# Multiple Autopolyploid *Arabidopsis lyrata* Populations Stabilized by Long-Range Adaptive Introgression Across Eurasia

**DOI:** 10.1093/molbev/msaf153

**Published:** 2025-07-24

**Authors:** Alison D Scott, Uliana K Kolesnikova, Anna Glushkevich, Laura Steinmann, Nikita P Tikhomirov, Ursula Pfordt, Magdalena Bohutínská, Robin Burns, Alexey P Seregin, Filip Kolar, Roswitha Schmickl, Polina Yu Novikova

**Affiliations:** Department of Chromosome Biology, Max Planck Institute for Plant Breeding Research, Cologne 50829, Germany; Department of Chromosome Biology, Max Planck Institute for Plant Breeding Research, Cologne 50829, Germany; Department of Chromosome Biology, Max Planck Institute for Plant Breeding Research, Cologne 50829, Germany; Department of Chromosome Biology, Max Planck Institute for Plant Breeding Research, Cologne 50829, Germany; Department of Chromosome Biology, Max Planck Institute for Plant Breeding Research, Cologne 50829, Germany; Department of Chromosome Biology, Max Planck Institute for Plant Breeding Research, Cologne 50829, Germany; Department of Botany, Faculty of Science, Charles University, Prague, Czech Republic; Institute of Botany, The Czech Academy of Sciences, Průhonice, Czech Republic; Department of Plant Sciences, University of Cambridge, Cambridge, UK; Herbarium (MW), Faculty of Biology, M. V. Lomonosov Moscow State University, Moscow 119991, Russia; Department of Botany, Faculty of Science, Charles University, Prague, Czech Republic; Institute of Botany, The Czech Academy of Sciences, Průhonice, Czech Republic; Department of Botany, Faculty of Science, Charles University, Prague, Czech Republic; Institute of Botany, The Czech Academy of Sciences, Průhonice, Czech Republic; Department of Chromosome Biology, Max Planck Institute for Plant Breeding Research, Cologne 50829, Germany

**Keywords:** *Arabidopsis lyrata*, introgression, polyploid, adaptation

## Abstract

Abundance of established polyploid lineages varies across lineages, evolutionary time, and geography, suggesting both genetics and environment play a role in polyploid persistence. We show *Arabidopsis lyrata* is the most polyploid-rich species complex in the *Arabidopsis* genus, with multiple origins of autotetraploidy. This is revealed by genomic data from over 400 *A. lyrata* samples across Eurasia. We found over 30 previously undescribed autotetraploid populations in Siberia with a minimum of two separate origins, independent of those previously reported in Central Europe. The establishment of Siberian tetraploids is mediated by meiotic adaptation at the same genes as in European tetraploid *A. lyrata* and *Arabidopsis arenosa,* despite their genomic divergence and geographical separation. Haplotype analysis based on synthetic long-read assemblies supports the long-range introgression of adaptive alleles from the tetraploid interspecific pool of European *A. lyrata* and *A. arenosa* to tetraploid Siberian *A. lyrata*. Once adaptations to polyploidy emerge, they promote the establishment of new polyploid lineages through adaptive inter- and intraspecific introgression.

## Introduction

Polyploidization cycles are recurrent and abundant with non-random distribution in space, through time, and across the Tree of Life. Polyploid frequency has a latitudinal gradient: more polyploid lineages are observed in colder regions closer to the poles (Rice et al. 2019; David 2022), which is paralleled with dating of ancient whole-genome duplication (WGD) events at climatically harsh time periods (Van de Peer et al. 2017). Whole genome duplications are recurrent; most major lineages of flowering plants experienced multiple polyploidy events detected in the period between 75 and 55 Mya (Lohaus and Van de Peer 2016), a time range of drastic climatic changes. Polyploidy is more abundant in plants (Barker et al. 2016; Van de Peer et al. 2017) compared to animals (Gregory and Mable 2005), in angiosperms versus gymnosperms (Khoshoo 1959), and in amphibians versus mammals (Gregory and Mable 2005; Schmid et al. 2015). What explains the abundance of polyploids in certain lineages, their emergence at certain times and climates? The explanation of the non-random distribution of polyploidy must lie in the balance between the birth and death rate of whole genome duplications, which differs depending on the context. For example, the environment, which varies across both time and space, can trigger polyploidy through unreduced gamete production (De Storme et al. 2012; Mason and Pires 2015; Zhou et al. 2015) and impact extinction rates (Van de Peer et al. 2017). Genetic background also affects polyploid birth rates directly through production rates of unreduced gametes (Mason et al. 2011; Kreiner et al. 2017a, 2017b), and can provide a route to frequent polyploid emergence, as suggested by theoretical modeling (Kauai et al. 2023). Studies from numerous plant systems show multiple origins of polyploidy, further suggesting particular genetic backgrounds provide ample opportunity for polyploid emergence (Soltis et al. 2009), including in autopolyploids (Servick et al. 2015).

The emergence of polyploids does not guarantee their survival. Neopolyploid establishment includes adaptation to external environments (Manzaneda et al. 2012; Chao et al. 2013), but adaptation to the new environment within the nucleus is even more critical. WGD in an individual (or single lineage; autopolyploidy) results in multiple sets of homologous chromosomes. Challenges in executing faithful segregation of the doubled chromosomes during meiosis can hinder the survival of neopolyploids (Bomblies et al. 2015, 2016). Artificially induced polyploids have reduced fertility and show high levels of aneuploidy in subsequent generations (Comai et al. 2000; Yant et al. 2013; Burns et al. 2021; Katche et al. 2023), suggesting that natural polyploids have evolved adaptations to the polyploid state (Morgan et al. 2020; Bohutínská et al. 2021b) or may be established only from certain preadaptive combinations of alleles in the diploid pool (Katche et al. 2023).

The genus *Arabidopsis* is a prime example of the non-random nature of polyploidy, as the majority of diploid *Arabidopsis* species contribute to either allo- or auto-polyploid lineages. There are allopolyploid species *Arabidopsis suecica* (parents *Arabidopsis arenosa* and *Arabidopsis thaliana*) and *Arabidopsis kamchatica* (parents *A. lyrata* and *Arabidopsis halleri*), and both *A. lyrata* and *A. arenosa* harbor autotetraploid populations.

*Arabidopsis* polyploids emerged in times of climatic upheaval (Novikova et al. 2018). *Arabidopsis arenosa* polyploids have been described to have a single geographical origin in the western Carpathian Mountains and spread across Europe after a whole-genome duplication (Arnold et al. 2015; Monnahan et al. 2019). Establishing tetraploid *A. arenosa* involved polygenic adaptation of reproductive machinery, including pollen tube growth (Westermann et al. 2024) and meiosis (Yant et al. 2013; Bomblies et al. 2016; Morgan et al. 2020). The former may be required to restore ion homeostasis, and the latter to prevent entanglement between three or more chromosomal copies and ensure faithful segregation.

We focus our study on the northernmost *Arabidopsis* species with a wide geographical distribution—*Arabidopsis lyrata*. Multiple tetraploid *A. lyrata* populations have been described in Europe in several areas in the Czech Republic and Austrian Alpine foothills (Polatschek 1966; Ansell et al. 2010; Jørgensen et al. 2011; Schmickl and Koch 2011). Tetraploid *A. lyrata* populations in Central Europe form three lineages occupying three distinct regions (Bohutínská et al. 2023), which may suggest multiple origins of polyploidy, in contrast to a single tetraploid origin in *A. arenosa* (Arnold et al. 2015). Diploid and tetraploid *A. lyrata* and *A. arenosa* have overlapping, yet nonsympatric ranges in Europe, and while diploids are reproductively isolated (Lafon-Placette et al. 2017) and do not show signs of ongoing interspecific gene flow (Clauss and Mitchell-Olds 2006; Ross-Ibarra et al. 2008; Ansell et al. 2010; Schmickl and Koch 2011), tetraploid *A. lyrata* and *A. arenosa* often hybridize (Jørgensen et al. 2011; Schmickl and Koch 2011; Lafon-Placette and Kohler 2016; Hohmann and Koch 2017; Schmickl et al. 2017; Marburger et al. 2019). Interestingly, interspecific gene flow between European tetraploid *A. lyrata* and *A. arenosa* has been adaptive: several introgressed alleles of meiotic genes from *A. arenosa* are also under selection in tetraploid *A. lyrata*, as they increase the fitness of tetraploids by stabilizing chromosomal segregation (Marburger et al. 2019; Schmickl and Yant 2021).

There are indications of a polyploid population in Yakutia (Zhukova et al. 1973; Novikova et al. 2018), suggesting that ploidy variation could still be massively underestimated in this species due to a lack of extensive sampling, particularly in Northern Eurasia. Despite the wide distribution of *A. lyrata* across the Northern Hemisphere (Dart et al. 2004; Schmickl et al. 2010; Novikova et al. 2016; Walden et al. 2020), a large part of the described range from Eastern Europe to Eastern Siberia lacks genetic data. We generate a whole-genome re-sequencing dataset from this understudied region and ask if polyploids are abundant there. Here we report multiple new tetraploid *A. lyrata* populations in the areas of White Sea shore, Polar Ural, and Central Siberia. We describe the nature, origins, and relationships of these WGDs. We ask what explains the abundance of polyploids in *A. lyrata* in these remote geographical regions since the distribution of previously described adapted polyploids is limited to European *A. arenosa* and *A. lyrata*. We investigate if any of the alleles known to stabilize polyploid meiosis could reach from Europe to Eastern Siberia or adaptation to polyploidy is purely convergent.

## Results

### Tetraploid *A. lyrata* is Abundant in Siberia

**Fig. 1.**
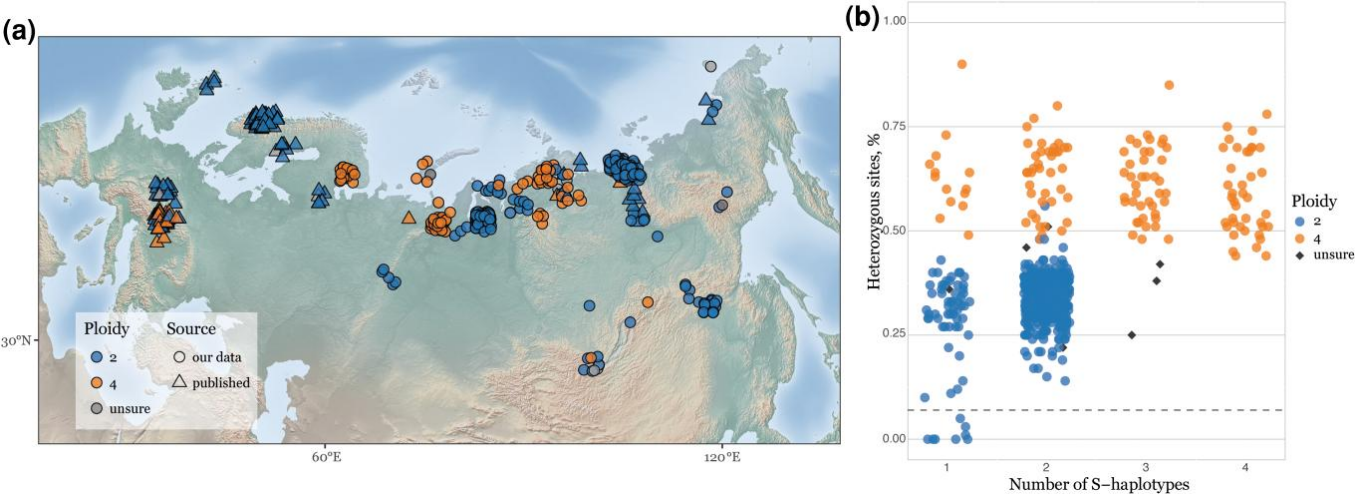
Distribution of diploid and tetraploid *A. lyrata* across Eurasia. a) Geographical distribution of *A. lyrata* samples used in this study. For better visualization in cases of overlaps we randomly shifted the points within 100 km from the actual sampling location, all true coordinates can be found in the supplementary data S1, Supplementary Material online. Newly found tetraploid *A. lyrata* populations are represented in orange circles. b) Relationships between proportions of heterozygous biallelic SNPs and the number of different *S*-alleles in each individual shown in (a). Tetraploid individuals (orange dots) show a higher proportion of heterozygous sites and up to four different *S*-alleles. Diploid individuals (blue dots) show a lower proportion of heterozygous sites and up to two different S-alleles; individuals with one *S*-allele below the dashed gray line correspond to recently described Siberian selfing *A. lyrata* lineage with nonfunctional *S*-locus (Kolesnikova et al. 2023). Herbarium individuals with ambiguous assignments of ploidy between two inference methods are shown in gray dots.

We assembled a dataset of whole-genome sequences from several sources. First, we sequenced 116 herbarium samples from the Moscow University Herbarium (Seregin 2025), spanning a wide geographical area from the White Sea to the Bering Strait. Second, we sequenced several individuals per population (283 samples from 32 locales) grown from seeds collected during expeditions to the White Sea, Polar Urals, Gydan Peninsula, Putorana Plateau, Lower Lena, and Amur basin (Fig. 1a, supplementary fig. S1, Supplementary Material online, supplementary data S1, Supplementary Material online). Third, we included whole-genome sequencing data of samples from different *A. lyrata* lineages which have been previously published (Novikova et al. 2016; Hämälä et al. 2017; Mattila et al. 2017; Marburger et al. 2019; Takou et al. 2021; Willi et al. 2022). All re-sequencing data were mapped to the reference genome of the self-compatible Siberian *A. lyrata* NT1 strain (Kolesnikova et al. 2023).

We used a statistical framework based on relative allelic counts implemented in nQuire software (Weiß et al. 2018) to get a first inference of ploidy from sequencing data (see Materials and Methods). We initially called variants for all samples as diploids. The proportion of heterozygous sites in tetraploid individuals was generally higher than in the diploids (Fig. 1b, supplementary fig. S2, Supplementary Material online). To complement the initial inference, we genotyped and counted *S*-alleles in each sample using NGSgenotyp (Genete et al. 2020). Due to the extreme divergence and diversity of *S*-alleles controlling self-incompatibility in *Arabidopsis* (Charlesworth et al. 2003; Mable et al. 2003, 2004; Castric and Vekemans 2004; Castric et al. 2008; Llaurens et al. 2008; Le Veve et al. 2023), it was often possible to find up to two different *S*-alleles in diploid and up to four different *S*-alleles in tetraploid individuals. These independent assessments of ploidy levels allowed us to infer ploidy for most of the samples (Fig. 1b). Finally, we directly counted chromosome numbers in 18 live samples grown in the greenhouse from seeds collected in the field (representative images in supplementary fig. S1, Supplementary Material online and supplementary data S2, Supplementary Material online) and confirmed our bioinformatic inferences. When bioinformatic inference yielded unclear results and cytogenetics was not possible (i.e. for herbarium specimens), we indicated the ploidy as “unsure” and excluded these accessions from downstream analyses—overall, 11 samples were excluded, representing ∼2.5% of new accessions (Fig. 1b, supplementary data S1, Supplementary Material online). Also, while tetraploid and diploid populations sometimes occur nearby, we found no evidence of mixed-ploidy populations: all individuals sampled from the same population had consistent ploidy levels (supplementary data S1, Supplementary Material online). Following the assignment of ploidy by heterozygosity, *S*-allele genotype, and cytological confirmation, we re-called variants according to the inferred ploidy state using the designated ploidy-aware approach in GATK.

Our live collections included confirmed self-compatible and self-incompatible diploid *A. lyrata* individuals, which differ in the levels of heterozygosity (Kolesnikova et al. 2023) (Fig. 1b, supplementary fig. S2, Supplementary Material online). Tetraploid individuals had a higher proportion of heterozygous sites compared to diploid selfers and outcrossers, and up to four different S-alleles in each individual (Fig. 1b, supplementary fig. S2, Supplementary Material online), pointing to their outcrossing nature, consistent with self-incompatibility observed in the greenhouse. While nucleotide diversity (π) calculated on putatively neutral four-fold degenerate sites is comparable (∼0.004) between diploid and tetraploid *A. lyrata* lineages in Siberia (supplementary fig. S3, Supplementary Material online), a *t*-test comparing nucleotide diversity between diploid and tetraploid lineage pairs shows that π is significantly higher in Northern Ural (NU) tetraploids versus diploids (supplementary fig. S4, Supplementary Material online). The same comparison for Central Siberian (CS) diploids and tetraploids yields no significant difference. While the theoretical expectation is that π in tetraploids should be twice as high as their diploid ancestors, our observations are consistent with recent work showing that nucleotide diversity in tetraploids takes millions of generations to reach twice that of diploids (Vlček et al. 2025) We additionally calculated Tajima's D (Tajima 1989), which is more strongly negative in tetraploid populations than diploid populations (supplementary fig. S4, Supplementary Material online), suggesting tetraploid lineages may still be rebounding from a historical bottleneck.

### Independent Autopolyploidization Events of *A. lyrata* in NU and Central Siberia Around Last Glacial Maximum

**Fig. 2.**
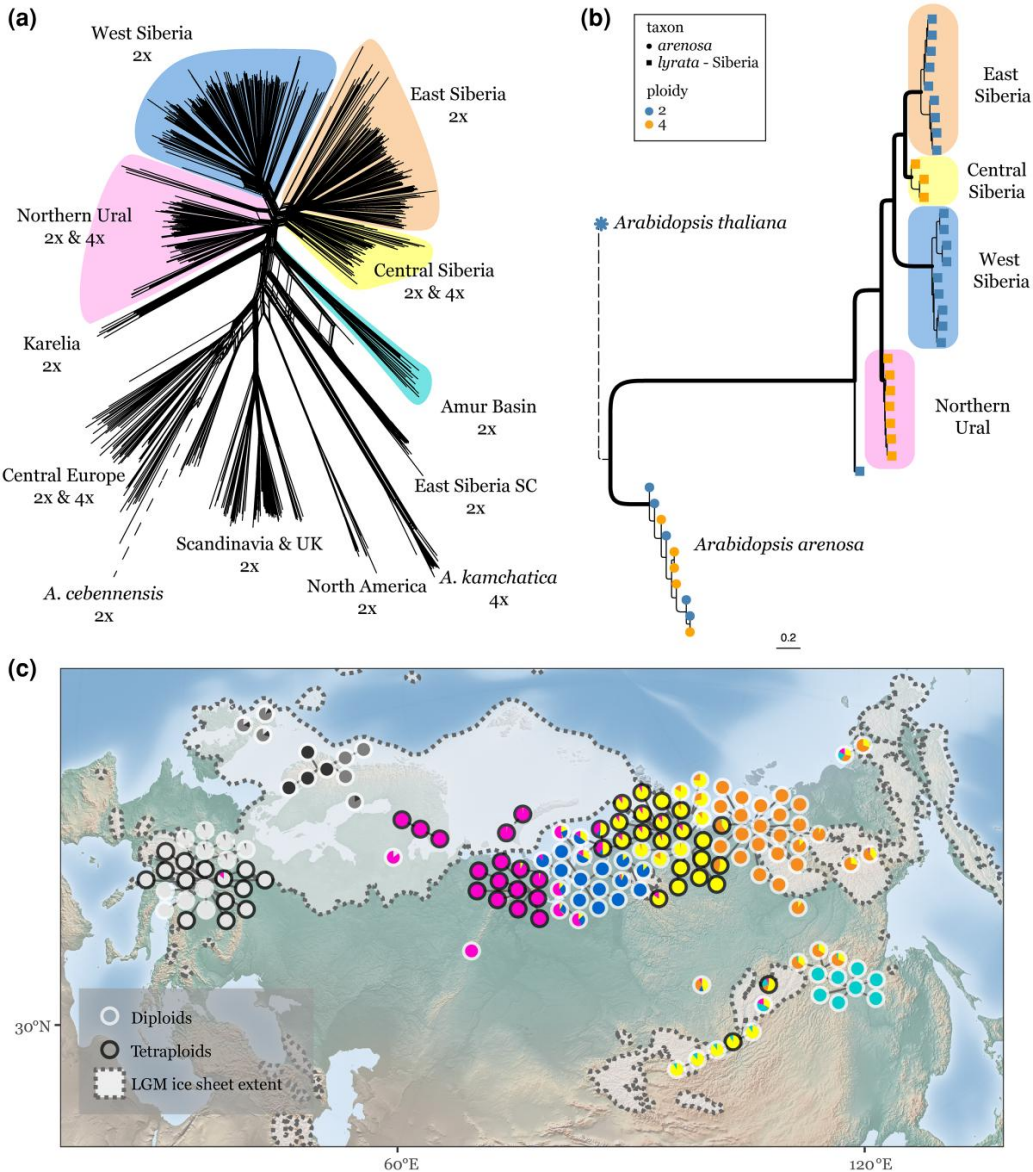
Population structure of *A. lyrata* diploid-tetraploid species complex. a) Network representation of genetic pairwise distances (Nei's D) between individuals. *Arabidopsis lyrata* accessions form several clusters outside of Central Europe, named here as NU (pink, diploid/tetraploid), WS (blue, diploid), CS (yellow, diploid/tetraploid), ES (orange, diploid), and Amur Basin (AB; turquoise, diploid). Note that while network clusters may contain multiple ploidy levels, geographic populations are not mixed ploidy. Newly described autotetraploids are found in two distinct clusters (NU/pink and CS/yellow), distinct from Central European autotetraploids (gray), and allotetraploid *A. kamchatica*. b) ASTRAL summary phylogeny of 4,000 gene trees from stLFR assemblies of *A. lyrata* and PacBio assemblies of *A. arenosa*, with *A. thaliana* as outgroup. Each tip is an accession, shape reflects taxon (square = *A. lyrata*, circle = *A. arenosa*) and color denotes ploidy (blue = diploid, orange = tetraploid). Thicker branches have a quartet support of 1.00. Branch lengths reported in coalescent units. Dotted branch subtending the outgroup has been shortened for easier plotting. Color of each clade is consistent with labeling in (a). c) Admixture across the Eurasian *A. lyrata* distribution. Colors correspond to labeling in (a) and (b) and reflect average admixture proportions per sampling site. Tetraploid accessions are found across the range (denoted by black outlines). Extent of the ice sheet during the LGM (Ehlers et al. 2011) shown in white.

To characterize the population structure of *A. lyrata* across Eurasia, we calculated pairwise genetic distances from the four-fold degenerate sites as a proxy for nearly neutral sites and built a network from the resulting distance matrix (Fig. 2a), showing that the newly described tetraploids cluster within two distinct groups, separate from the Central Europe tetraploids. NU (highlighted in pink, Fig. 2a) populations sampled around the White Sea and in the Polar Ural Mountains form a cluster containing diploid and tetraploid individuals. This NU cluster is genetically close to the West Siberian (WS) cluster (WS, highlighted in blue, Fig. 2a) comprised purely of diploid populations sampled from the Taz Estuary. The CS cluster (highlighted in yellow, Fig. 2a) also contains both diploids and tetraploids and, in turn, is closer to diploids from the lower Lena river area in East Siberia (ES, highlighted in orange, Fig. 2a). We also used the distance matrix to estimate a UPGMA clustering tree to validate the identity of *A. lyrata* samples (supplementary fig. S5, Supplementary Material online), which confirmed our taxon assignments.

We estimated gene trees for representatives of the Siberian *A. lyrata* lineages and the resulting phylogenetic summary from ASTRAL (Fig. 2b) clarifies relationships among diploids and tetraploids. Tetraploids from Central Siberia (yellow) are most closely related to diploids from ES (orange), and these two lineages form a clade with WS diploids (blue). NU tetraploids (pink) are sister to the clade of Central Siberia-East Siberia-West Siberia. The self-compatible Eastern Siberia diploid lineage falls outside the rest of the Siberian clade, consistent with its position on the distance matrix in Fig. 2a. Diploid and tetraploid accessions of *A. arenosa* form an independent clade, separate from *A. lyrata*. Overall, the tree topology supports two independent tetraploid origins of *A. lyrata* in Siberia and suggests the Northern Ural (NU) tetraploids are equally closely related to WS diploids, East Siberian diploids, and CS tetraploids.

Using population-level data, we estimated site frequency spectra (SFS) for the two newly discovered tetraploid *A. lyrata* lineages (NU 4× and CS 4×) separately and compared them to theoretical expectations for auto- and allo-polyploids. In both cases, the observed SFS of Siberian *A. lyrata* tetraploids are consistent with autopolyploidy, indicated by an absence of sites at an intermediate allele frequency (supplementary fig. S6, Supplementary Material online). Allotetraploid populations exhibit a pronounced peak at the intermediate frequency when mapped to a diploid reference due to subgenome divergence, while autotetraploid populations do not show such a peak (Blischak et al. 2023); however, allotetraploids may lack a peak due to, e.g. multisomic inheritance or widespread homeologous exchange. Allotetraploid *Arabidopsis kamchatica*, originating through hybridizations of Siberian *A. lyrata* and *A. halleri* (Shimizu et al. 2005; Shimizu-Inatsugi et al. 2009; Kolesnikova et al. 2023), is also included in the network and clusters separately from NU and CS autotetraploid *A. lyrata* (Fig. 2a).

We then used TreeMix (Pickrell and Pritchard 2012) to describe relationships among diploid populations. The maximum likelihood model (supplementary fig. S7a, Supplementary Material online) groups Karelia, NU, Western Siberia, Central Siberia, Eastern Siberia, and AB together with suggested migration edge between Karelia and Central Europe, while UK and Scandinavian populations grouped with Central European populations. In the maximum likelihood model including all diploid and tetraploid populations, neither European nor Siberian tetraploids grouped together; in both cases instead grouping with geographically close diploids (supplementary fig. S7b, Supplementary Material online). We also ran TreeMix using the diploid and tetraploid Siberian lineages from phylogenetic analysis in Fig. 2b, and the resulting tree recapitulates the ASTRAL summary phylogeny, with the addition of a migration edge between the tetraploid populations (supplementary fig. S7c, Supplementary Material online). Clustering of individuals using ADMIXTURE (Alexander et al. 2009) with the optimal number of groups *K* = 8 (supplementary fig. S8, Supplementary Material online) assigned tetraploid and diploid NU populations into a single cluster (pink, Fig. 2c) with high levels of admixture with diploid WS populations (blue, Fig. 2c); tetraploids and diploids from CS were also assigned into a single cluster (yellow, Fig. 2c) with high levels of admixture with diploid ES cluster (orange, Fig. 2c). We used topology weighting with Twisst (Martin and Van Belleghem 2017) to further explore the relationships between *A. lyrata* lineages when variation in topologies across the genome is taken into account (supplementary fig. S9, Supplementary Material online). The top three highest-weighted topologies are consistent with the independent origin of the two autotetraploid lineages.

To test whether the tetraploids in Siberia have a single origin or independent origins, we used a demographic modeling approach based on fitting simulated and observed SFS (Excoffier et al. 2021). We used four diploid and tetraploid populations (NU 2× and NU 4×, CS 2× and CS 4×) and tested simple divergence models with different topologies, alongside models allowing gene flow between tetraploids and from diploids into tetraploids (supplementary fig. S10, Supplementary Material online). Among all the tested models, likelihood comparisons and AIC supported an independent origin model which recapitulates the ASTRAL summary tree in Fig. 2b (supplementary fig. S10, model c, Supplementary Material online; supplementary table S3, Supplementary Material online). While we exercise caution in the interpretation of parameter estimates, the origin of CS tetraploids is estimated to be much younger than NU tetraploids (∼4,000 generations vs. ∼30,000 generations; supplementary table S4, Supplementary Material online). We also tested demographic models to compare relative origin times of Central European and NU tetraploids, with resulting divergence time estimates suggesting Central European tetraploids are older by at least an order of magnitude than NU tetraploids (supplementary table S5, Supplementary Material online).

### Tetraploid-Specific Adaptive Introgression of Genes Involved in Meiotic Stabilization

**Fig. 3.**
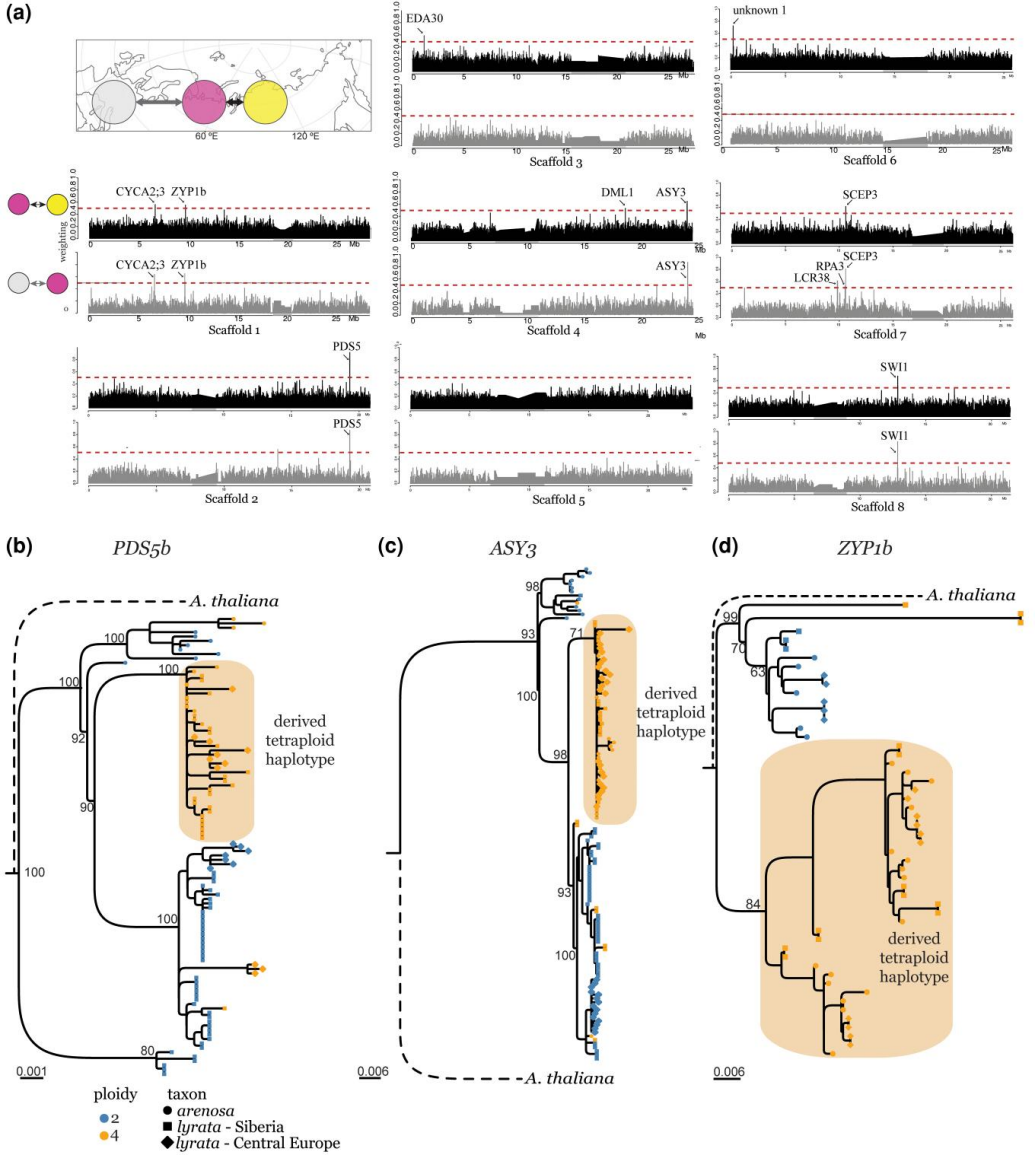
Genes present in introgression topologies. a) Map showing tetraploid lineages as circles, Grey = Central Europe, Pink = Northern Ural, Yellow = Central Siberian. Each plot is a distribution of introgression topology windows along a chromosome, with upper panels (black) showing introgression windows between NU and CS tetraploids, and lower panels (gray) showing introgression windows between Central Europe and NU tetraploids. Height of peaks indicates topology weighting, and introgression blocks are annotated. Blocks may contain multiple gene models, see supplementary data S4, Supplementary Material online for full list. Red dashed line indicates 50% weight. b to d) gene trees for haplotypes from *A. lyrata* in Siberia, *A. lyrata* in Central Europe, and *A. arenosa*. Each tip is a haplotype, orange tips are haplotypes from tetraploid individuals, and blue tips from diploids. Shape indicates taxon: squares for *A. lyrata* in Siberia, diamonds for *A. lyrata* in Central Europe, and circles for *A. arenosa*. Orange box highlights derived tetraploid haplotype clade (b) Haplotype tree for *PDS5b* (c) Haplotype tree for *ASY3* (d) Haplotype tree for *ZYP1b*.

Topology weighting results showed that topologies consistent with the ASTRAL tree and demographic analysis were the most frequent (supplementary fig. S9, Supplementary Material online), but a smaller but considerable fraction indicated introgression between the NU and CS tetraploids (topologies 7, 8, 9, supplementary fig. S9, Supplementary Material online). We combine the topology weighting results for all introgression topologies and show them along the chromosomes (Fig. 3a, upper panels in black). In total, there are 11 windows with a high weighting of gene flow topologies (introgression windows), visible as sharp peaks. Comparing these window boundaries with gene annotation, we identified 37 gene models (supplementary data S4, Supplementary Material online) located within introgression windows. These genes are enriched for gene ontology terms related to meiosis and chromosome organization (supplementary fig. S11 and data S4, Supplementary Material online). Performing the same analysis to identify introgression windows using Central European and NU diploid and tetraploid populations, we find striking overlap of the introgression windows found in the NU and CS comparison (Fig. 3a, lower panels in gray). For each gene within introgression windows, we calculate SNP frequencies across diploid and tetraploid lineages including Central Europe, NU, and CS and visualize frequencies as heatmaps (supplementary fig. S12, Supplementary Material online). We see SNP sharing across the entire sampled range, from Central Europe to Eastern Siberia: in some cases SNPs appear to be tetraploid-specific (e.g. *ZYP1b*, *ASY3*, *MAU2*, and *PDS5*, supplementary fig. S12, d, m, ee, ff, Supplementary Material online) and other SNPs are shared among diploids and tetraploids (e.g. in *CYCA2;3* and *PAPP2C*, supplementary fig. S12, a and c, Supplementary Material online).

Upon seeing which genes fell within introgression windows, we tested enrichment for gene ontology terms among the 37 introgressed genes. The strong enrichment of meiosis-related gene ontology terms (supplementary fig. S11, Supplementary Material online) raises the question of whether introgressed alleles may be undergoing positive selection. To explore this idea, we calculated nucleotide diversity (π) per gene within NU tetraploids and between NU diploids and tetraploids and between CS diploids and tetraploids (dxy) using piawka (https://github.com/novikovalab/piawka) and calculated residuals. This “diversity versus differentiation” metric allows us to identify genes that are more differentiated than expected given their within-population diversity (Yant et al. 2013), which could indicate positive selection acting in the tetraploid population (supplementary figs. S13 and S14, Supplementary Material online). Four genes from the introgression windows also fall among outliers in the residuals analysis (supplementary fig. S14, Supplementary Material online), including meiosis genes *ASY3* and *PDS5*, suggesting these loci may be experiencing selection.

Given that introgression genes are enriched for meiotic functions and some of these loci may be under selection, we moved beyond the SNP dataset to examine haplotype sharing across the range of autopolyploid *Arabidopsis lyrata* and *A. arenosa*. We used full haplotype sequences from synthetic long reads (single-tube long fragment reads; stLFR), PacBio long-read assemblies (see Materials and Methods), and published haplotype sequences of Sanger sequenced PCR products (Morgan et al. 2020; Seear et al. 2020) in *A. lyrata* and *A. arenosa*. We focused on three introgressed genes (*ASY3*, *PDS5*, and *ZYP1b*) with substantial published sequence data across *Arabidopsis* genus allowing haplotype-scale alignments for further analyses. We aligned assembled haplotypes from diploid and tetraploid *A. arenosa* and *A. lyrata* and estimated phylogenetic trees. Tetraploid-specific haplotypes from *A. arenosa*, Central European, NU, and CS *A. lyrata* form a monophyletic clade at these loci (Fig. 3b-d, orange clade). While the derived tetraploid haplotype is only found in tetraploids and never in diploids, tetraploids can harbor diploid haplotypes as well. This corresponds well with SNP frequency heatmaps at the same loci (supplementary fig. S11, d, m, and ff, Supplementary Material online), but is a different topology than the ASTRAL summary tree which is comprised of loci lacking introgression signatures (Fig. 2b).

## Discussion

Our range-wide sampling of *A. lyrata* across Eurasia reveals the extent of *A. lyrata* tetraploids (Fig. 1), which was dramatically underestimated until now. Here, we report tetraploids from 30 locations and classify them as autopolyploids, which is apparent from both their deep clustering within *A. lyrata* (Fig. 2) and the absence of fixed heterozygosity due to subgenome divergence (supplementary fig. S6, Supplementary Material online). Autotetraploid populations from the NU region cluster separately, sister to the rest of the Siberian samples, while CS tetraploids are most closely related to local diploids (Fig. 2, supplementary fig. S4b, Supplementary Material online), suggesting different source populations for tetraploid origins. Considering that autotetraploid *A. lyrata* populations in Europe form distinct lineages, perhaps from multiple origins (Bohutínská et al. 2023), and that allotetraploid *A. kamchatica* originated from multiple hybridizations between *A. lyrata* and *A. halleri* (Shimizu-Inatsugi et al. 2009; Schmickl et al. 2010), we conclude that *A. lyrata* is the most polyploid-rich species complex within *Arabidopsis* genus in both auto- and allopolyploid context. The recurrent formation of tetraploids may imply *A. lyrata* (i) has a propensity for WGD, perhaps by an increased likelihood of producing unreduced gametes or increased sporadic somatic WGD and (ii) establishment of *A. lyrata* tetraploids is facilitated by special environmental conditions or genetic background.

*Arabidopsis lyrata* is the northernmost species in the genus, and its evolutionary history shaped by temperature fluctuations during the Pleistocene (Schmickl et al. 2010). Expansion of *A. lyrata* eastwards from Europe may predate the second to last Ice Age (Ross-Ibarra et al. 2008), which peaked at 130 Kya. Pleistocene temperature oscillations and glaciation cycles explain the main migration routes and secondary contacts in refugia areas, such as Beringia (Schmickl et al. 2010). TreeMix analysis grouped Siberian and Karelian populations together, while UK and Scandinavian populations grouped with Central European populations, consistent with previously suggested different colonization routes for Karelia and Scandinavia, with the former being colonized from NU and the latter from Central Europe, both occurring after the ice sheet retreat post-LGM (Muller et al. 2008; Mattila et al. 2017). Such a pattern of distinct recolonization routes has also been observed in *A. thaliana* (The 1001 Genomes Consortium 2016*)*, and other Arctic species including keystone species such as *Dryas octopetala, Vaccinium vitis-idaea*, and *Betula nana* (Eidesen et al. 2013).

Given that *A. lyrata* appears to be the most polyploid-rich taxon in the *Arabidopsis* genus, we again ask: what explains the abundance of polyploids in certain lineages, their emergence at certain times and climates? Our estimations of the divergence times between the diploids and tetraploids in NU and Central Siberia arrive close to the peak of the last Ice Age (supplementary table S4, Supplementary Material online) and follow a general pattern of co-occurrence with glaciations (Novikova et al. 2018). This suggests that environment may play a crucial role in polyploid birth rate, triggering whole-genome duplications via production of 2× gametes: either unreduced gametes formed in a diploid, or normal gametes of a somatically doubled branch. Environmental stress can disturb microtubules and prevent the completion of meiotic cell division (Mason et al. 2011; Sora et al. 2016).

However, as the emergence of polyploids does not guarantee their survival, what contributes to the reduced death rate in Siberian tetraploid lineages? While environmental disturbance may also facilitate establishment of polyploids, e.g. by opening novel space for colonization after the ice-sheet retreat (Brochmann et al. 2004; Parisod et al. 2010), we see strong evidence of the impact of genetic adaptation. Topology weighting analysis and haplotype sharing from de novo assemblies reveal introgression among distinct *A. lyrata* tetraploid lineages over long distances (Fig. 3). Tetraploid populations in Europe were dated at around 160 Kya (Marburger et al. 2019) and in our demographic modeling results are older than Siberian tetraploid lineages by at least an order of magnitude. Given the colonization history of A. lyrata moving from west to east, we infer the direction of the gene flow from Central European *A. lyrata* all the way to Central Siberian *A. lyrata* (Fig. 3). The interspecific introgression from tetraploid *A. arenosa* to tetraploid *A. lyrata* in Central Europe has been shown previously (Marburger et al. 2019). Here, we show that genomic regions introgressed between Central Europe/NU and NU/Central Siberia are narrow and highly enriched in meiotic genes (Fig. 3a, supplementary fig. S11, Supplementary Material online). Among introgressed haplotypes in Siberia, we find the *ASY3* tetraploid-derived allele which has an established functional role (Morgan et al. 2020). In natural tetraploid *A. arenosa*, plants with the derived tetraploid *ASY3* allele in conjunction with a derived *ASY1* allele have fewer multivalents and shorter meiotic axes than plants with the diploid alleles (Morgan et al. 2020), leading to more stable meiosis. Additional research (Seear et al. 2020) on *ASY3* as an adaptation candidate showed an increase in meiotic stability associated with the derived tetraploid *ASY3* haplotype. During meiosis, the length of the chromosomal axis dictates crossover frequency and position (Song et al. 2021; S. Wang et al. 2019). In tetraploids, axis length may be especially important if it serves to limit crossovers to one per chromosome, reducing the risk of multivalent formation (Morgan et al. 2021). Patterns of diversity and divergence in Siberian lineages at the introgressed region harboring *ASY3* gene are consistent with positive selection, suggesting that introgression was adaptive.

Similar patterns of both long-range introgression and positive selection in *A. lyrata* polyploids across Eurasia are observed in the region harboring the cohesin cofactor PDS5. In budding yeast, PDS5 supports sister chromatid cohesion, as mutants suffer from early separation of sister chromatids (Zhang et al. 2005). While experimental work in *A. thaliana* shows that PDS5 mutants have minimal meiotic phenotypes (Pradillo et al. 2015), suggesting its role in *Arabidopsis* diverges from that in yeast systems, recent work shows that PDS5 impacts axis length in multiple model systems (Viera et al. 2020; Song et al. 2021; Joo et al. 2022). As described above for ASY3, regulation of axis length in tetraploids can help to stabilize meiosis, as a shorter axis can limit crossover number. *PDS5* and its paralogs were repeatedly found in selection scans between diploid and tetraploid lineages in Brassicaceae: first in *A. arenosa* (Hollister et al. 2012; Yant et al. 2013; Wright et al. 2015), then in Central European *A. lyrata* (Marburger et al. 2019), and in *Cardamine amara* (Bohutínská et al. 2021a), where tetraploids harbor a private derived allele, absent from diploid gene pools. We find a high frequency of described adaptive tetraploid alleles in *ASY3* and *PDS5* meiotic genes in NU and CS tetraploids, which suggests that meiotic stabilization is required for polyploid establishment, and that this stabilization is primarily achieved through adaptive introgression.

Siberian *A. lyrata* tetraploids are younger compared to European and it is possible that meiotic stabilization there is incomplete. For example, we find synaptonemal complex protein ZYP1b in introgression scans across Eurasia. The paralogs *ZYP1a* and *ZYP1b* encode transverse filament proteins which are central elements of the synaptonemal complex, helping to “zip” together chromosomal axes of homologs, distribute crossovers and establish proper bivalents (Higgins et al. 2005; Barakate et al. 2014; Capilla-Pérez et al. 2021). Tetraploid alleles of *ZYP1* paralogs are likely adaptive as they appear on introgression and selection scans (Yant et al. 2013; Wright et al. 2015; Marburger et al. 2019). However, in Siberia, although tetraploid Central European alleles of *ZYP1b* are already introduced and detected by introgression scans, their frequency is intermediate (supplementary fig. S11d, Supplementary Material online) and *ZYP1b* does not appear among outliers in diversity and divergence patterns (supplementary fig. S13, Supplementary Material online). The varying patterns across meiotic loci is consistent with previous work showing meiotic adaptation to polyploidy is polygenic without a single key player but rather many players of cumulative effects (Bomblies et al. 2015; Bomblies 2023). Emphasizing the polygenic nature of meiotic adaptation to polyploidy, our introgression windows contain a gene model (SCEP3) recently described as a member of the synaptonemal complex which may work in concert with ZYP1 in crossover mediation (Heckmann et al. 2024).

It seems that, while environmental fluctuation may yield increased polyploid formation in *A. lyrata*, the persistence of these neopolyploids is due to genetic adaptation involving long-range introgression. This raises the question of how neopolyploids survived initially, without beneficial haplotypes in the population. In contrast to *A. arenosa*, *A. lyrata* is known to propagate clonally (via root sprouts), which could help local neopolyploid persistence in spite of initial unstable meiosis. Environmental conditions could also play a role in early stabilization. For example, it is known that temperature impacts meiosis by modifying the number of crossovers per chromosome (Morgan et al. 2017). It is plausible that vegetative propagation and environmental conditions allowed the initial survival of neopolyploid *A. lyrata* in Siberia. Then, gene flow into the nascent tetraploid populations from the established Central European gene pool introduced the adaptive alleles, enhancing further polyploid establishment and ultimately resulting in further persistence of polyploids in Siberia.

## Materials and Methods

### Fieldwork and Plant Collection

We used a combination of herbarium specimens and seeds collected from the field for this study. Herbarium samples were provided by the Moscow University Herbarium (MW), the Herbarium of Irkutsk State University (IRKU) and the herbarium of the Siberian Institute of Plant Physiology and Biochemistry. For herbarium accessions, we sampled a small amount of dried tissue from at least one individual plant on a herbarium sheet. Samples from a single collection locale were named using the herbarium accession appended by a number indicating multiple plants that were sequenced from a sheet (e.g. MW0079552_1, MW0079552_2 and MW0079552_3). We collected seeds from wild populations of *A. lyrata* in expeditions to the Lena River Valley, Taz Estuary, Polar Ural, and South Yakutia (supplementary data S1, Supplementary Material online). We then grew these seeds in the greenhouse (21 °C, 16 h of daylight) and sampled leaf tissue for sequencing.

### Short-Read Sequencing for Population Analyses

We used dried leaves either sampled from herbaria, the field, or live plants in the greenhouse. Plant material was processed in two different ways, indicated by types I and II in supplementary data S1, Supplementary Material online. Processing protocols for each type are described in detail in Kolesnikova et al. (2023). Briefly, we isolated DNA from type I herbarium samples using the DNeasy Plant Mini Kit (Qiagen) followed by library prep with the NEBNext DNA Library Prep Kit. For type II samples, we isolated DNA using the NucleoMag Plant” kit from Macherey and Nagel (Düren, Germany) on the KingFisher 96Plex device (Thermo), followed by a TPase-based library prep. Libraries were pooled and sequenced on the NovaSeq 6000 S4 flow cell Illumina system with paired-end 150 bp reads. The Full list of short-read sequenced samples used in the study is in the supplementary data S1, Supplementary Material online.

### Linked-Read Sequencing

DNA from 2 g of fresh material was isolated with a NucleoBond HMW DNA kit (Macherey Nagel). Quality, library preparation, and sequencing were performed by BGI as described in O. Wang et al. (2019). Library preparation process starts from random insertions of a transposon sequence into the long fragment DNA, then the product is combined with a magnetic bead carrying a multi-copy molecular barcode. Each DNA molecule receives a unique barcode. After that, fragments are amplified using PCR, and single-stranded cyclized products are used for pair-end sequencing DNBSEQ with read length of 100 bp.

### Short Read Mapping and Variant Calling for Population Analysis

We analyzed short paired-end reads (2×150 bp) the same way as in Kolesnikova et al. (2023). The reads were filtered for adapters with bbduk.sh script from BBMap (38.20) (Bushnell 2014) with ktrim = r k = 23 mink = 11 hdist = 1 tbo tpe qtrim = rl trimq = 15 minlen = 70. Then, we mapped all reads to the NT1 *A. lyrata* reference genome (Kolesnikova et al. 2023) using bwa mem (0.7.17) (Li and Durbin 2009) with -M parameter. We used Picard MarkDuplicates (Broad Institute) to mark PCR duplicated reads and then used Samtools (Li et al. 2009) to sort and index the BAM files. We called variants with HaplotypeCaller algorithm from GATK (3.8) (McKenna et al. 2010) and then combined individual vcf files using CombineGVCFs and created final vcf using GenotypeGVCF from GATK including non-variant sites with—includeNonVariantSites parameter. We estimated heterozygosity on a vcf with variants called for all samples as for diploids by calculating proportion of heterozygous sites among all called sites. For the further analysis, we created a vcf with variant and non-variant sites called according to the ploidy of the samples such that tetraploid individuals are genotyped as tetraploids mapped to a diploid reference (HaplotypeCaller with—sample-ploidy 2 or—sample-ploidy 4). Autotetraploids (*A. lyrata*) and allotetraploids alike (*A. kamchatica*) were mapped in full to the diploid reference and not split by subgenome.

### Synthetic Long-Read Assemblies and Tree Inference

StLFR reads were assembled using the stlfr2supernova_pipeline (https://github.com/BGI-Qingdao/stlfr2supernova_pipeline) which is based on Supernova (Weisenfeld et al. 2017) with—style = pseudohap2 option to get two haplotypes for each assembly. The haplotypes were scaffolded on the NT1 *A. lyrata* genome (Kolesnikova et al. 2023) with ragtag (Alonge et al. 2022). Long-read *A. arenosa* assemblies from (Bohutínská et al. 2024) were also scaffolded on *A. lyrata* reference and further used together with stLFR assemblies. The GALBA pipeline (Stanke et al. 2006; Buchfink et al. 2015; Hoff et al. 2019; Hoff and Stanke 2019; Li 2023) was used for protein annotation of the assemblies with proteins from Phytozome (Goodstein et al. 2012) *A. lyrata* v.2.1 release (Rawat et al. 2015) as a protein database. The annotated genes were used for gene tree construction. Protein BLAST (Camacho et al. 2009) was used to identify genes of interest in the annotation. Then, we used L-INS-i algorithm of MAFFT (Katoh et al. 2005) for multiple sequence alignment of the gene regions. The alignments were manually curated in UGENE (https://doi.org/10.1093/bioinformatics/bts091) and used for tree construction with iqtree (Nguyen et al. 2015; Hoang et al. 2018), fast bootstrap = 1,000.

For species tree inference from gene trees we used full-length BUSCO gene sequences (Simão et al. 2015). Predictions with the same ID from all the assemblies were aligned with L-INS-i after filtering for length to get rid of incomplete models. Gene trees produced by IQ-TREE were then summarized with wASTRAL (https://doi.org/10.1093/molbev/msac215) from the ASTER package. This method takes into account tree support and branch length values.

### Ploidy Estimation and *S*-Allele Genotyping From Short-Read Sequencing Data

To estimate ploidy, we used nQuire (Weiß et al. 2018) with “create,” “denoise,” and “lrdmodel” steps. This ploidy estimation method is based on allelic read depth distributions, also previously described (Scott et al. 2023). We identified *S*-alleles as described in Kolesnikova et al. (2023) using short-read sequencing data and *S*-locus genotyping pipeline NGSgenotyp (Genete et al. 2020) with two first steps: kmerfilter (–k 20 for *SRK* and –k 15 for *SCR*) and genotyp—with both *SRK* and *SCR* sequence reference databases. The databases used for genotyping also contain known *SRK-* and *SCR*-like, but unlinked genetic regions, that are used to normalize the coverage over the *S*-alleles and are later excluded from the genotyping results as false-positives. For every individual we used normalized *S*-allele coverage to make sure that all *S*-alleles were genotyped and count copy numbers for each *S*-allele. The number of different *S*-alleles in each individual together with proportion of heterozygous sites were used to confirm the estimated ploidy level. In tetraploid individuals with only two *S*-alleles the normalized depth over the S-alleles was sometimes unequal (3:1). This fact provided evidence that there were three copies of one allele and one copy of another and was also used to confirm ploidy. In addition to the databases used previously (Kolesnikova et al. 2023), we have assembled four *SRK* alleles from short-read data with assembly option and provide them together with 6 more published (Castric et al. 2008; Neuffer et al. 2023) *SRK* alleles in supplementary table S6, Supplementary Material online. The sequences of *S*-alleles are provided in the supplementary data S3, Supplementary Material online in .fasta format.

### Network Analysis and Population Admixture

To obtain a network of genetic distances between individuals, we followed Kolesnikova et al. (2023). Briefly, we filtered our biallelic vcf down to only fourfold degenerate sites. We then calculated Nei's genetic distance (D) (Nei 1972) on the filtered vcf in R (using StAMPP; Pembleton et al. 2013), and used SplitsTree4 (Huson and Bryant 2006) to visualize the resulting distance matrix. We ran a series of ADMIXTURE (Gutenkunst et al. 2009) analyses with *K* = 2 up to *K* = 15. Based on cross-validation error (supplementary fig. S8, Supplementary Material online), *K* = 8 was the optimal number of groups. To get population-level clustering, we combined clustering proportions for accessions from the same geographical coordinate and took the mean proportion. To accommodate for uneven sampling where some populations are represented by more individuals than others, we took three random representatives from populations with more than three accessions. Note that ADMIXTURE is not designed for mixed-ploidy datasets, and while there is the risk of spurious clustering by ploidy level rather than population (see Stift et al. 2019 for a thorough discussion), we do not see such a pattern with our dataset, instead observing clustering based on geography.

### Geographical Map

We used R v4.2.2(R Core Team 2022), primarily with the tidyverse v2.0.0 package collection (Wickham et al. 2019), for making the plots. Maps were created using Lambert Azimuthal Equal Area projection centered at N 40° E 90°, with packages sf v1.0-14 (Pebesma 2018) and raster v3.6-23 (Hijmans 2023) and a background map by NaturalEarth; the ggrepel algorithm (Slowikowski 2023) was used to adjust the positioning of the pie charts. The Last Glacial Maximum (LGM) ice sheet extents (Ehlers et al. 2011) were simplified for plotting.

### Cytogenetic Validation of Ploidy

To confirm ploidy levels estimated by bioinformatic methods, we used meiotic spreads. We first harvested immature buds (prior to pollen being formed) and fixed them in Carnoy fixative (3:1 ethanol:acetic acid) for 2 h on a horizontal shaker at room temperature, changed to fresh Carnoy fixative, and stored them at −20 °C until further usage.

Fixed buds were rinsed twice for 5 min in 10 mM citrate buffer (tri-sodium citrate, pH 4.5 with HCl), followed by a digestion with an enzymatic solution (0.3% Pectolyase Y-23, 0.3% Driselase, 0.3% Cellulase Onozuka R 10 and 0.1% sodium azide in 10 mM citrate buffer) at 37 °C for 1 h in a humid chamber. After another two washes with citrate buffer, the anthers were separated from surrounding tissue and put onto a slide. One edge of an anther was cut open and the cells within were squeezed out with the tip of a needle. After processing all anthers of one to two buds, cells were mixed into a suspension by stirring with a needle. A small amount of freshly prepared 60% acetic acid was added to the suspension from the side and the whole drop gently homogenized by again stirring with a needle. The slide was placed on a hot plate at 45 °C and cells were spread with the help of a bent needle until almost all liquid evaporated. Around the spread area, a boundary of ice cold fresh fixative solution was created and allowed to enter, followed by a jet of ice cold fixative directly into the center of the area. Then the slide was allowed to air dry in an upright position. Chromosomal spreads were stained with DAPI in a solution of 2 μg/ml in the antifade mounting medium Vectashield and covered with a coverslip. Imaging was done on a Zeiss Axio Imager Z2 Microscope using the ZEN software. Ploidy validation data shown in supplementary data S2, Supplementary Material online.

### Estimation of Nucleotide Diversity

We calculated π (Nei and Li 1979) for diploid and tetraploid live populations with more than five individuals in each (excluding siblings) using piawka (https://github.com/novikovalab/piawka), our implementation of the site weighting strategy introduced in pixy (Korunes and Samuk 2021) compatible with mixed-ploidy datasets. We also calculated π within each tetraploid lineage and between diploid-tetraploid lineages on a per-gene basis with piawka.

### Introgression Analysis

Starting with a VCF containing only biallelic SNPs, we filtered the dataset further using genomics_general-0.4 toolkit (minimum quality = 20, minimum depth = 5, maximum missing data = 0.2, maximum heterozygosity = 0.75, minimum variant count = 5) (https://github.com/simonhmartin/genomics_general). Genealogies were inferred in windows of 50 SNPs along the genome using PHYML (Guindon et al. 2010) and a phasing approach implemented in genomics_general. Previously published (Novikova et al. 2016) samples of *Arabidopsis cebennensis* and *Arabidopsis pedemontana* were used as outgroups. We used Twisst (Martin and Van Belleghem 2017) for topology weighting. GO enrichment analysis using g:Profiler (Kolberg et al. 2023) was performed for genes intersecting the regions with >50% weighting of introgression topologies.

TreeMix (Pickrell and Pritchard 2012) was used with 500 SNPs per block, 4 allowed migration events, and 1,000 bootstrap replicates.

### Demographic Modeling

To generate SFS, we utilized biallelic SNPs and invariant sites. We excluded genic, centromeric, and pericentromeric regions, retaining only SNPs found in intergenic regions of chromosome arms. Individuals of the two main lineages were selected as outlined in supplementary data S1, Supplementary Material online. Using the chosen SNPs, we generated bootstrap replicates by dividing the genome into 10 kb segments. These segments were resampled with replacement until the equivalent size of the total amount of filtered SNPs in the genome. We computed site frequency spectrum (SFS) for each population of the 200 bootstrapped replicates and a joint site frequency spectrum for each pseudo-observation using the Python package dadi (Gutenkunst et al. 2009). The SFS spectra generated by dadi were used as input into fastsimcoal2 (fsc27) (Excoffier et al. 2021). We first assessed a simple divergence model for both lineages through 50 fastsimcoal2 runs, then compared with a model allowing migration from the diploid into tetraploid lineage. Each fastsimcoal2 run was allowed 40 optimization cycles and 100,000 iterations. Based on likelihood and AIC criteria we continued with the divergence mode with migration for the confidence interval calculation. For each of the 200 bootstrap replicates we initiated 50 fastsimcoal2 runs. The resulting parameter estimates from the 200 pseudo-observations were used to calculate the 95% confidence intervals in R.

## Supplementary Material

msaf153_Supplementary_Data

## Data Availability

The whole-genome re-sequencing short reads data for the samples which was generated in this study were submitted to the ENA database under study numbers PRJEB67879 and PRJEB60410. The list of all the samples used in the study with individual ENA numbers is in supplementary data S1, Supplementary Material online. The images of the herbarium vouchers can be found online using an example link https://plant.depo.msu.ru/open/public/item/MW0079552 and replacing MW0079552 with the individual number from supplementary data S1, Supplementary Material online column “accession”. Both raw stLFR data and resulting assemblies are also accessible in the ENA database, with individual ENA numbers in supplementary data S5, Supplementary Material online. supplementary data S1 to S5, Supplementary Material online and the scripts used in the analysis are available at https://github.com/novikovalab/Siberian_Alyrata.
