# Supplementary material for: Multiple Autopolyploid Arabidopsis lyrata Populations Stabilized by Long-Range Adaptive Introgression Across Eurasia
Source: Mol Biol Evol. 2025 Jul 24;42(8):msaf153. doi: 10.1093/molbev/msaf153 (PMC12342985; doi:10.1093/molbev/msaf153)
Supplement: msaf153_Supplementary_Data [file msaf153_supplementary_data.zip › SupplementaryData2.pdf]

Experimental validation of ploidy in Siberian A. lyrata populations

Chromosomal spreads

WS1.1-2 somatic cell 2n=32
WS1.1-2 4n A. lyrata metaphases (>8 bivalents)
TE4.3-1 somatic cell 2n=16
TE8.3-1 somatic cell 2n=16
TE8.8-2 somatic cell 2n=16
PU6.6-1 somatic cell 2n=32
PU7.2-2 4n A. lyrata metaphases (>8 bivalents)
AL1.5-1 4n A. lyrata metaphases (>8 bivalents)
AL1.5-3 somatic cell 2n=32
NT2.4-1 somatic cell 2n=16
NT10.1-1 somatic cell 2n=16
NT10.1-1 2n A. lyrata metaphases (<=8 bivalents)

WS1.1-2 somatic cell 2n=32

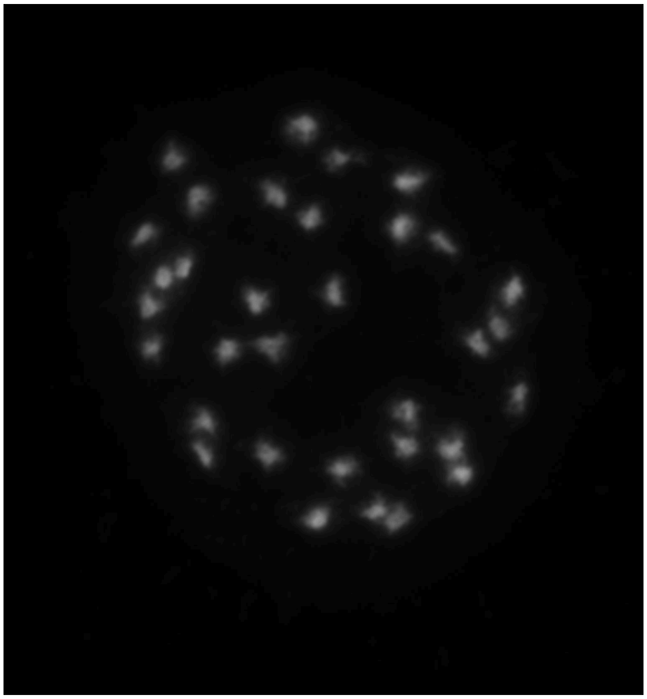

WS 1.1-2anthers1-01

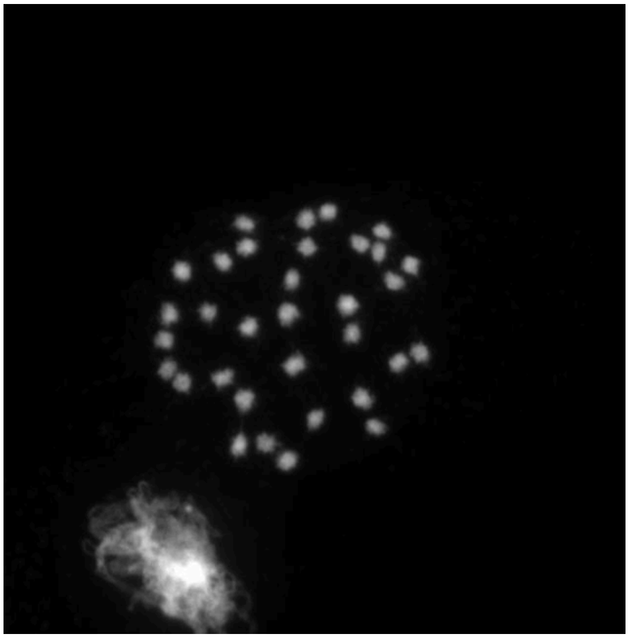

WS1.1-2anth1-19_32Chrs

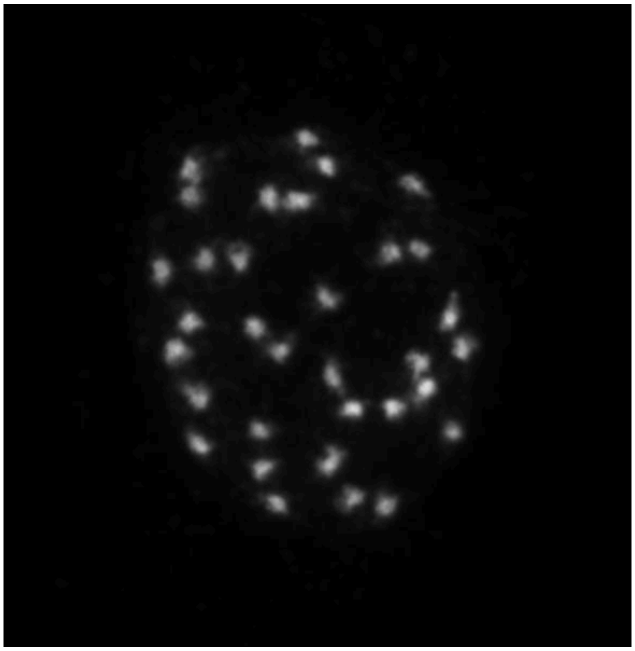

WS1.1-2anth1-25_32Chrs

Meiotic behavior

WS1.1-2 4n A. lyrata metaphases (>8 bivalents)

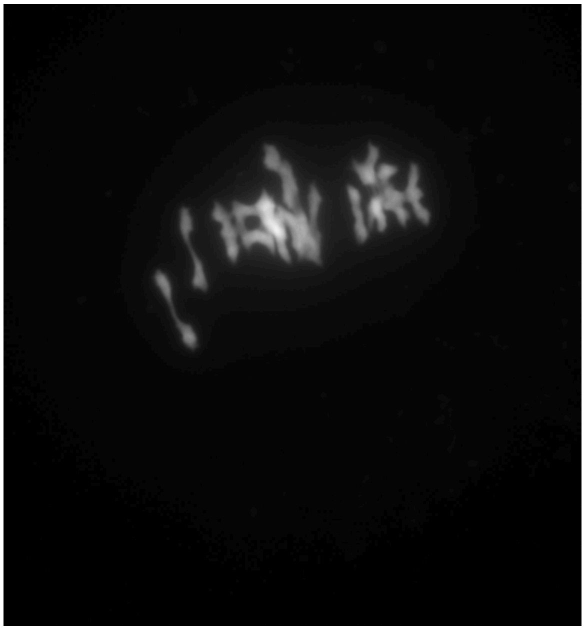

WS 1.1-2anthers1-13metaphase

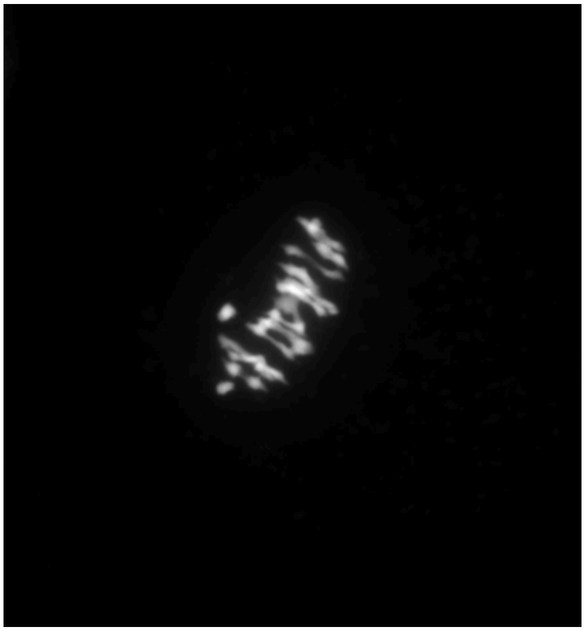

WS 1.1-2anthers1-16metaphase

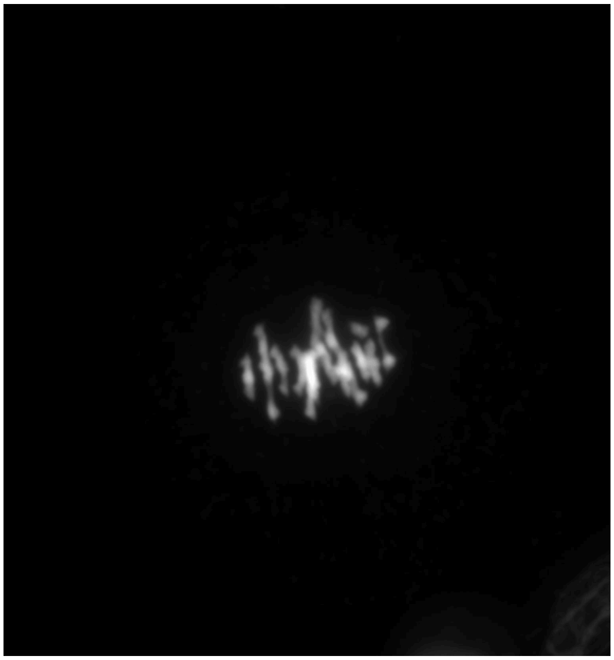

WS 1.1-2anthers1-17metaphase

TE4.3-1 somatic cell 2n=16

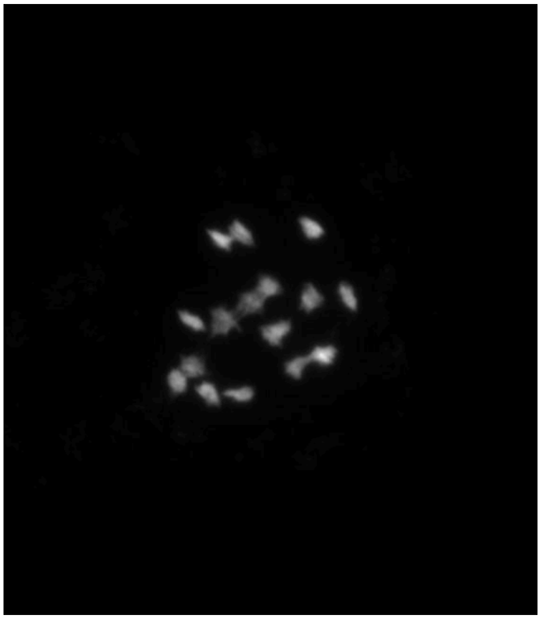

TE 4.3-1 anthers1-05, 16Chroms zoom

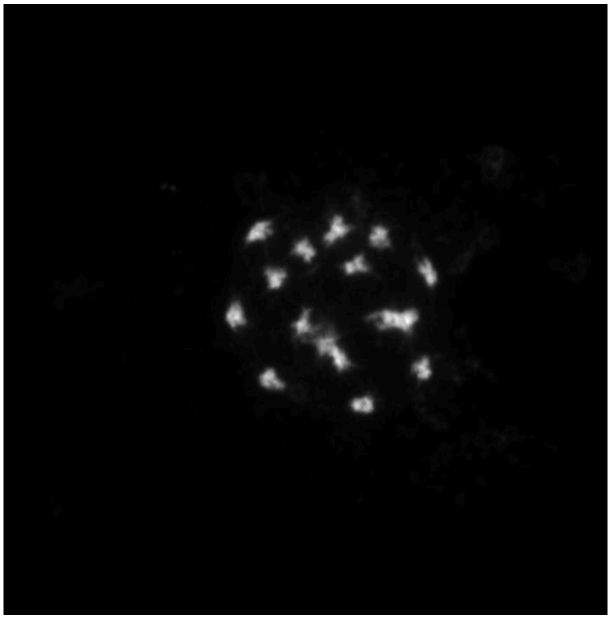

TE 4.3-1 anthers1-06, 16Chroms zoom

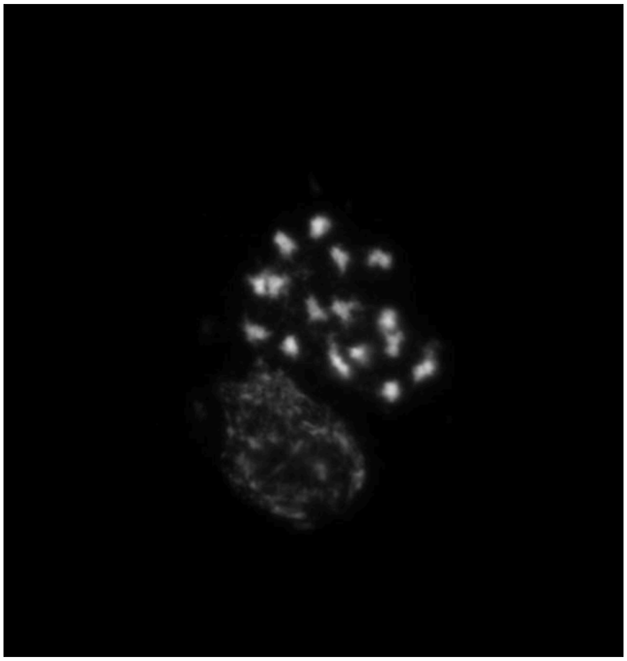

TE 4.3-1 anthers1-08, 16Chroms zoom

TE8.3-1 somatic cell 2n=16

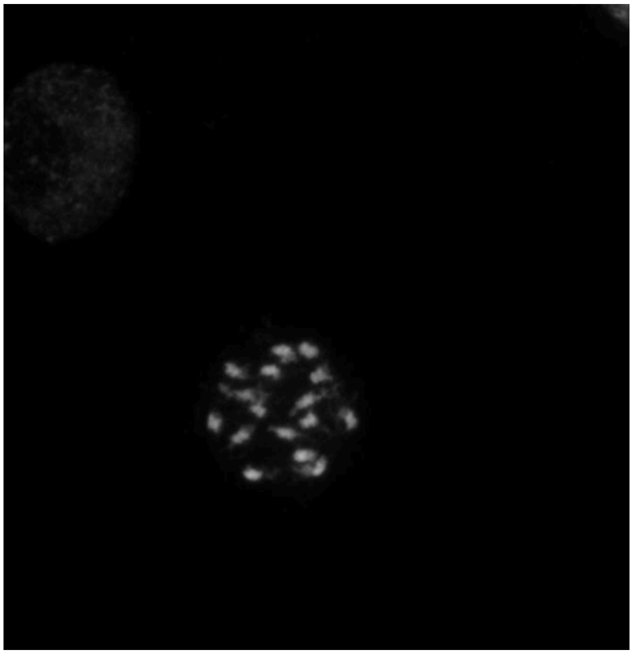

TE 8.3-1 anthers2-09, 16Chroms

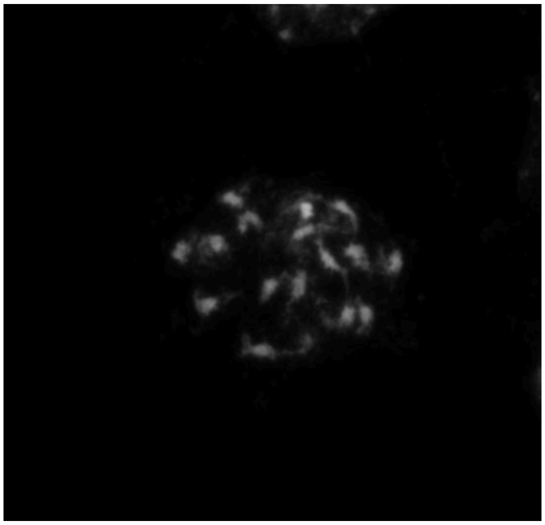

TE 8.3-1 anthers2-05, 16Chroms

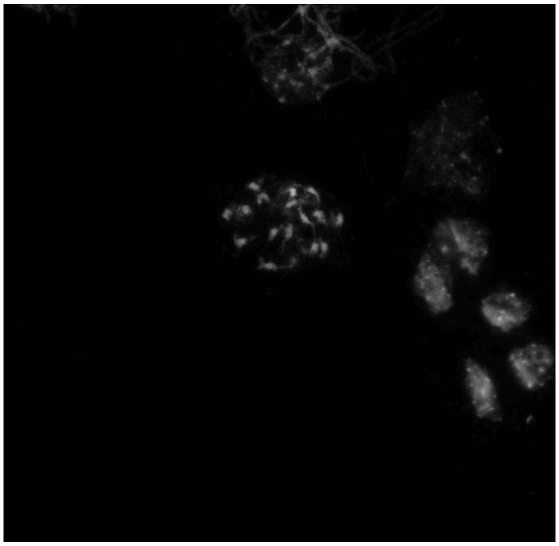

TE 8.3-1 anthers2-05, 16Chroms

TE8.8-2 somatic cell 2n=16

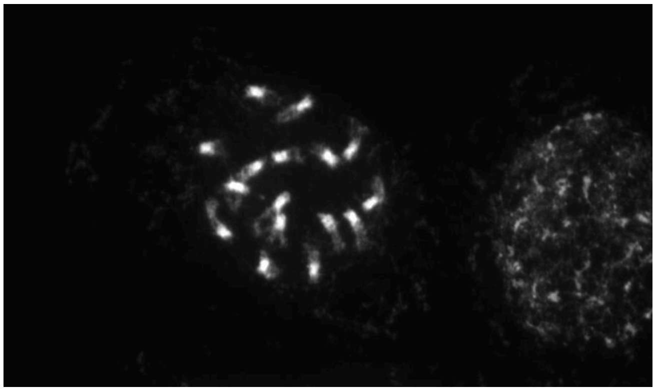

TE 8.8-2anth1-01_16Chrs

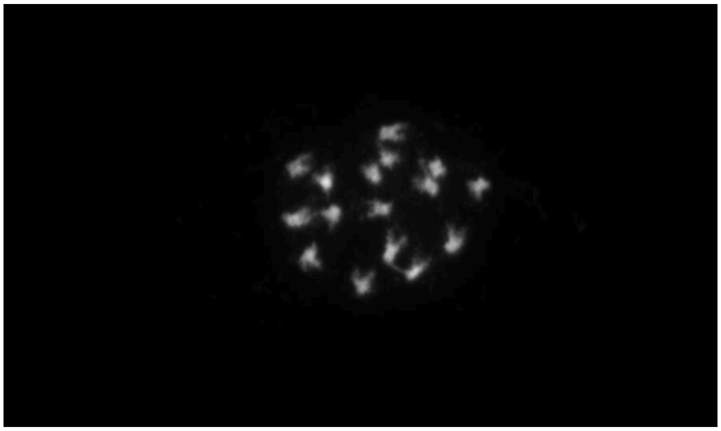

TE 8.8-2anth1-14_16Chrs

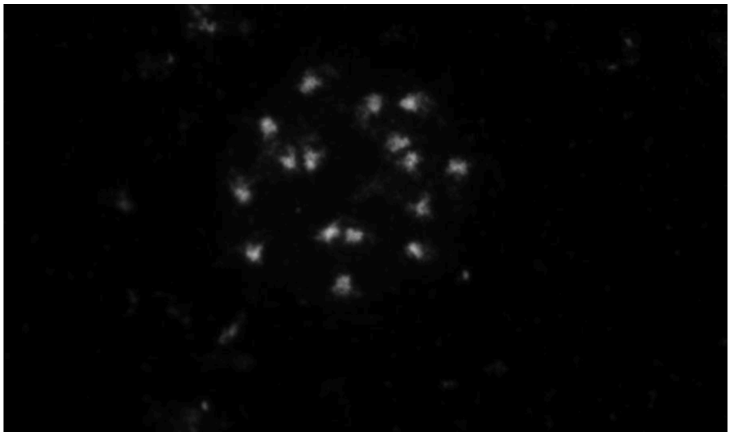

TE 8.8-2anth2-01_16Chrs

PU6.6-1 somatic cell 2n=32

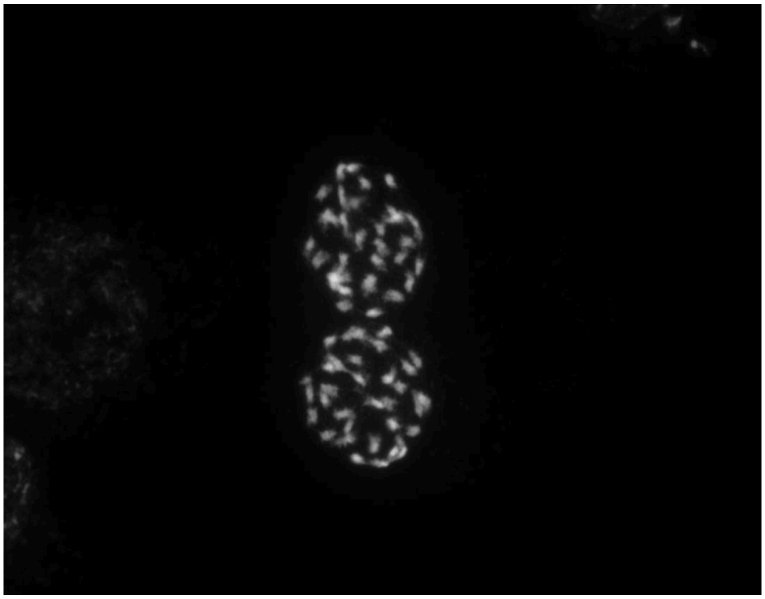

NewPU6.6-1(1)-03_2x 32 somatic

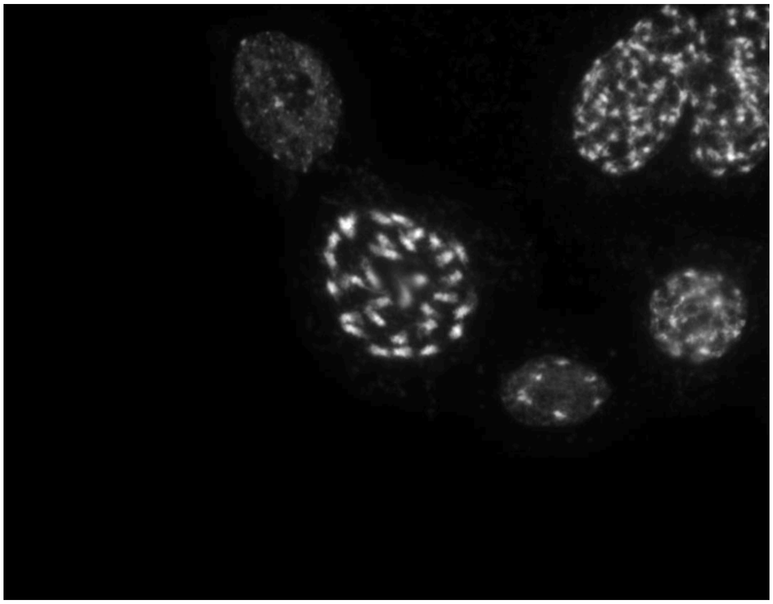

PU6.6-1(1)-03_Somatic

PU7.2-2 4n A. lyrata metaphases (>8 bivalents)

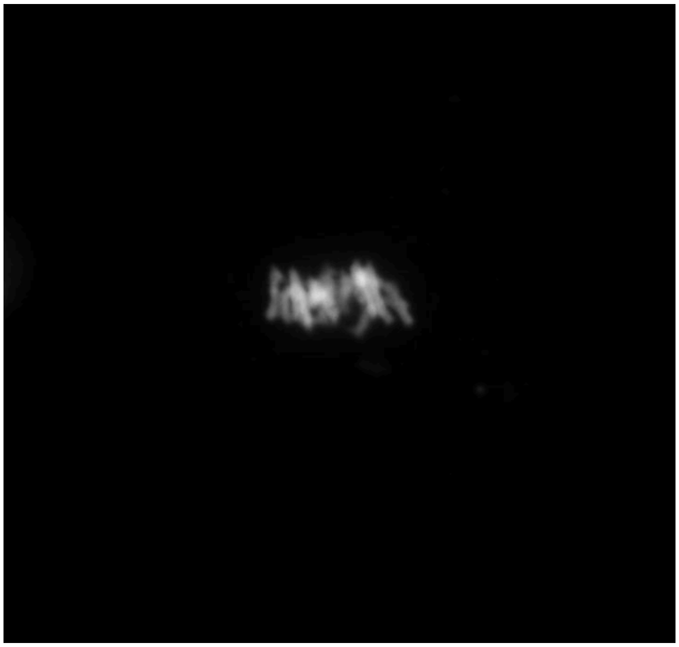

PU 7.2-2_2-19 Metaphase I

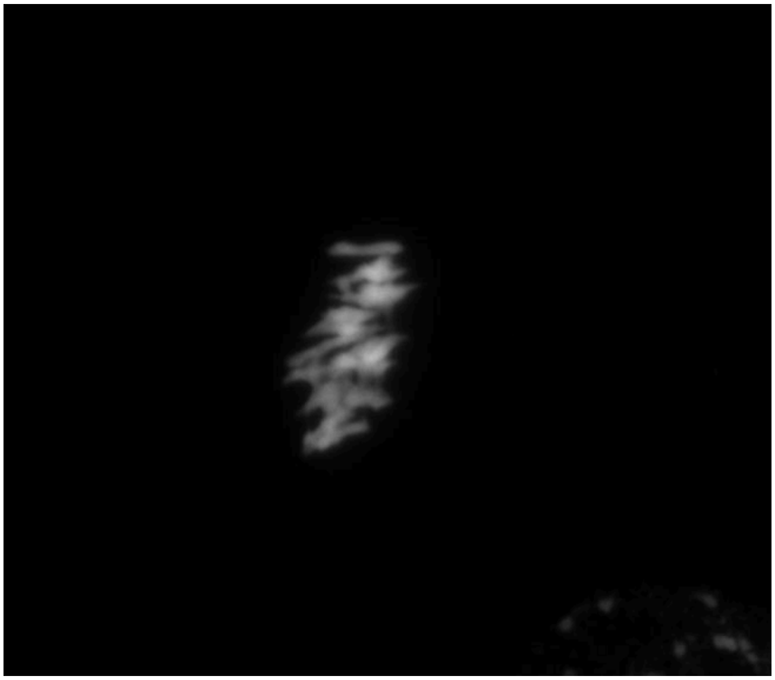

PU 7.2-2_2-32 Metaphase I

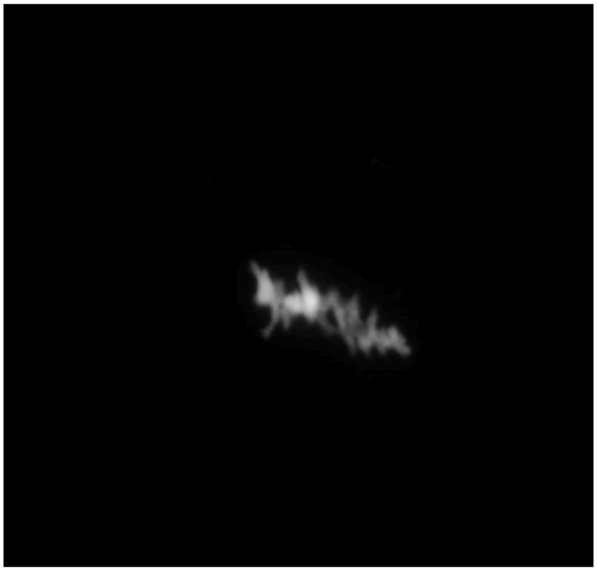

PU 7.2-2_2-20 Metaphase I

AL1.5-1 4n A. lyrata metaphases (>8 bivalents)

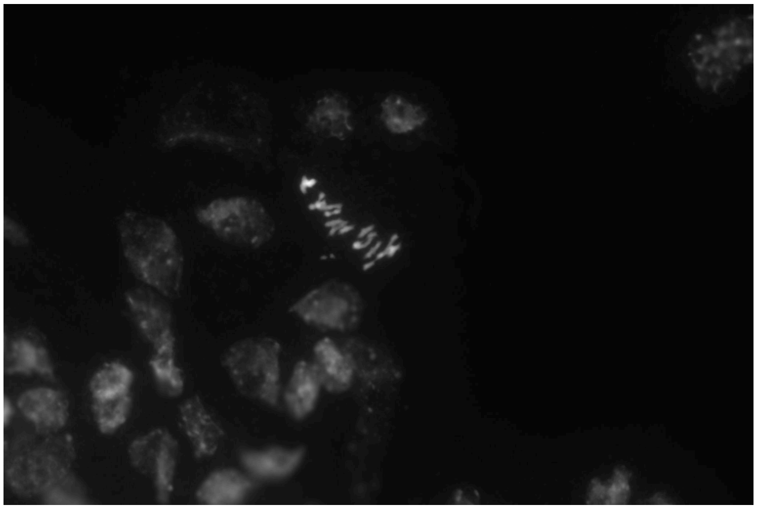

al1-5.1-05_Metaphase I

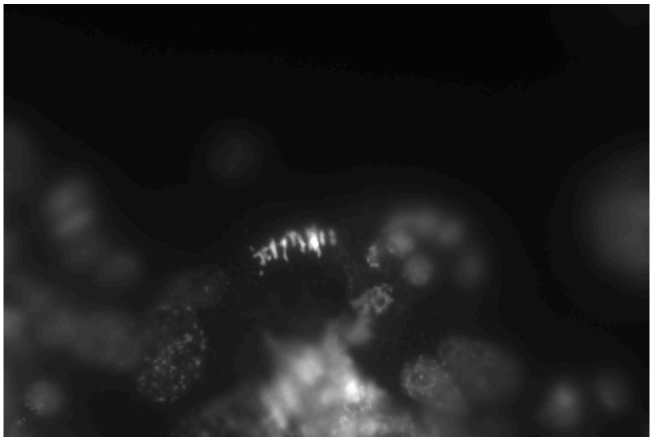

al1-5.1-01-2metaphase I

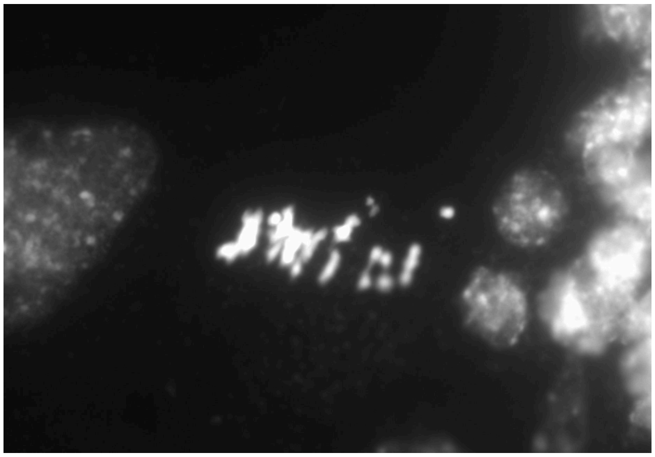

al1-5.1-09metaphase I

AL1.5-3 somatic cell 2n=32

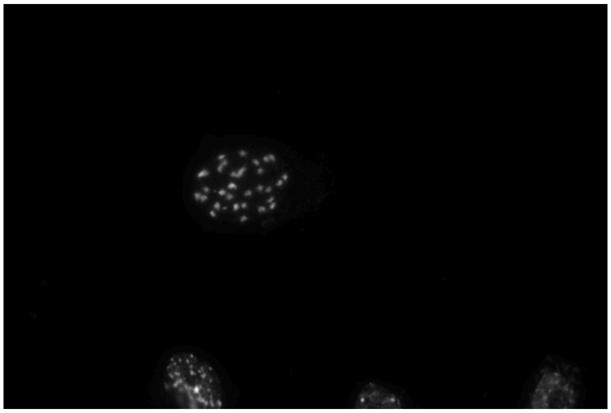

AI1-5.3-01

NT2.4-1 somatic cell 2n=16

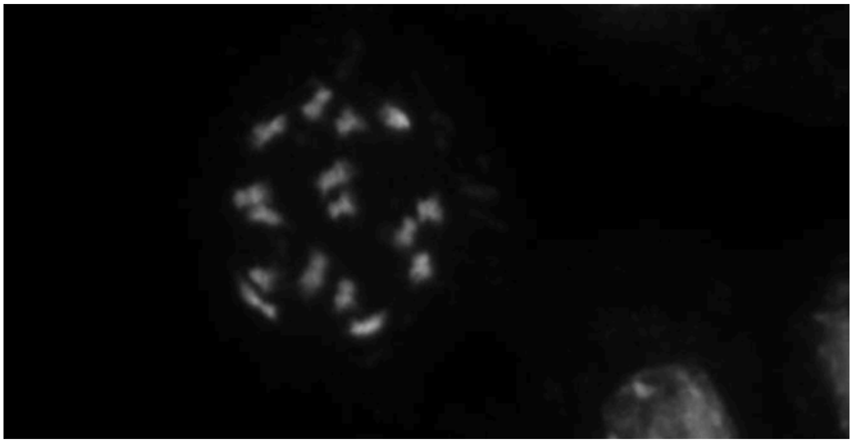

NT2.4-1_1-05_16 Chrs

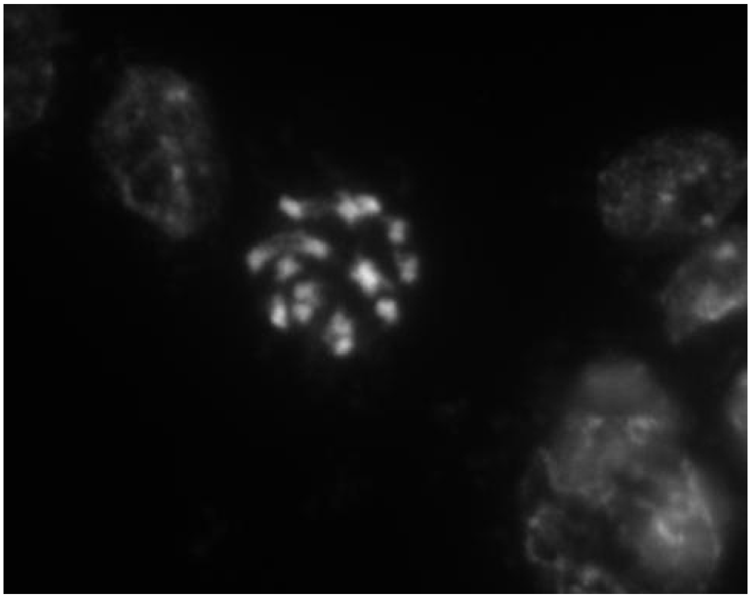

NT2.4-1_3-17_15Chrs

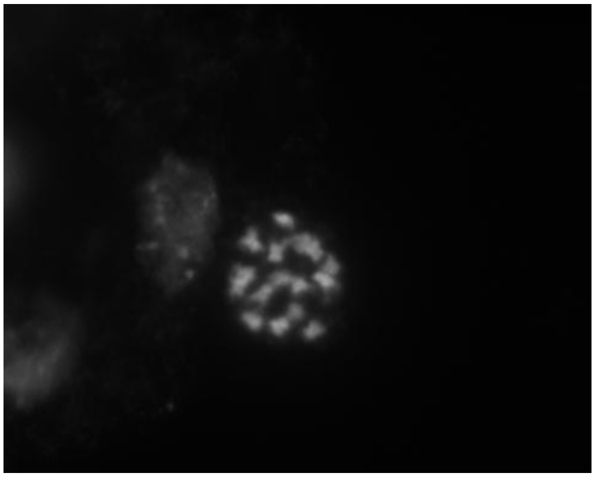

NT2.4-1_3-28_16Chrs

NT10.1-1 somatic cell 2n=16

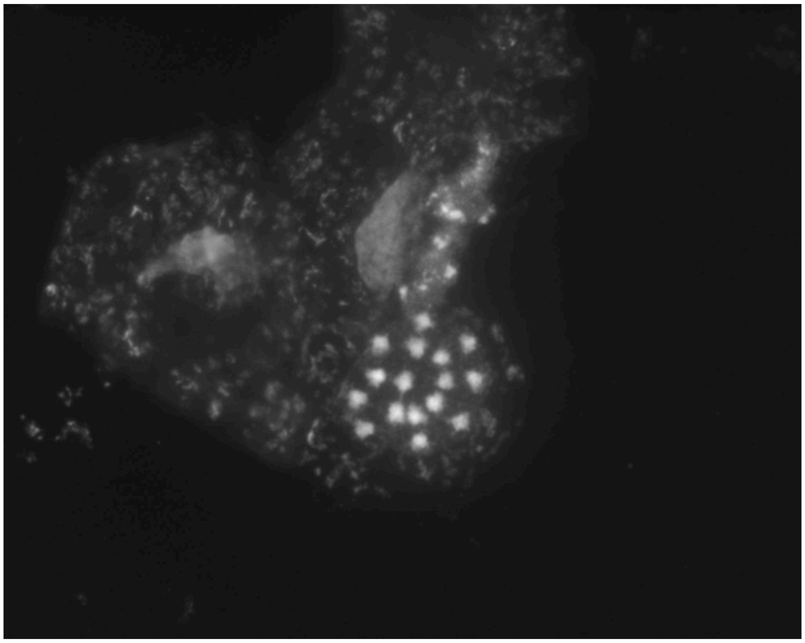

NT10.1-1-1-01_16Chrs

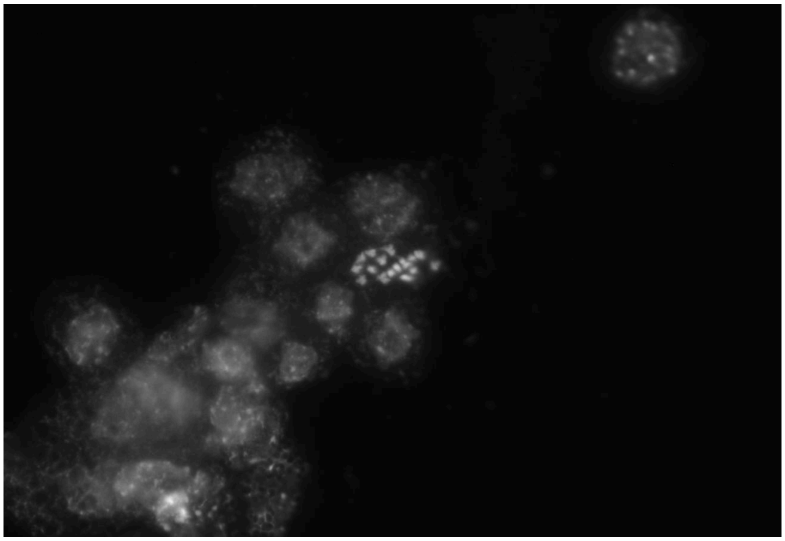

NT10.1-1-1-03_16Chrs

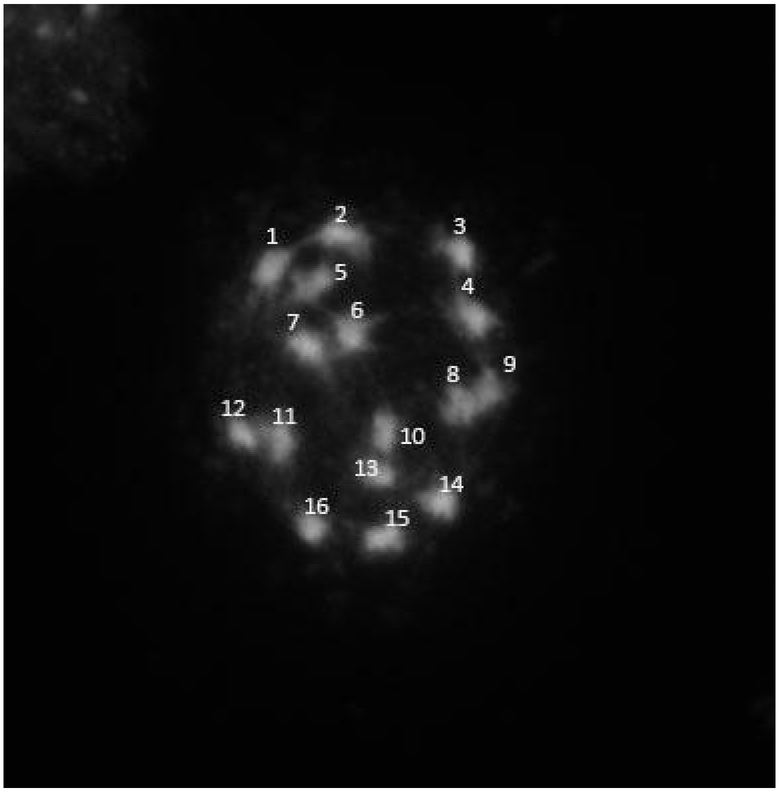

NT10.1-1-2-03_16ChrsPScounted

NT10.1-1 2n A. lyrata metaphases (<=8 bivalents)

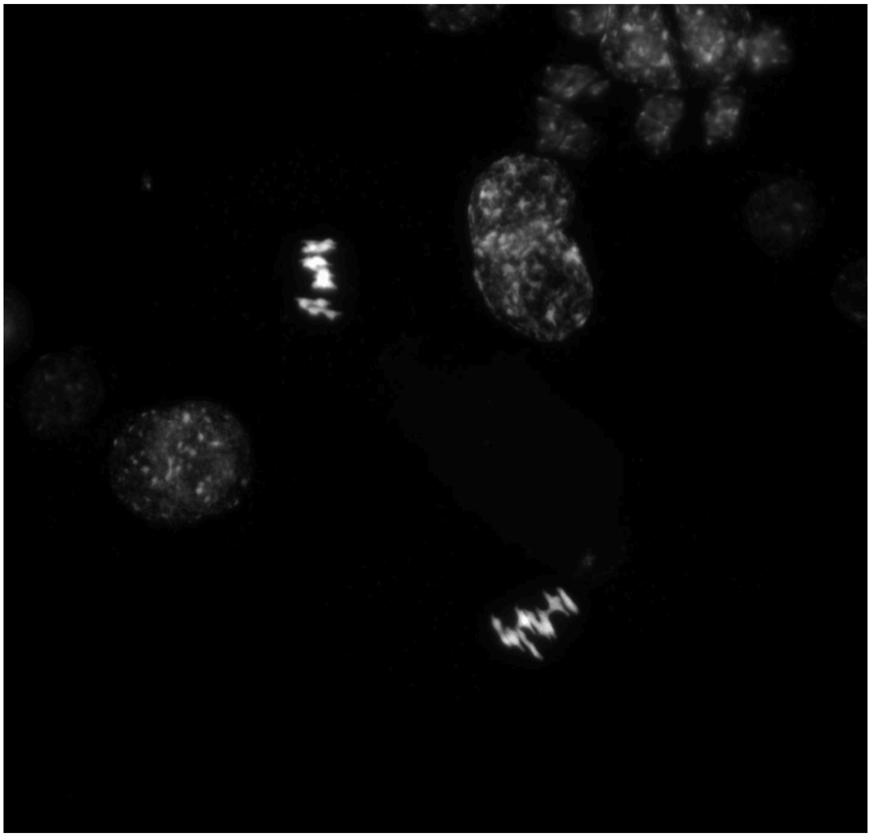

NT10.1-1-3-12metaph

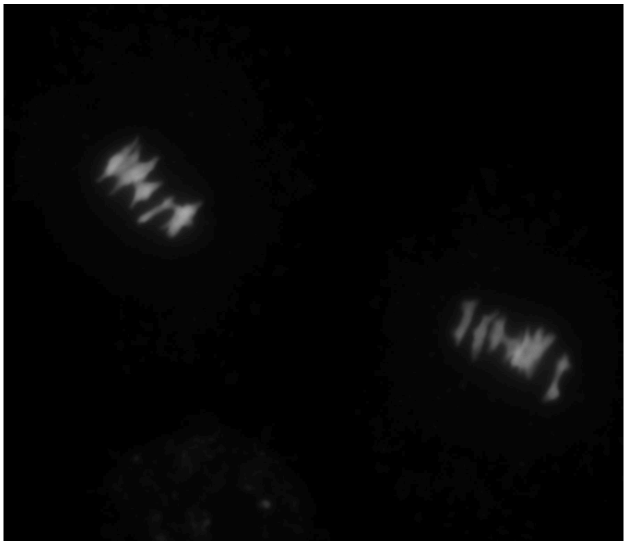

NT10.1-1-3-17metaph

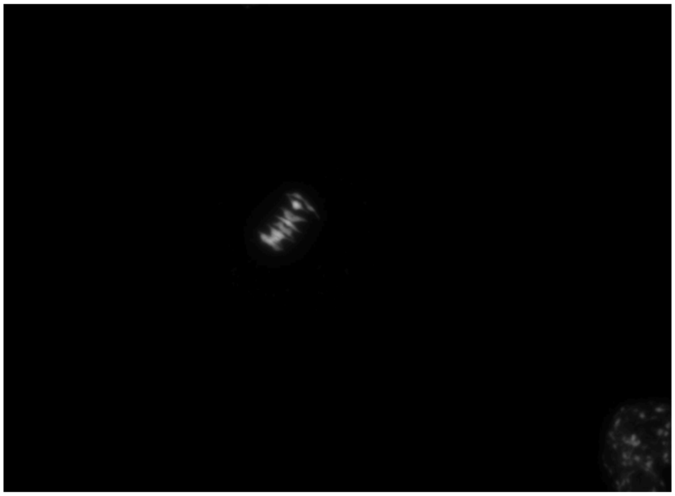

NT10.1-1-3-27metaph
