# Supplementary material for: Multiple Autopolyploid Arabidopsis lyrata Populations Stabilized by Long-Range Adaptive Introgression Across Eurasia
Source: Mol Biol Evol. 2025 Jul 24;42(8):msaf153. doi: 10.1093/molbev/msaf153 (PMC12342985; doi:10.1093/molbev/msaf153)
Supplement: msaf153_Supplementary_Data [file msaf153_supplementary_data.zip › SupplementaryMaterials.pdf]

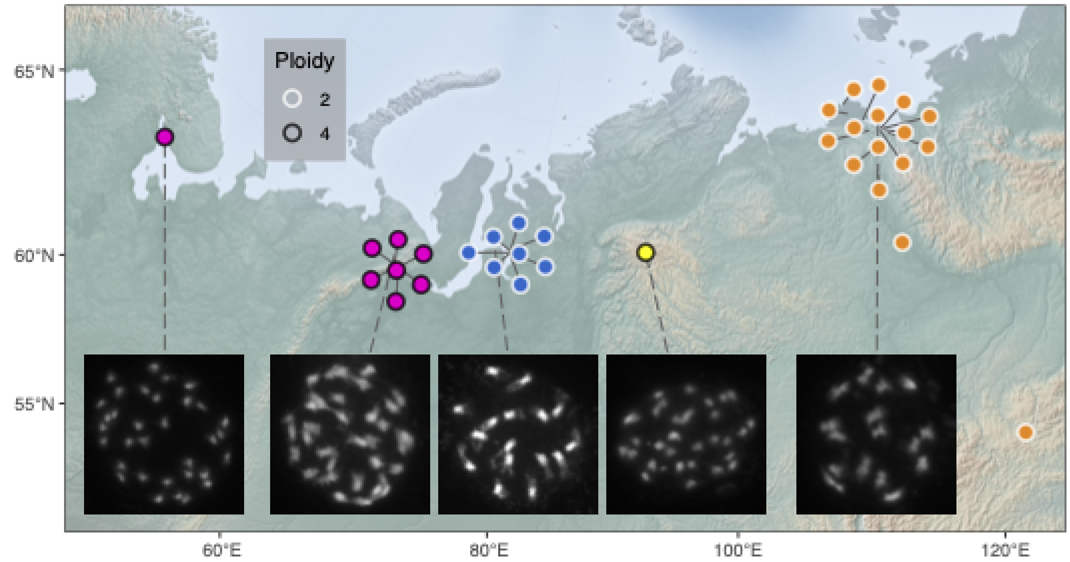

Supplementary Figure 1. Geographical map of populations with collected seeds. Seeds were grown in the green house and karyotyped to confirm the ploidy inference of each lineage, colors corresponding to those in Figure 2.

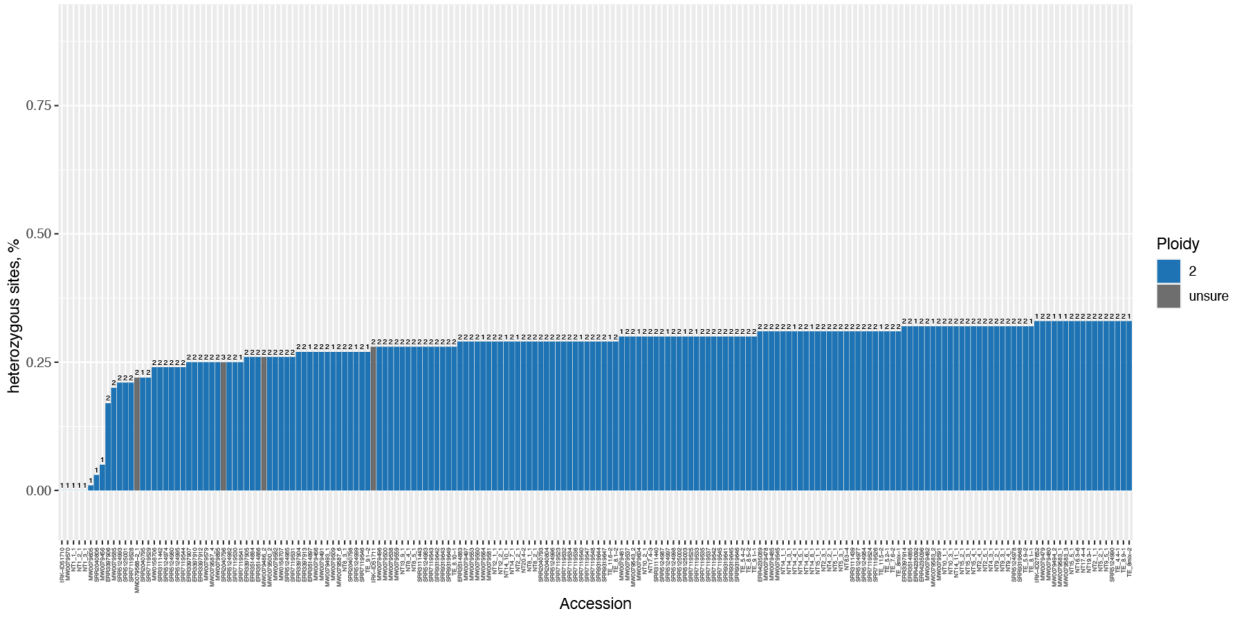

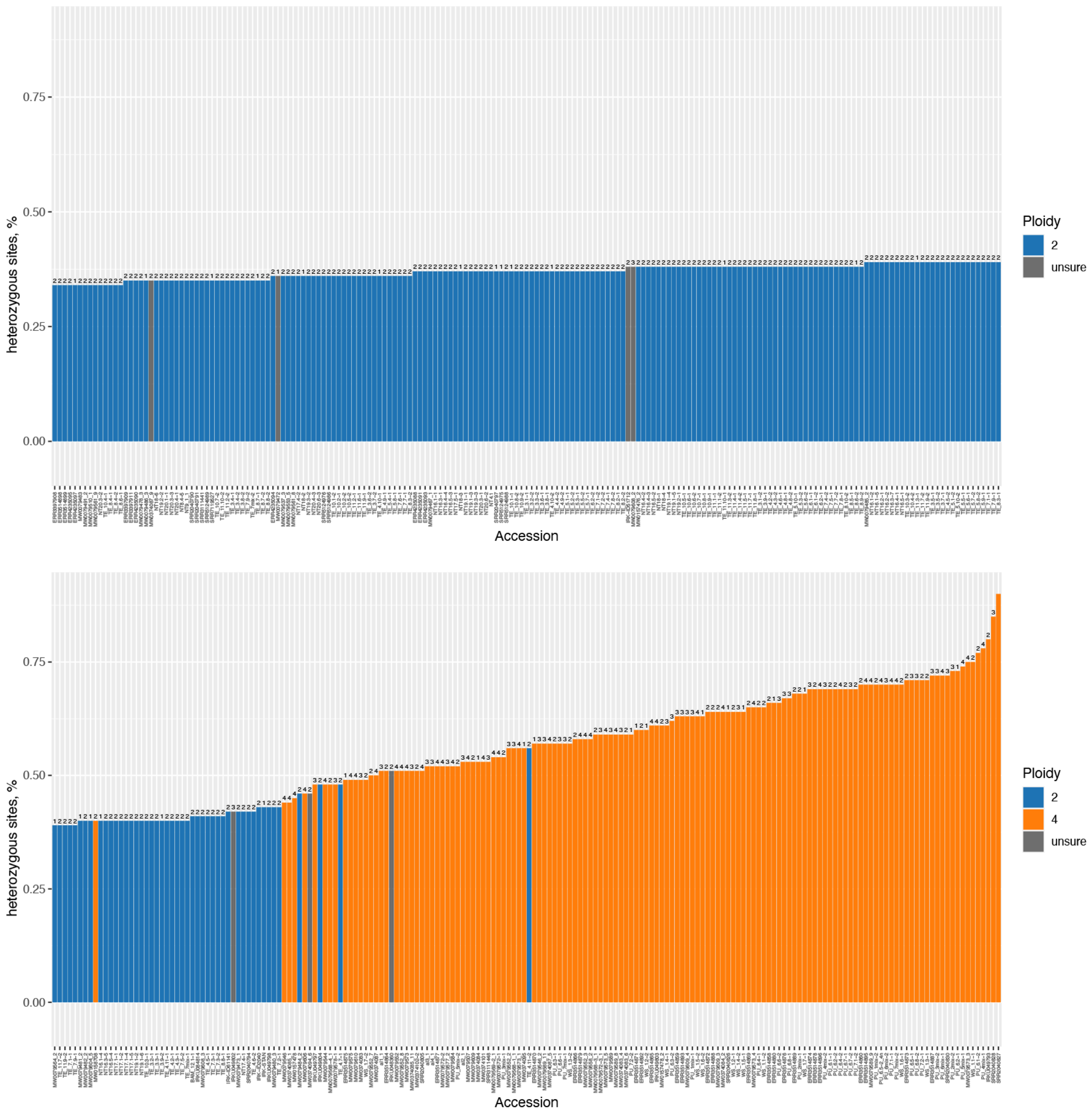

Supplementary Figure 2. Heterozygosity and ploidy estimates. Bar height corresponds to percentage of heterozygous sites. Bar color is ploidy level inferred by nQuire (orange = tetraploid, blue = diploid, grey = unsure). The number of different *S*-alleles is indicated on top of each bar. Overall, tetraploid samples have higher heterozygosity than diploids. Data used to plot this figure are in Supplementary Data 1.

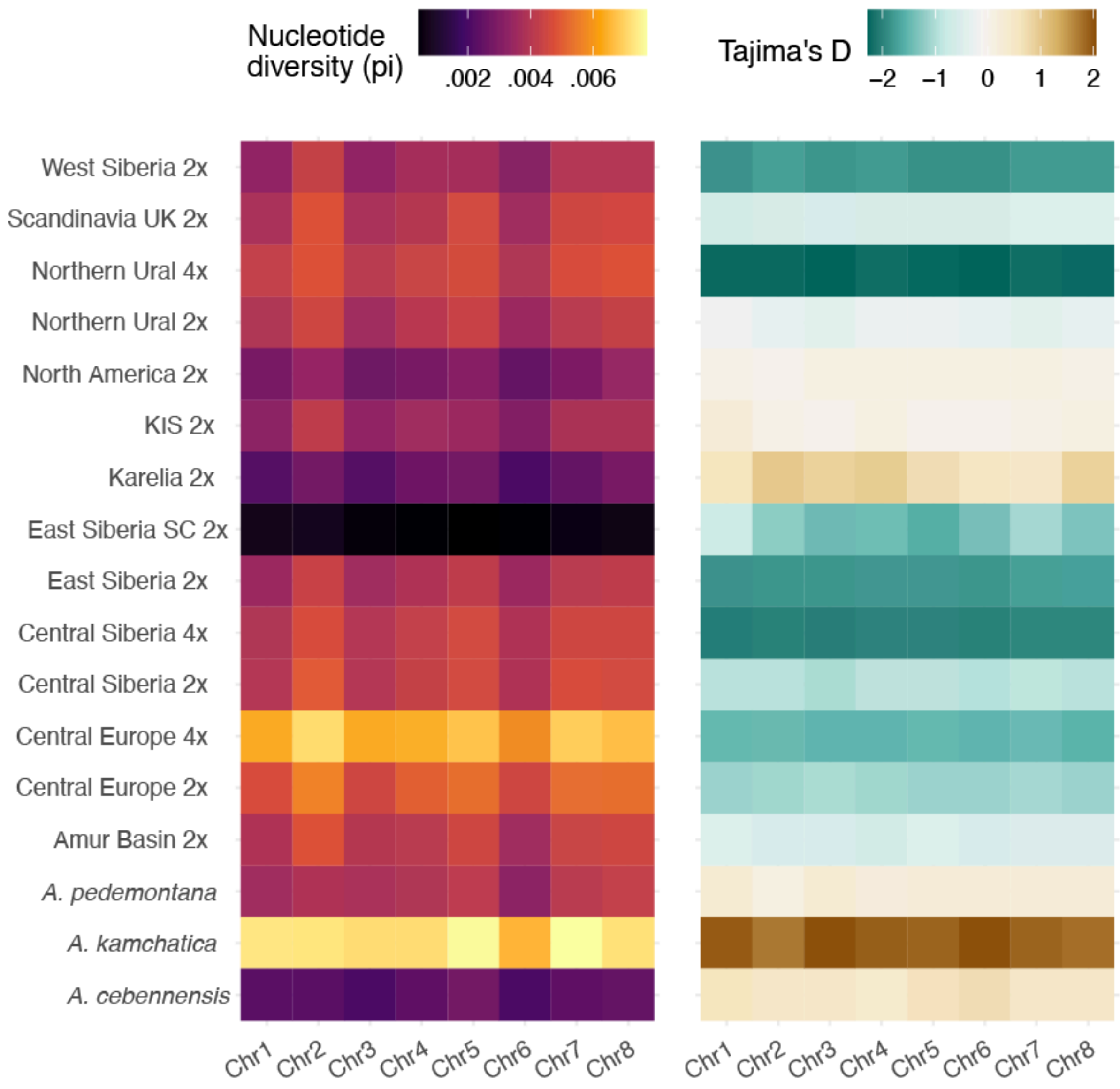

Supplementary Figure 3. Nucleotide diversity and Tajima's D per scaffold for A. lyrata lineages and outgroups. Nucleotide diversity (left heatmap) is lowest in self-compatible diploids from East Siberia (black bar), and highest in allotetraploid *A. kamchatica* (light yellow). Tajima's D (right column) is lowest in Northern Ural tetraploids (dark turquoise bar) and highest in *A. kamchatica* (brown bar).

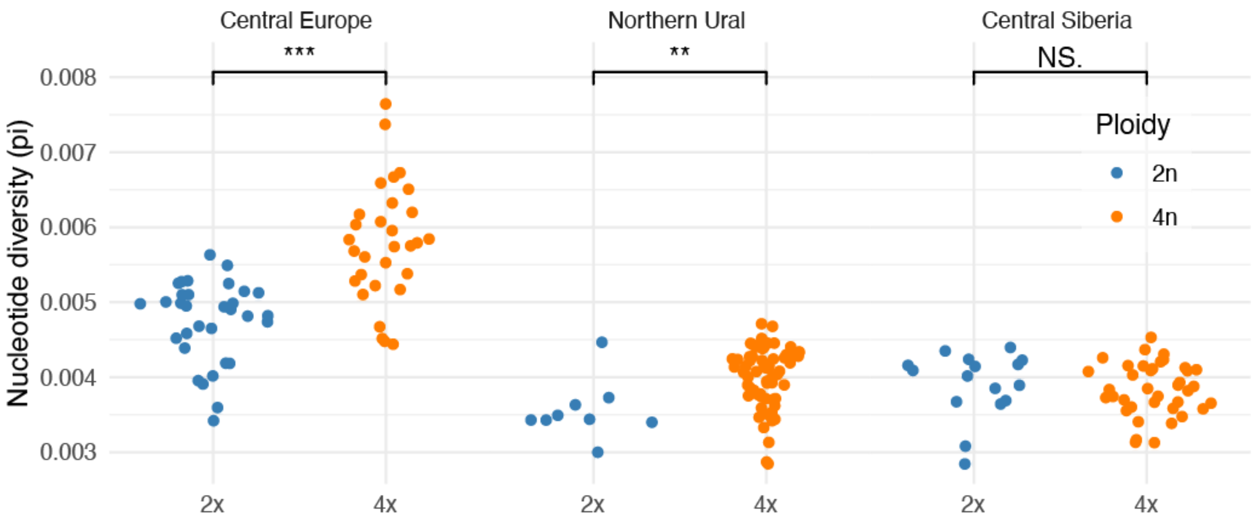

Supplementary Figure 4. Genome-wide nucleotide diversity (pi) calculated per individual from diploid and tetraploid lineages. Tetraploids in orange, diploids in blue. Significant differences between ploidy levels calculated via T-test.

Supplementary Figure 5 (as separate pdf).

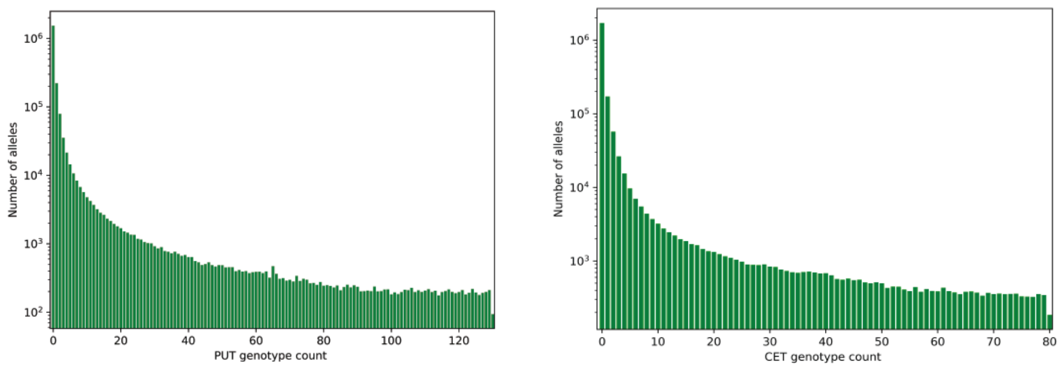

Supplementary Figure 6. Site frequency spectra of Northern Ural and Central Siberia tetraploids, with absence of peak at the intermediate frequencies, which would be expected for allopolyploids due to fixed heterozygosity from two divergent subgenomes.

a

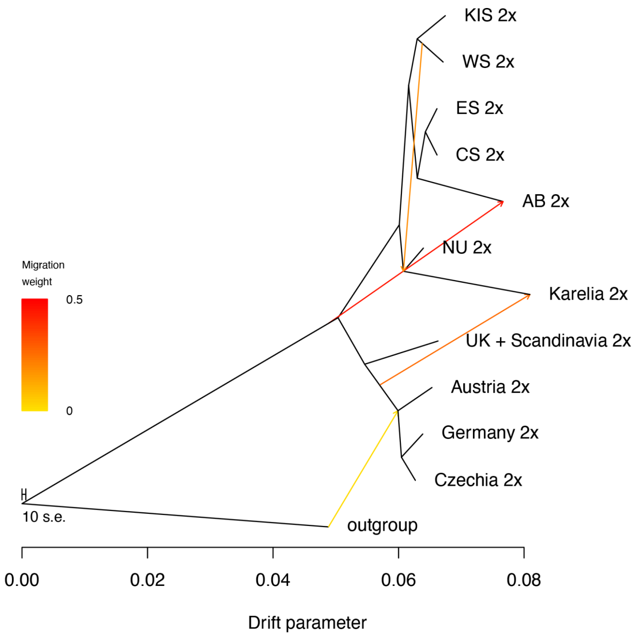

KIS 2x
WS 2x
ES 2x
CS 2x
AB 2x
NU 2x
Karelia 2x
UK + Scandinavia 2x
Austria 2x
Germany 2x
Czechia 2x
outgroup
Migration weight
0.5
0
10 s.e.
0.00
0.02
0.04
0.06
0.08
Drift parameter

b

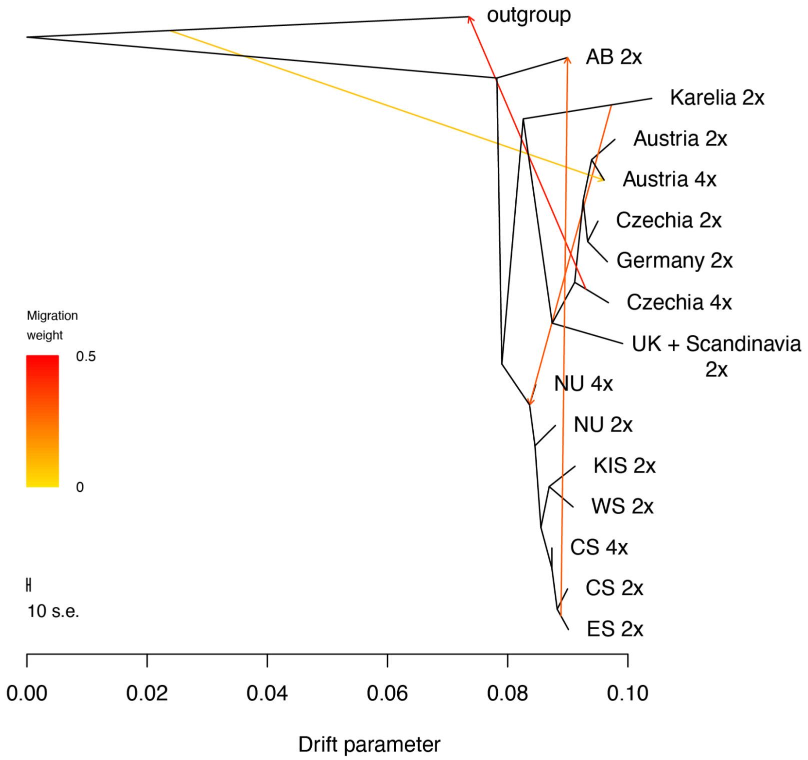
outgroup
AB 2x
Karelia 2x
Austria 2x
Austria 4x
Czechia 2x
Germany 2x
Czechia 4x
UK + Scandinavia 2x
NU 4x
NU 2x
KIS 2x
WS 2x
CS 4x
CS 2x
ES 2x
Migration weight
0.5
0
10 s.e.
0.00
0.02
0.04
0.06
0.08
0.10
Drift parameter

c

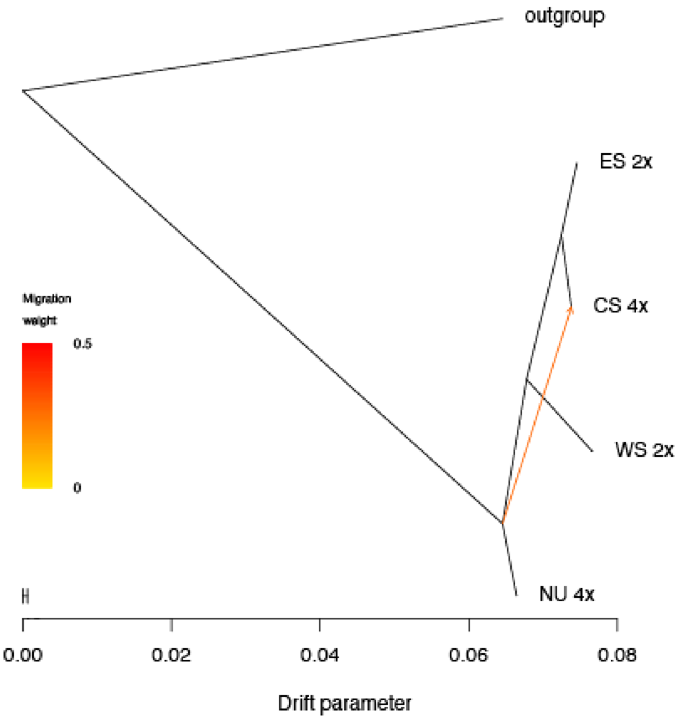
outgroup
ES 2x
CS 4x
WS 2x
NU 4x
Migration weight
0.5
0
0.00
0.02
0.04
0.06
0.08
Drift parameter

Supplementary Figure 7. Maximum likelihood TreeMix models including diploids only (top), with tetraploids (middle), and diploids and tetraploids from the same lineages as ASTRAL tree in Figure 2 (bottom).. NU - Northern Ural, WS - Western Siberia, CS - Central Siberia, ES - Eastern Siberia, KIS - Kuz'kin Island, AB - Amur Basin.

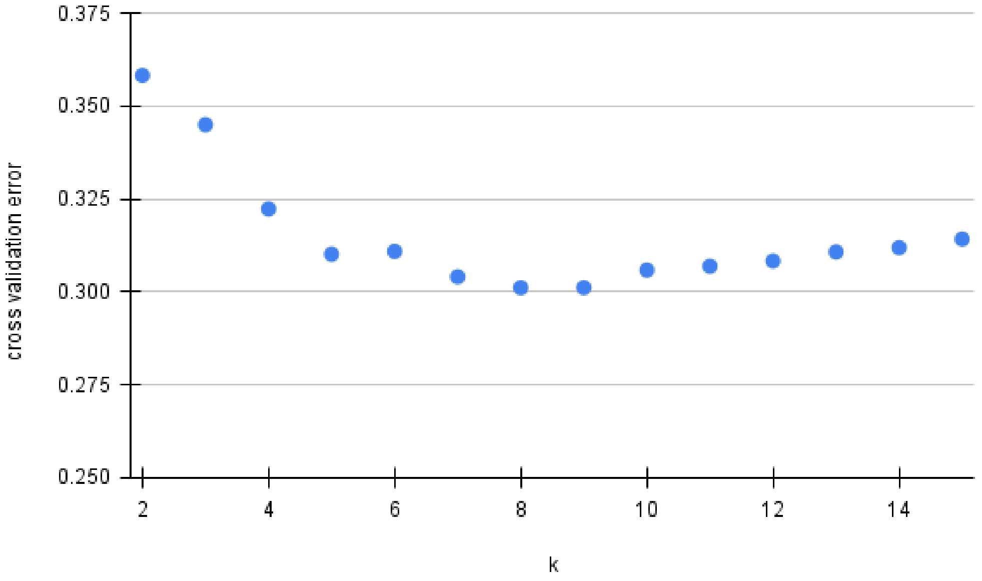

Supplementary Figure 8. Cross-validation error plot to choose k for admixture. K=8 was chosen as an optimal number of clusters for Figure 2c

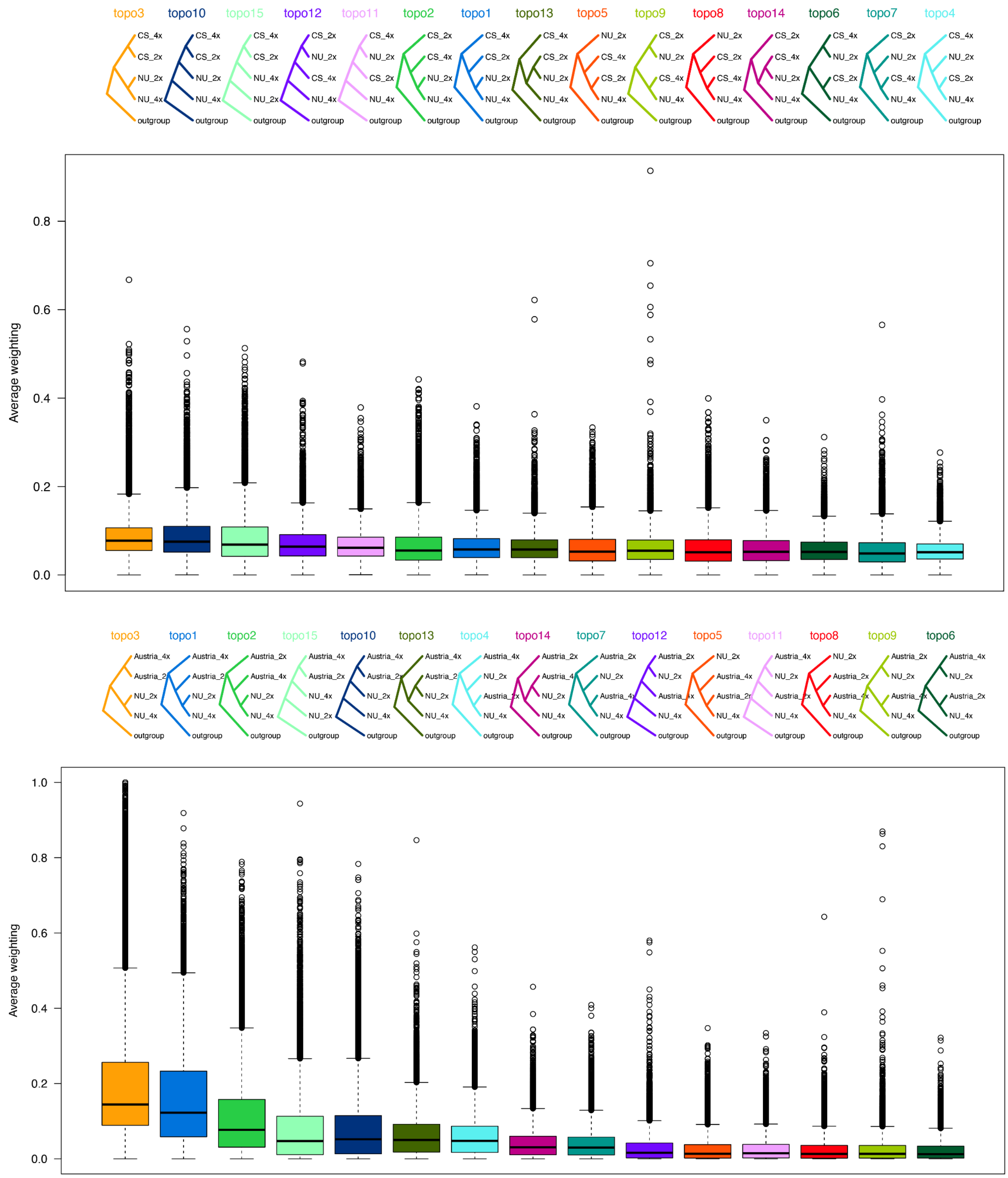

Supplementary Figure 9. Full topology weighting results across all possible topologies. Upper plot, topology weighting for Northern Ural and Central Siberian lineages (introgression topologies are 7,8,9). Lower plot, topology weighting for Northern Ural and Central Europe lineages (introgression topologies are 7,8,9).

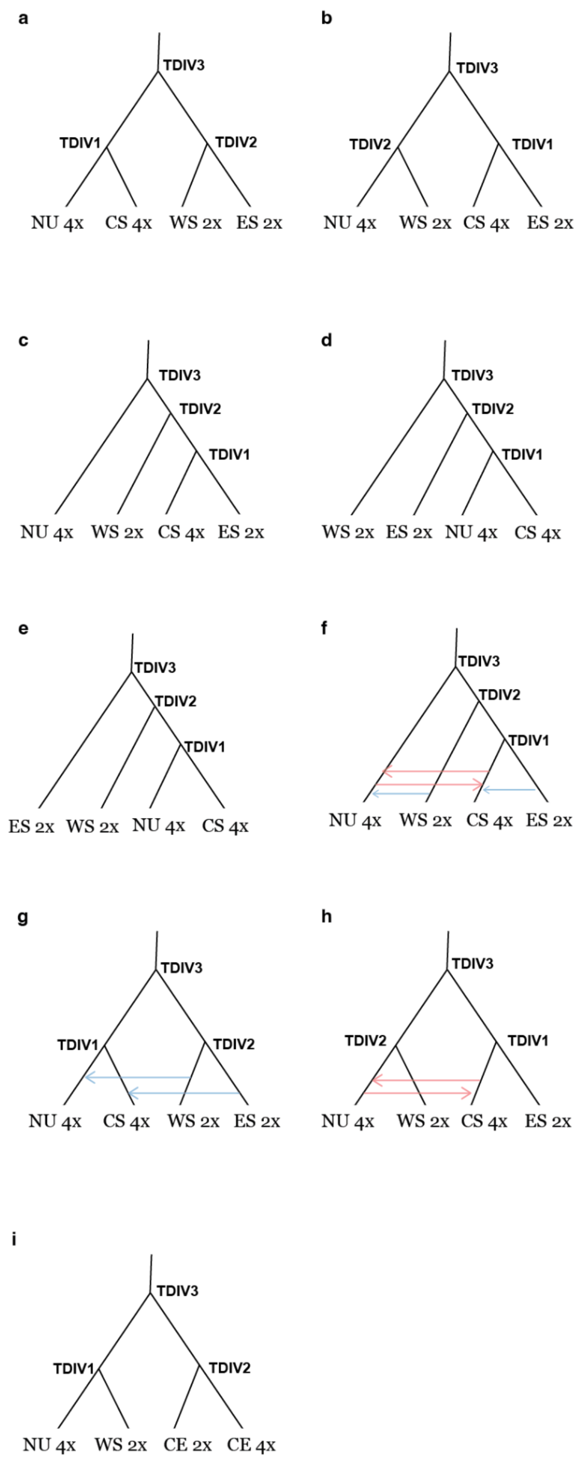

Supplementary Figure 10: Demographic models tested. Models a,d,e test a single origin of tetraploids. Models b and c test independent origins. Models f,g,h include migration between tetraploids (red arrows, in two-origin scenarios) and local migration between ploidy levels (blue arrows).

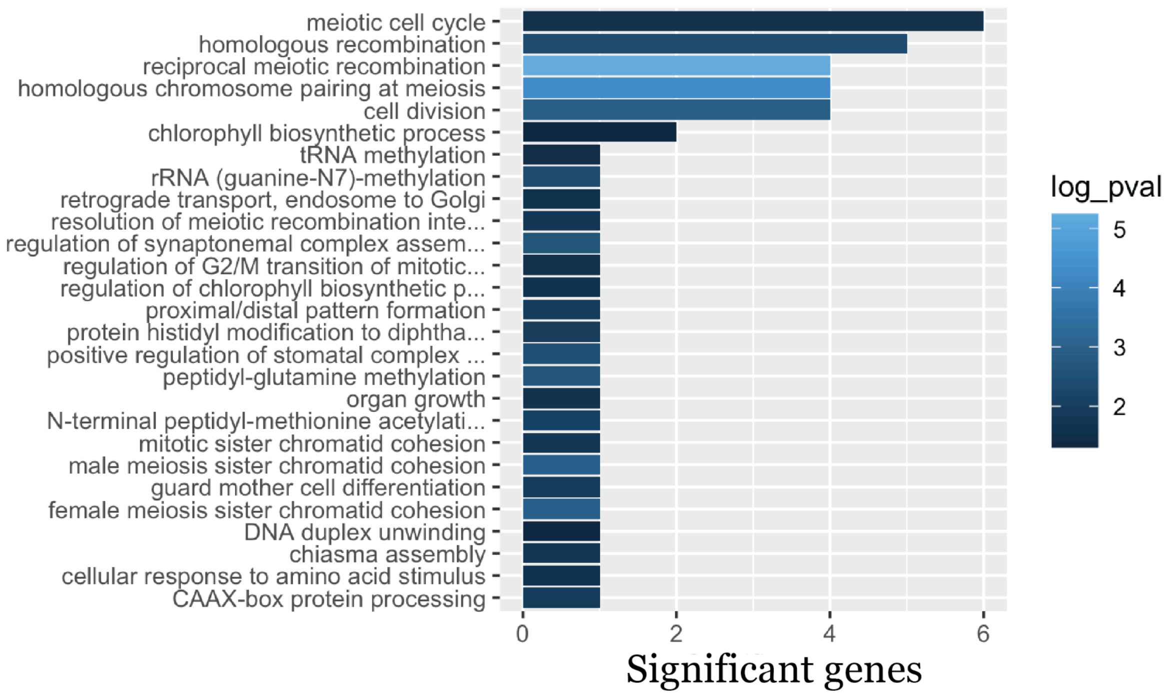

Supplementary Figure 11: Gene ontology enrichment of genes within introgression windows. Gene ontology categories listed on Y axis. Number of significant genes within each category on the X axis. Color of bars indicates -log p-value.

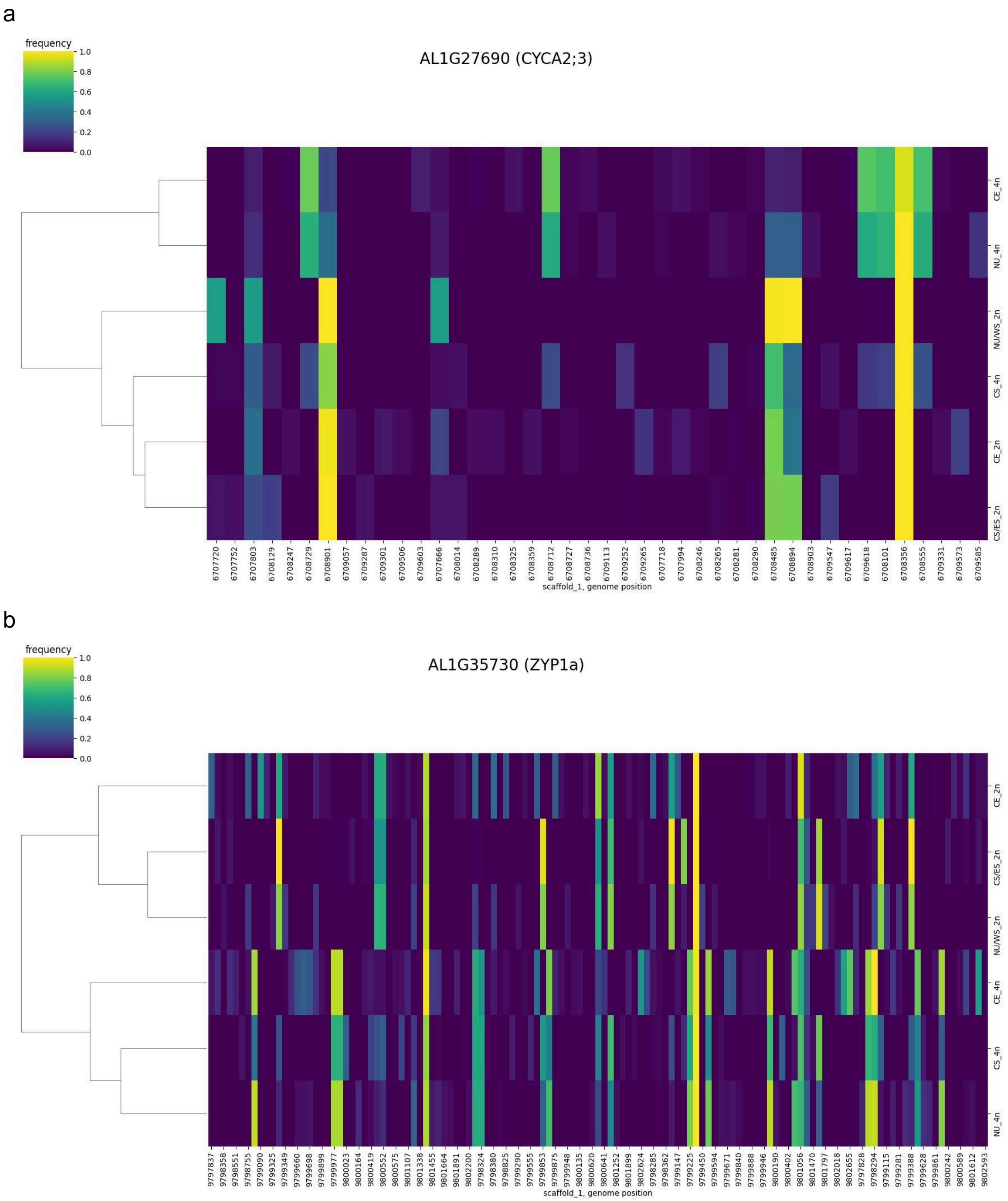
a
frequency
AL1G27690 (CYCA2;3)
CE_4n
NU_4n
NU/WS_2n
CS_4n
CE_2n
CS/ES_2n
scaffold_1, genome position
b
frequency
AL1G35730 (ZYP1a)
CE_2n
CS/ES_2n
NU/WS_2n
CE_4n
CS_4n
NU_4n
scaffold_1, genome position

c

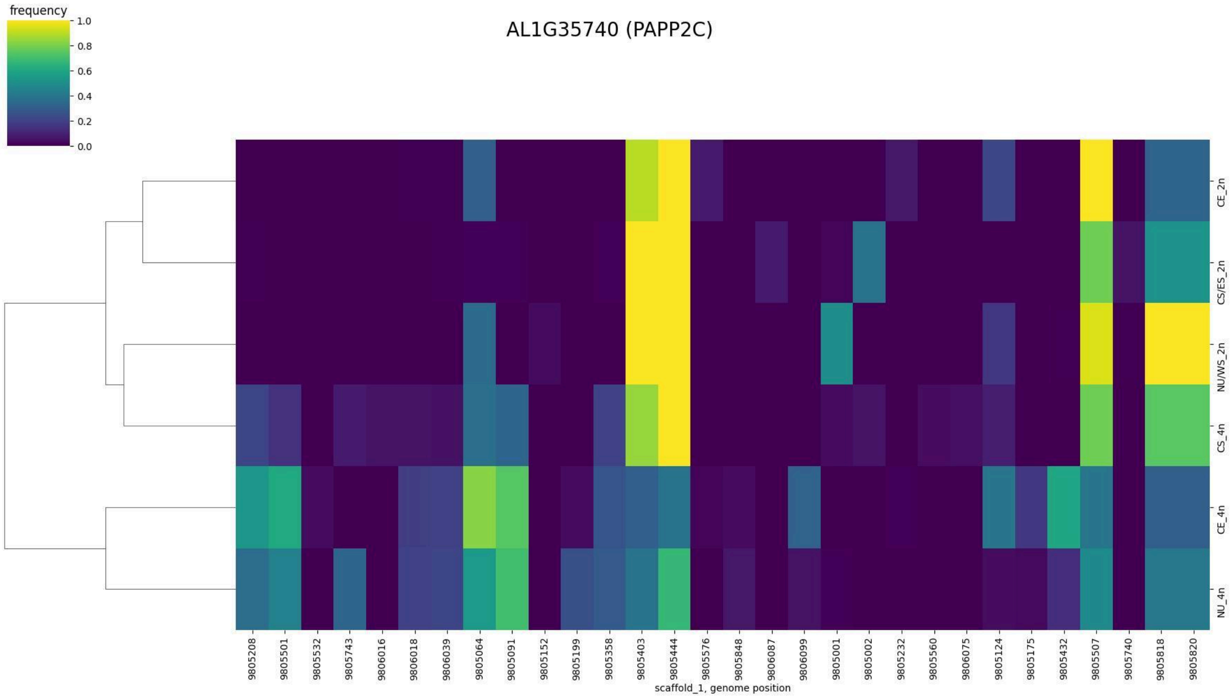
frequency
1.0
0.8
0.6
0.4
0.2
0.0
AL1G35740 (PAPP2C)
CE_2n
CS/ES_2n
NU/WS_2n
CS_4n
CE_4n
NU_4n
9805208 9805501 9805532 9805743 9806016 9806018 9806039 9805064 9805091 9805152 9805199 9805358 9805403 9805444 9805576 9805848 9806087 9806099 9805001 9805002 9805232 9805560 9806075 9805124 9805175 9805432 9805507 9805740 9805818 9805820
scaffold_1, genome position

d

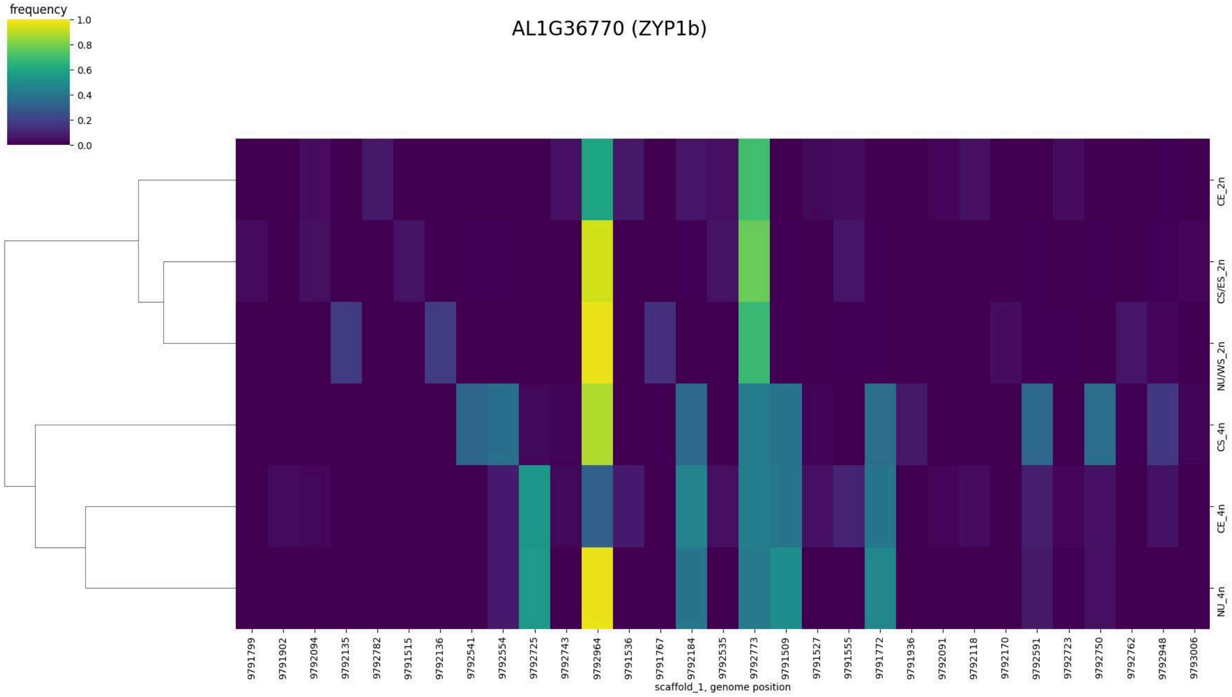
frequency
1.0
0.8
0.6
0.4
0.2
0.0
AL1G36770 (ZYP1b)
CE_2n
CS/ES_2n
NU/WS_2n
CS_4n
CE_4n
NU_4n
9791799 9791902 9792094 9792135 9792782 9791515 9792136 9792541 9792554 9792725 9792743 9792964 9791536 9791767 9792184 9792535 9792773 9791509 9791527 9791555 9791772 9791936 9792091 9792118 9792170 9792591 9792723 9792750 9792762 9792948 9793006
scaffold_1, genome position

e

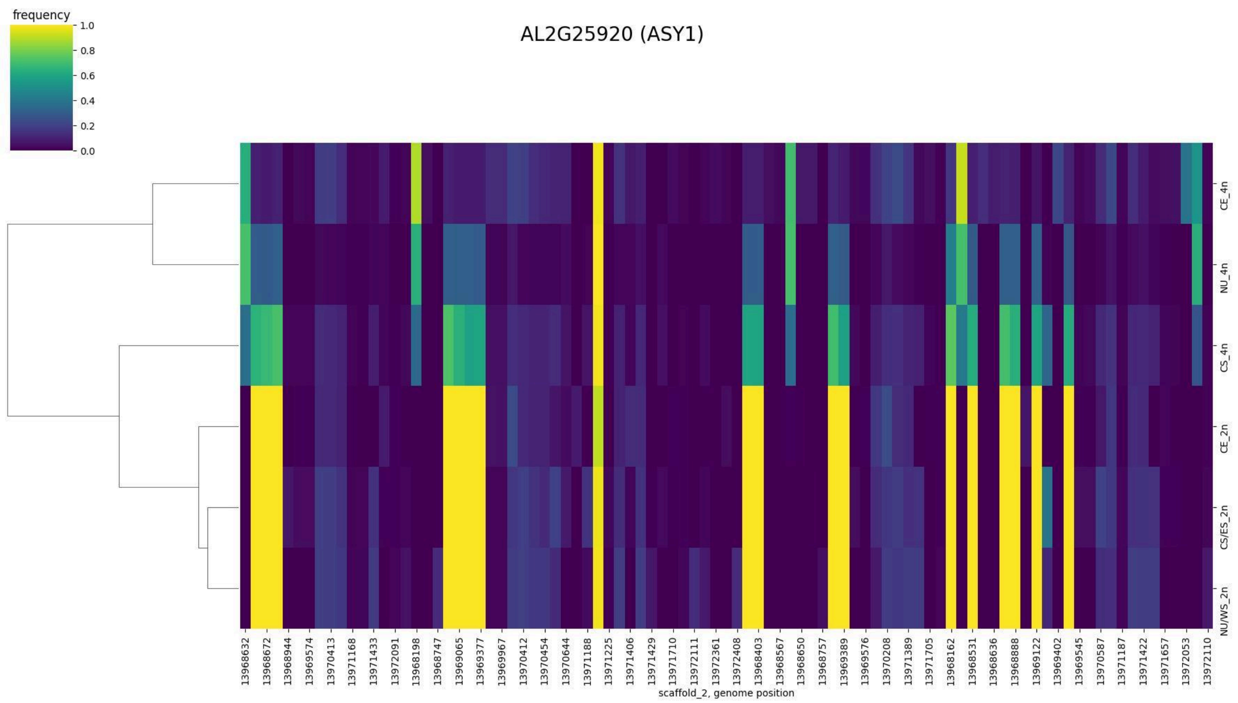

frequency
1.0
0.8
0.6
0.4
0.2
0.0
AL2G25920 (ASY1)
CE_4n
NU_4n
CS_4n
CE_2n
CS/ES_2n
NU/WS_2n
13968632 13968672 13968944 13969574 13970413 13971168 13971433 13972091 13968198 13968747 13969065 13969377 13969967 13970412 13970454 13970644 13971188 13971225 13971406 13971429 13971710 13972111 13972361 13972408 13968403 13968567 13968650 13968757 13969389 13969576 13970208 13971389 13971705 13968162 13968531 13968636 13968888 13969122 13969402 13969545 13970587 13971187 13971422 13971657 13972053 13972110
scaffold_2, genome position

f

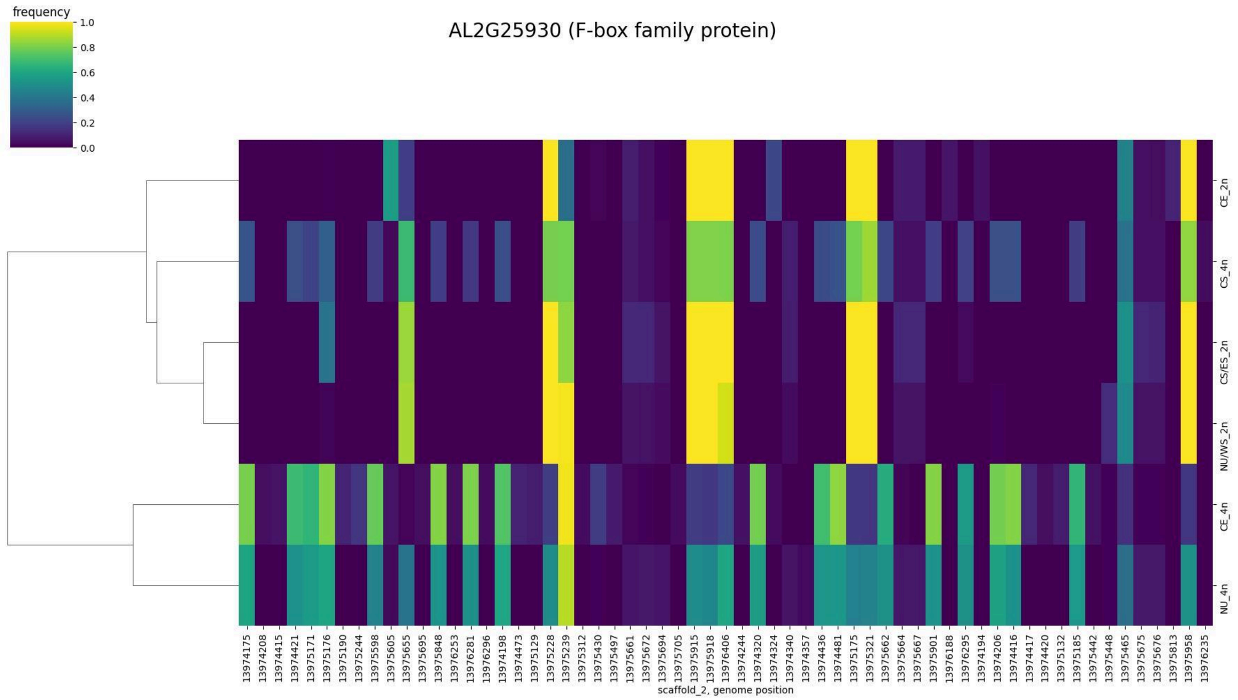

frequency
1.0
0.8
0.6
0.4
0.2
0.0
AL2G25930 (F-box family protein)
CE_2n
CS_4n
CS/ES_2n
NU/WS_2n
CE_4n
NU_4n
13974175 13974208 13974415 13974421 13975171 13975176 13975190 13975244 13975598 13975605 13975655 13975695 13975848 13976253 13976281 13976296 13974198 13974473 13975129 13975228 13975239 13975312 13975430 13975497 13975661 13975672 13975694 13975705 13975915 13975918 13976406 13974244 13974320 13974324 13974340 13974357 13974436 13974481 13975175 13975321 13975662 13975664 13975667 13975901 13976188 13976295 13974194 13974206 13974416 13974417 13974420 13975132 13975185 13975442 13975448 13975465 13975675 13975676 13975813 13975958 13976235
scaffold_2, genome position

g

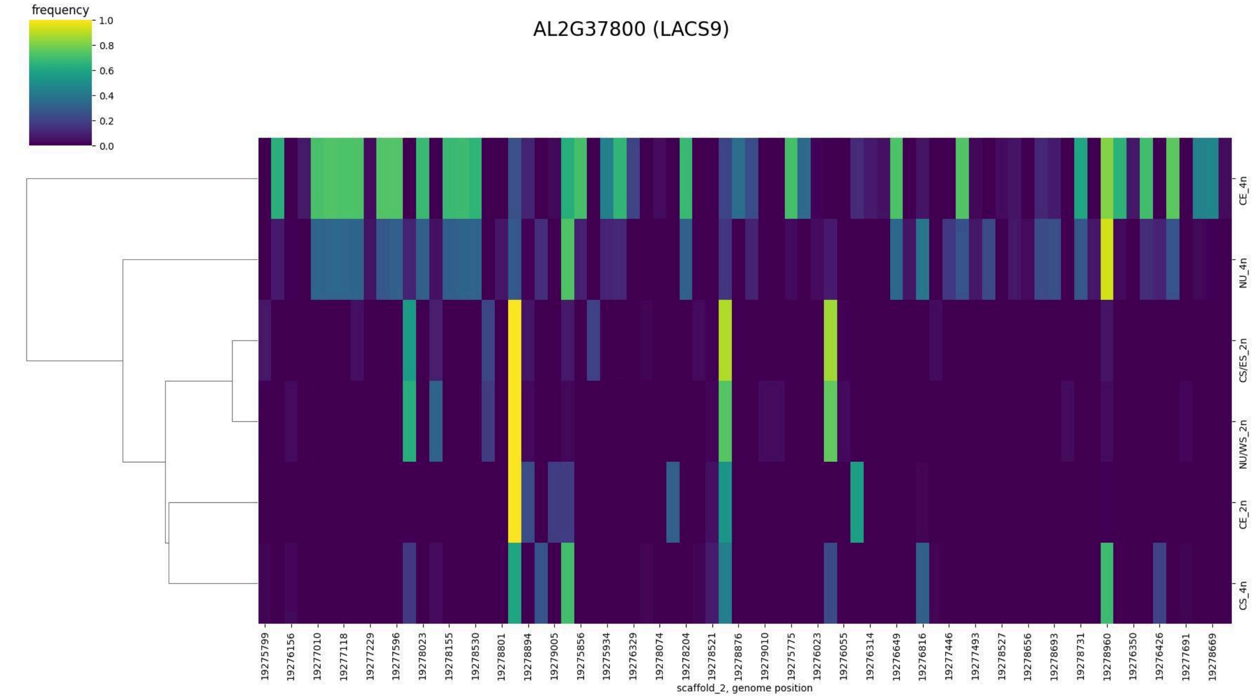
AL2G37800 (LACS9)
frequency
1.0
0.8
0.6
0.4
0.2
0.0
CE_4n
NU_4n
CS/ES_2n
NU/WS_2n
CE_2n
CS_4n
19275799
19276156
19277010
19277118
19277229
19277596
19278023
19278155
19278530
19278801
19278894
19279005
19275856
19275934
19276329
19278074
19278204
19278521
19278876
19279010
19275775
19276023
19276055
19276314
19276649
19276816
19277446
19277493
19278527
19278656
19278693
19278731
19278960
19276350
19276426
19277691
19278669
scaffold_2, genome position

h

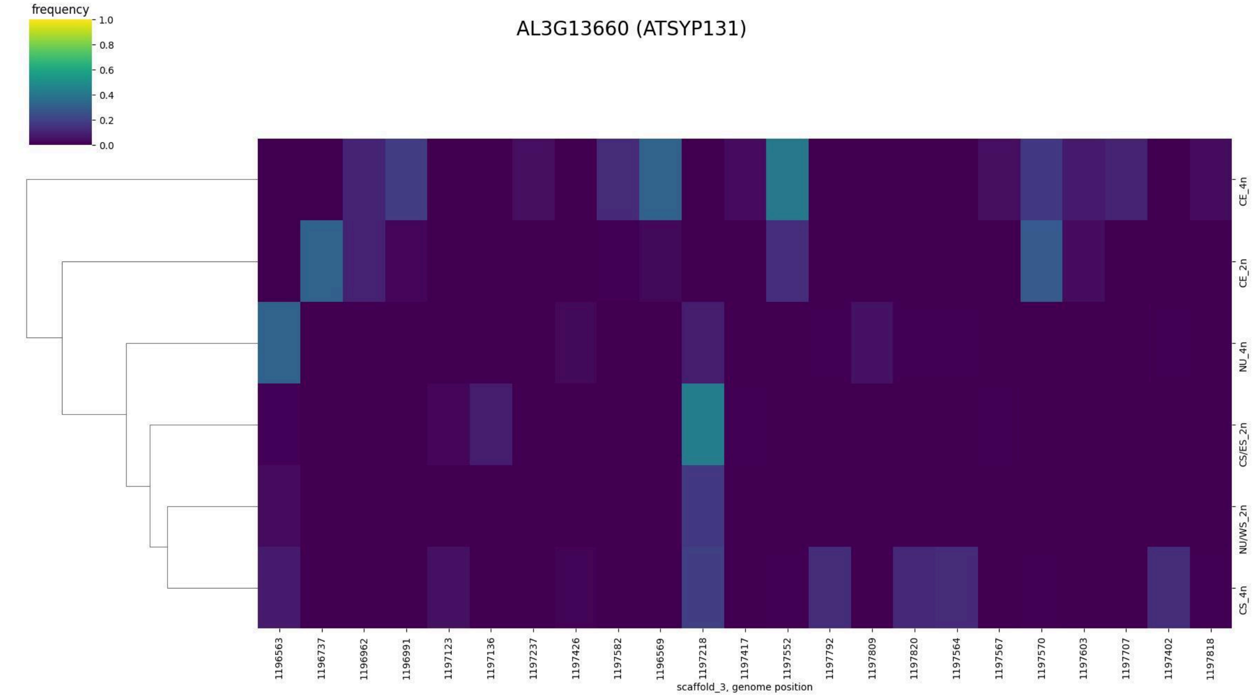
AL3G13660 (ATSYP131)
frequency
1.0
0.8
0.6
0.4
0.2
0.0
CE_4n
CE_2n
NU_4n
CS/ES_2n
NU/WS_2n
CS_4n
1196563
1196737
1196962
1196991
1197123
1197136
1197237
1197426
1197582
1196569
1197218
1197417
1197552
1197792
1197809
1197820
1197564
1197567
1197570
1197603
1197707
1197402
1197818
scaffold_3, genome position

i

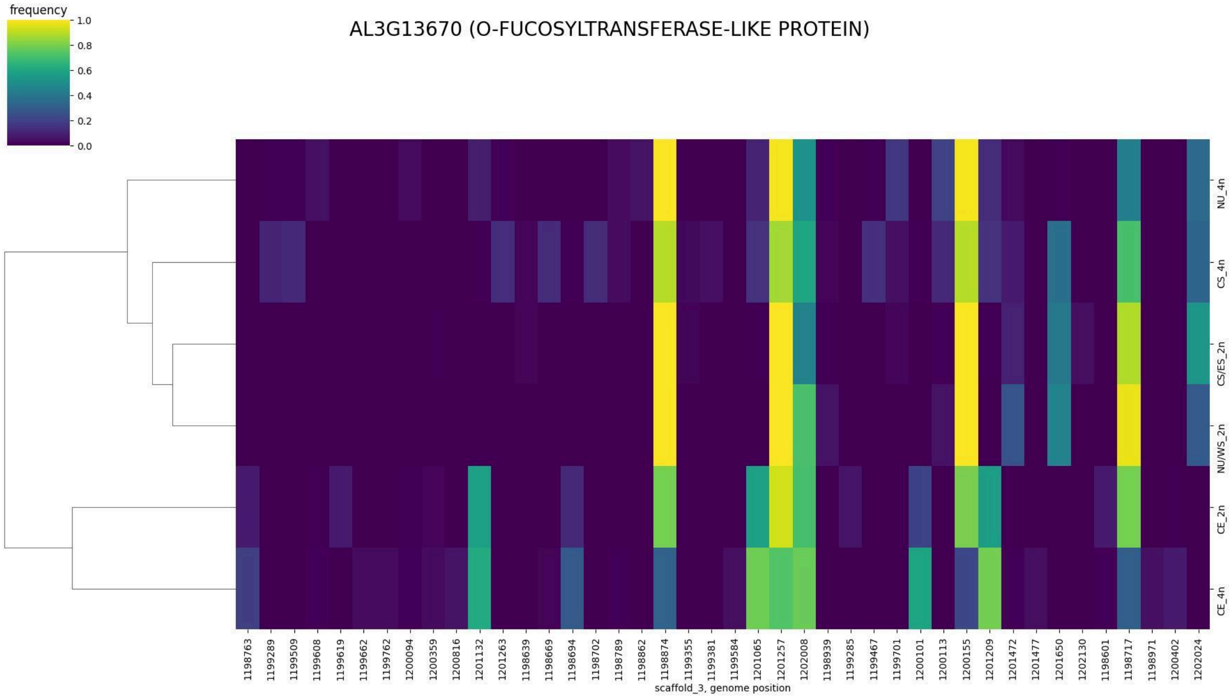
frequency
1.0
0.8
0.6
0.4
0.2
0.0
AL3G13670 (O-FUCOSYLTRANSFERASE-LIKE PROTEIN)
NU_4n
CS_4n
CS/ES_2n
NU/WS_2n
CE_2n
CE_4n
scaffold_3, genome position

j

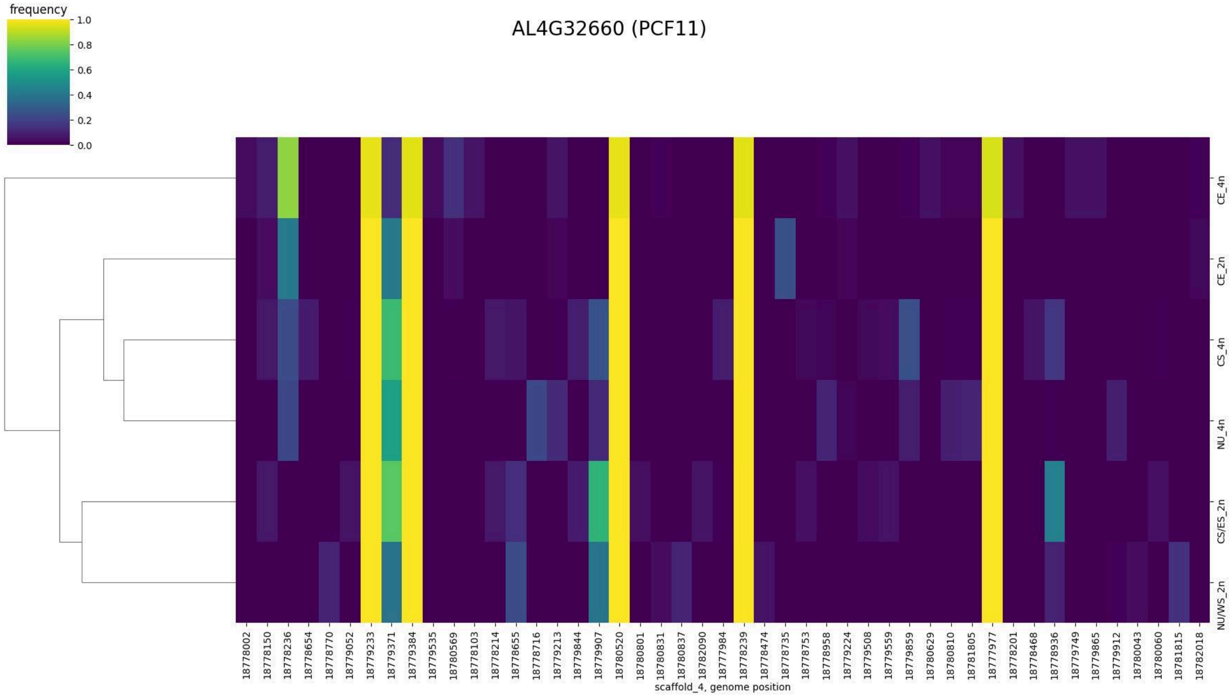
frequency
1.0
0.8
0.6
0.4
0.2
0.0
AL4G32660 (PCF11)
CE_4n
CE_2n
CS_4n
NU_4n
CS/ES_2n
NU/WS_2n
scaffold_4, genome position

k

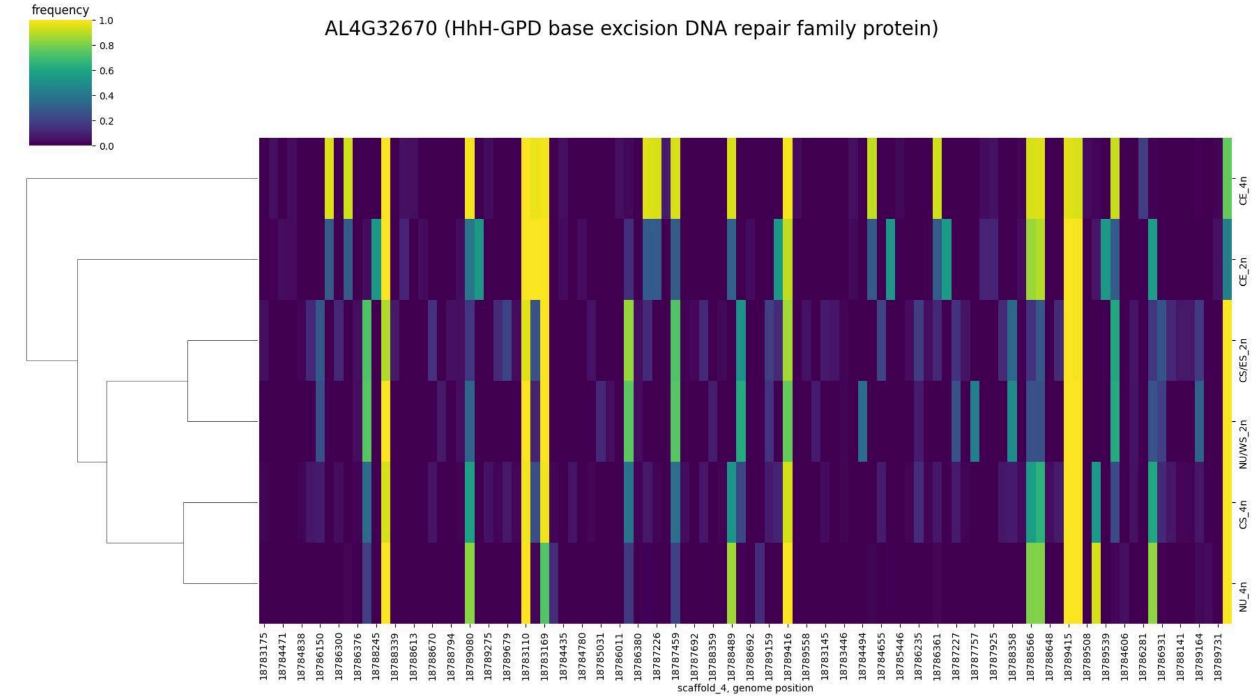
frequency
1.0
0.8
0.6
0.4
0.2
0.0
AL4G32670 (HhH-GPD base excision DNA repair family protein)
CE_4n
CE_2n
CS/ES_2n
NU/WS_2n
CS_4n
NU_4n
18783175 18784471 18784838 18786150 18786300 18786376 18788245 18788339 18788613 18788670 18788794 18789080 18789275 18789679 18783110 18783169 18784435 18784780 18785031 18786011 18786380 18787226 18787459 18787692 18788359 18788489 18788692 18789159 18789416 18789558 18783145 18783446 18784494 18784655 18785446 18786235 18786361 18787227 18787757 18787925 18788358 18788566 18788648 18789415 18789508 18789539 18784606 18786281 18786931 18788141 18789164 18789731
scaffold_4, genome position

l

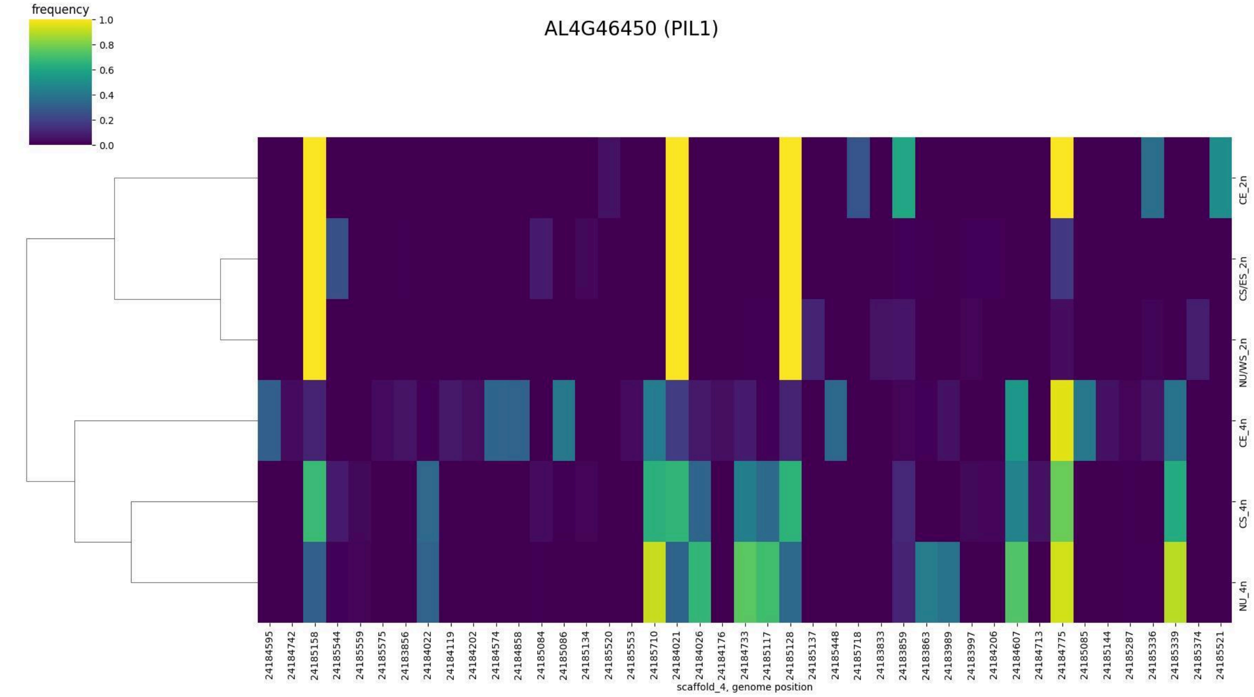
frequency
1.0
0.8
0.6
0.4
0.2
0.0
AL4G46450 (PIL1)
CE_2n
CS/ES_2n
NU/WS_2n
CE_4n
CS_4n
NU_4n
24184595 24184742 24185158 24185544 24185559 24185575 24183856 24184022 24184119 24184202 24184574 24184858 24185084 24185086 24185134 24185520 24185553 24185710 24184021 24184026 24184176 24184733 24185117 24185128 24185137 24185448 24185718 24183833 24183859 24183863 24183989 24183997 24184206 24184607 24184713 24184775 24185085 24185144 24185287 24185336 24185339 24185374 24185521
scaffold_4, genome position

m

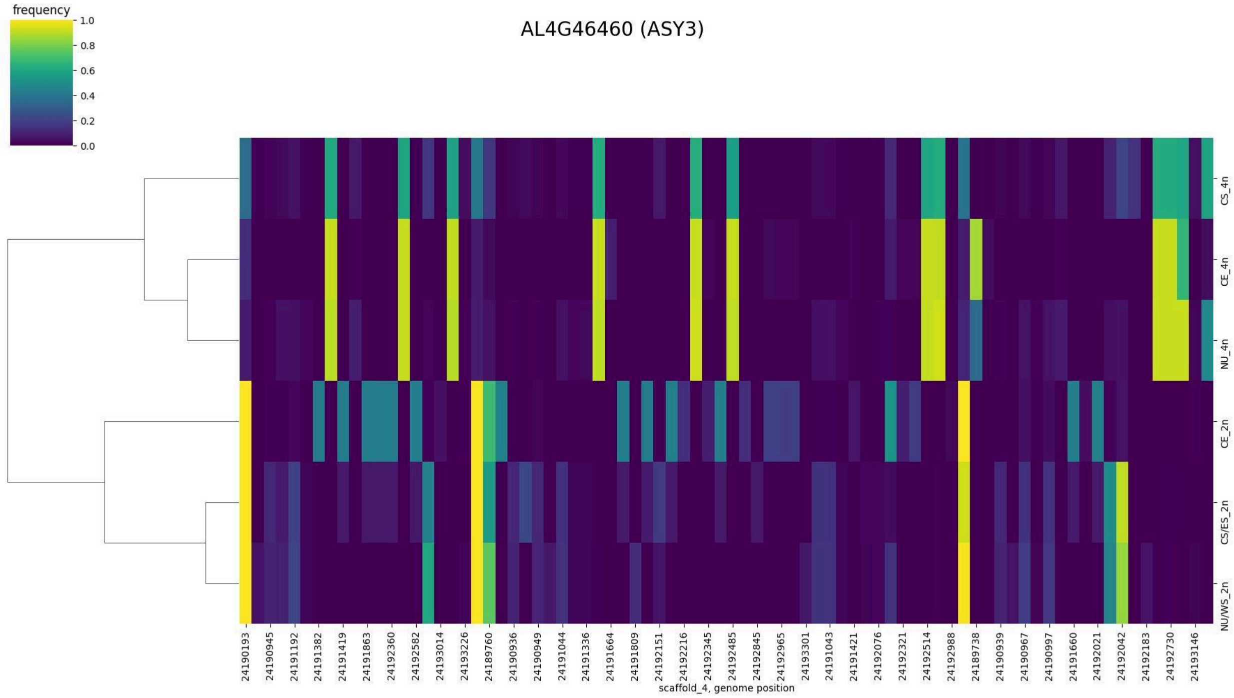
frequency
1.0
0.8
0.6
0.4
0.2
0.0
AL4G46460 (ASY3)
CS_4n
CE_4n
NU_4n
CE_2n
CS/ES_2n
NU/WS_2n
24190193
24190945
24191192
24191382
24191419
24191863
24192360
24192582
24193014
24193226
24189760
24190936
24190949
24191044
24191336
24191664
24191809
24192151
24192216
24192345
24192485
24192845
24192965
24193301
24191043
24191421
24192076
24192321
24192514
24192988
24189738
24190939
24190967
24190997
24191660
24192021
24192042
24192183
24192730
24193146
scaffold_4, genome position

n

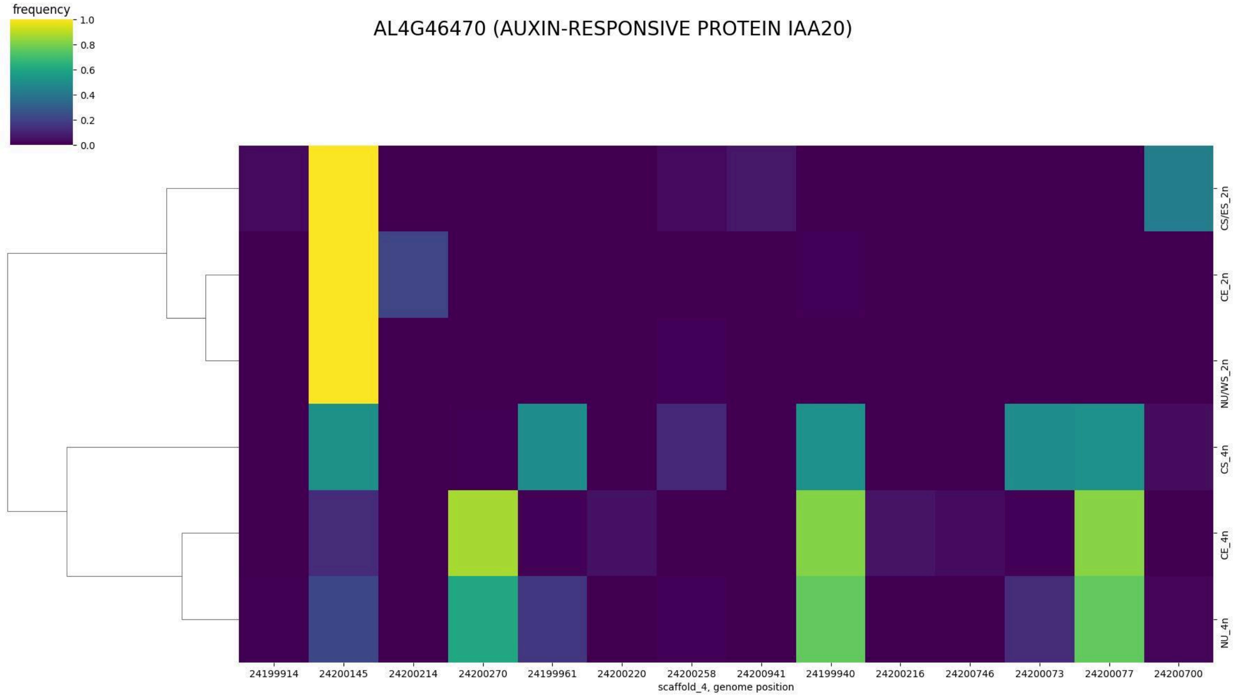
frequency
1.0
0.8
0.6
0.4
0.2
0.0
AL4G46470 (AUXIN-RESPONSIVE PROTEIN IAA20)
CS/ES_2n
CE_2n
NU/WS_2n
CS_4n
CE_4n
NU_4n
24199914
24200145
24200214
24200270
24199961
24200220
24200258
24200941
24199940
24200216
24200746
24200073
24200077
24200700
scaffold_4, genome position

o

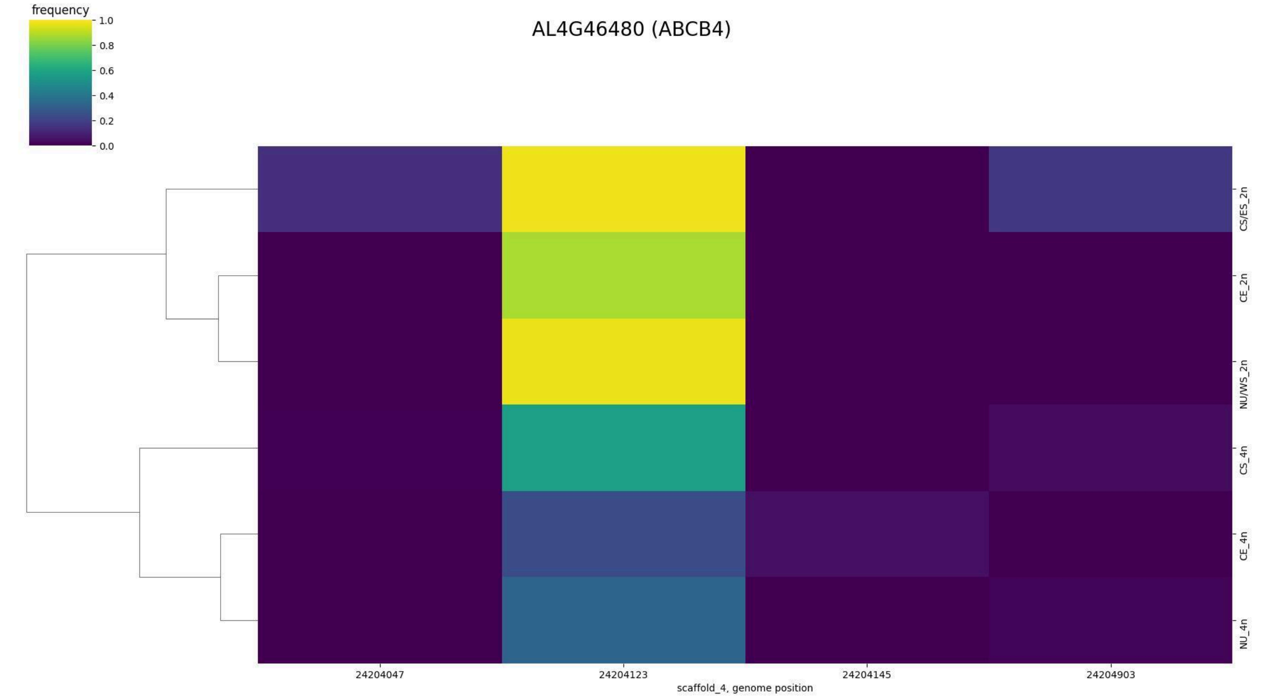
frequency
1.0
0.8
0.6
0.4
0.2
0.0
AL4G46480 (ABCB4)
CS/ES_2n
CE_2n
NU/WS_2n
CS_4n
CE_4n
NU_4n
24204047
24204123
24204145
24204903
scaffold_4, genome position

p

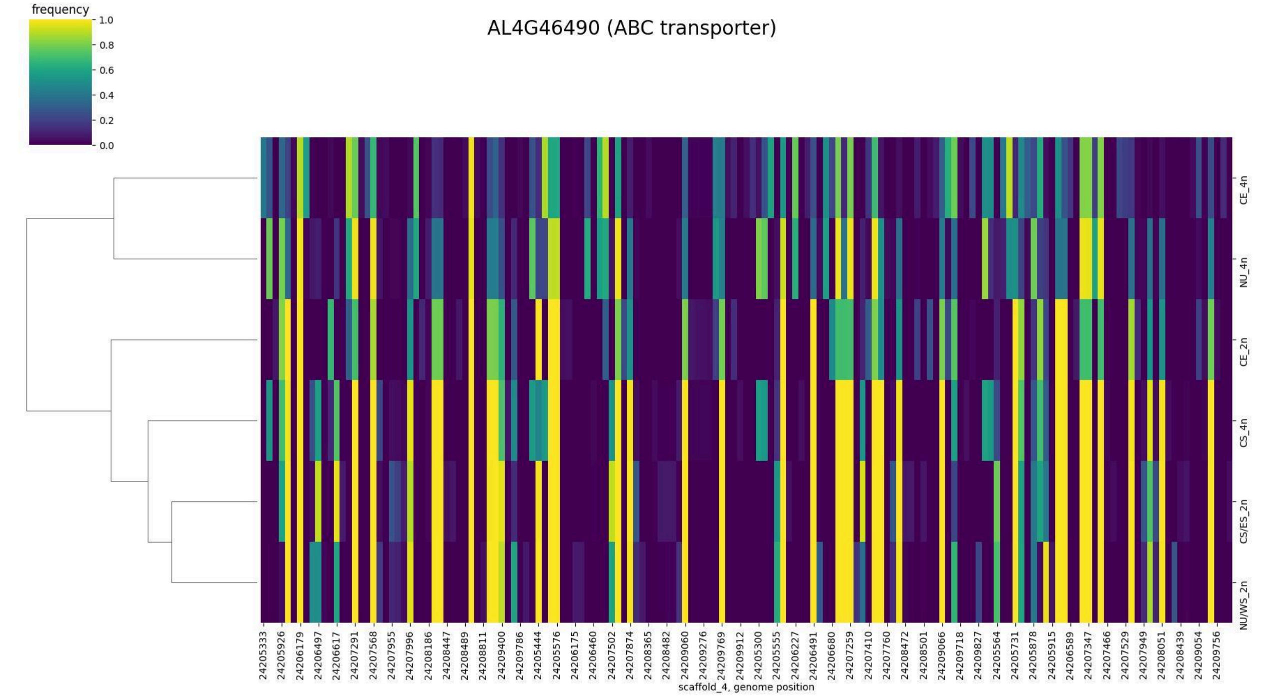
frequency
1.0
0.8
0.6
0.4
0.2
0.0
AL4G46490 (ABC transporter)
CE_4n
NU_4n
CE_2n
CS_4n
CS/ES_2n
NU/WS_2n
24205333 24205926 24206179 24206497 24206617 24207291 24207568 24207955 24207996 24208186 24208447 24208489 24208811 24209400 24209786 24205444 24205576 24206175 24206460 24207502 24207874 24208365 24208482 24209060 24209276 24209769 24209912 24205300 24205555 24206227 24206491 24206680 24207259 24207410 24207760 24208472 24208501 24209066 24209718 24209827 24205564 24205731 24205878 24205915 24206589 24207347 24207466 24207529 24207949 24208051 24208439 24209054 24209756
scaffold_4, genome position

q

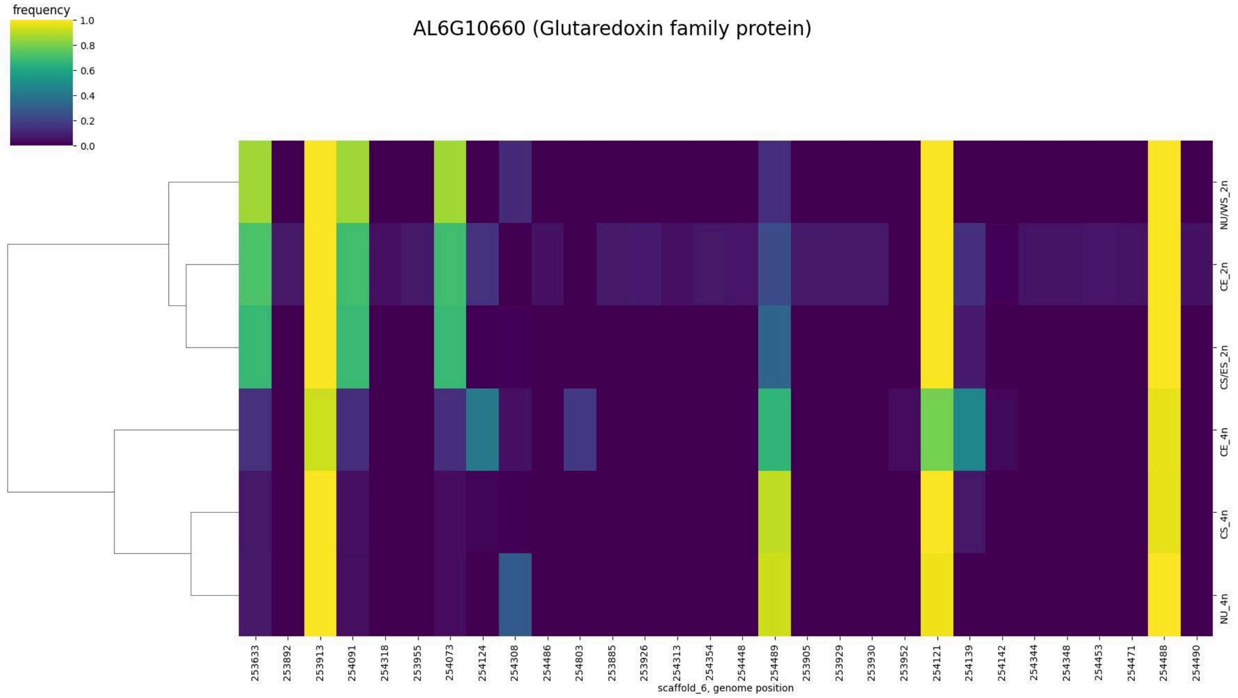
AL6G10660 (Glutaredoxin family protein)
frequency
1.0
0.8
0.6
0.4
0.2
0.0
NU/WS_2n
CE_2n
CS/ES_2n
CE_4n
CS_4n
NU_4n
253633 253892 253913 254091 254318 253955 254073 254124 254308 254486 254803 253885 253926 254313 254354 254448 254489 253905 253929 253930 253952 254121 254139 254142 254344 254348 254453 254471 254488 254490
scaffold_6, genome position

r

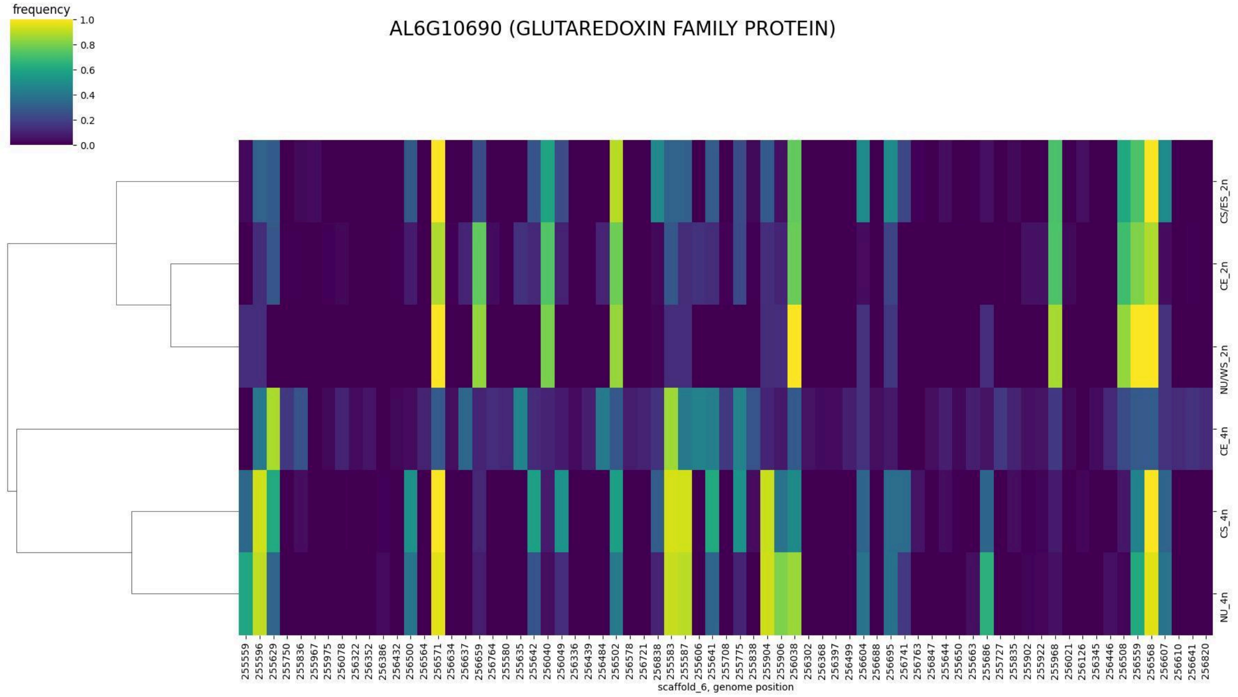
AL6G10690 (GLUTAREDOXIN FAMILY PROTEIN)
frequency
1.0
0.8
0.6
0.4
0.2
0.0
CS/ES_2n
CE_2n
NU/WS_2n
CE_4n
CS_4n
NU_4n
scaffold_6, genome position

s

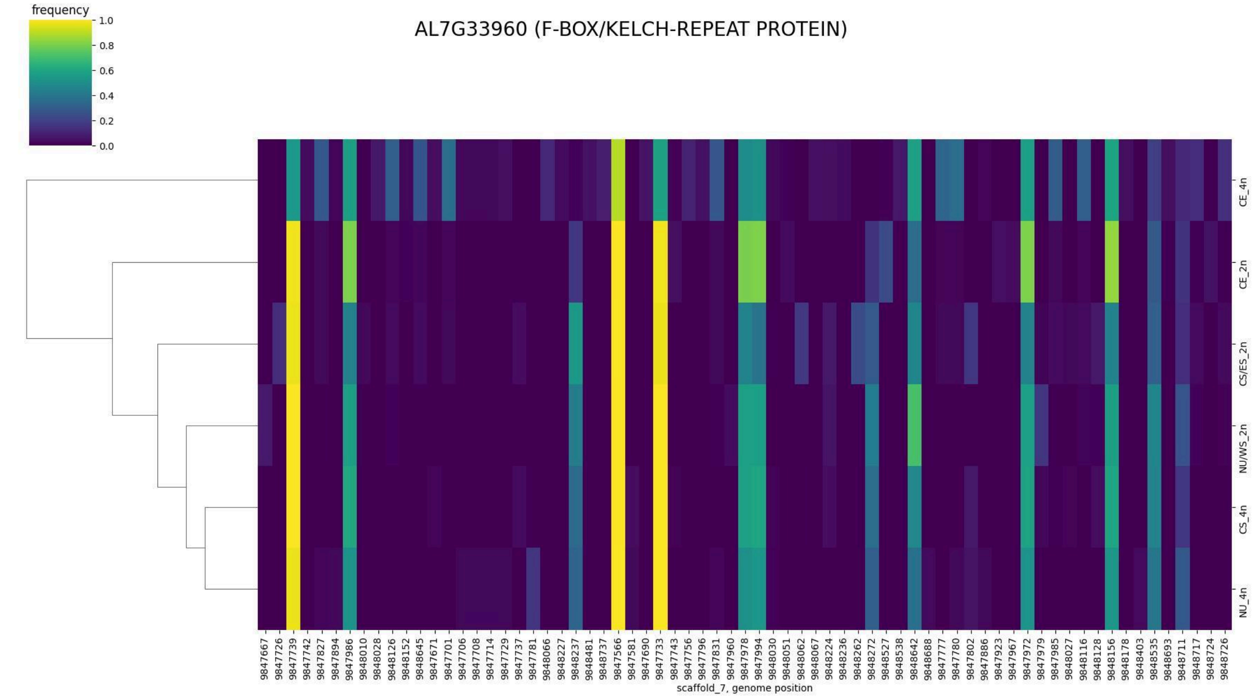
AL7G33960 (F-BOX/KELCH-REPEAT PROTEIN)
frequency
1.0
0.8
0.6
0.4
0.2
0.0
CE_4n
CE_2n
CS/ES_2n
NU/WS_2n
CS_4n
NU_4n
9847667 9847726 9847739 9847742 9847827 9847894 9847986 9848010 9848028 9848126 9848152 9848645 9847671 9847701 9847706 9847708 9847714 9847729 9847737 9847781 9848066 9848227 9848237 9848481 9848737 9847566 9847581 9847690 9847733 9847743 9847756 9847796 9847831 9847960 9847978 9847994 9848030 9848051 9848062 9848067 9848224 9848236 9848262 9848272 9848527 9848538 9848642 9848688 9847777 9847780 9847802 9847886 9847923 9847967 9847972 9847979 9847985 9848027 9848116 9848128 9848156 9848178 9848403 9848535 9848693 9848711 9848717 9848724 9848726
scaffold_7, genome position

t

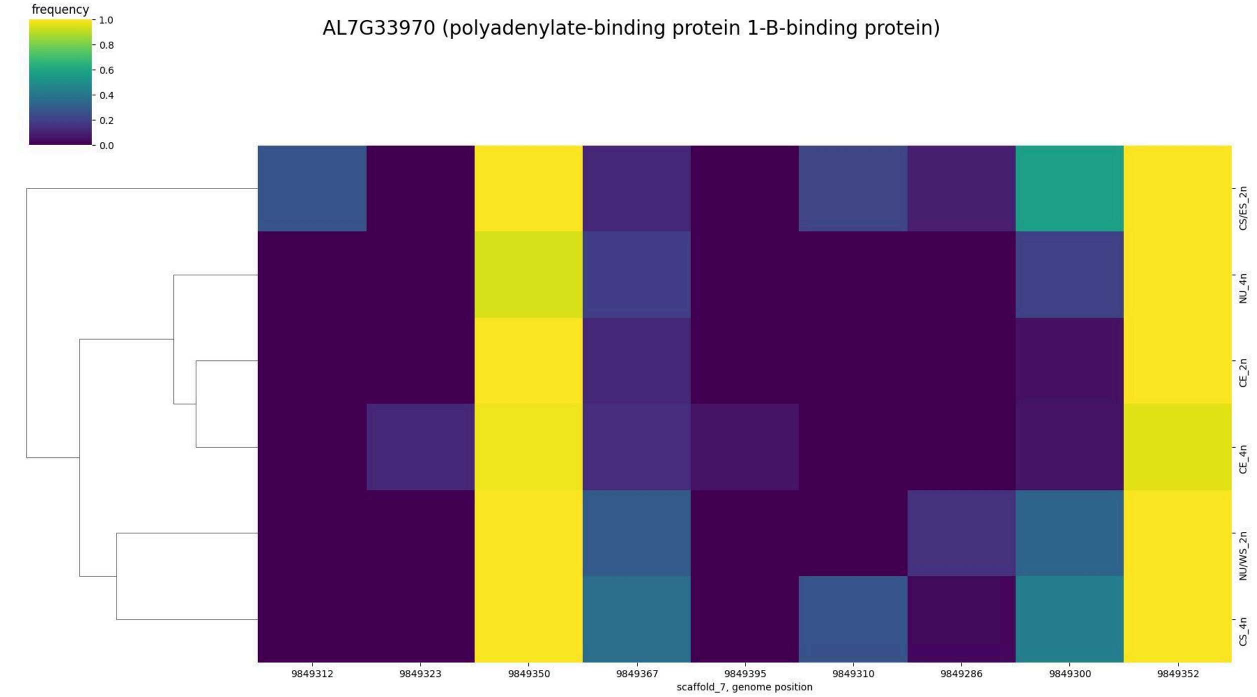
AL7G33970 (polyadenylate-binding protein 1-B-binding protein)
frequency
1.0
0.8
0.6
0.4
0.2
0.0
CS/ES_2n
NU_4n
CE_2n
CE_4n
NU/WS_2n
CS_4n
9849312
9849323
9849350
9849367
9849395
9849310
9849286
9849300
9849352
scaffold_7, genome position

u

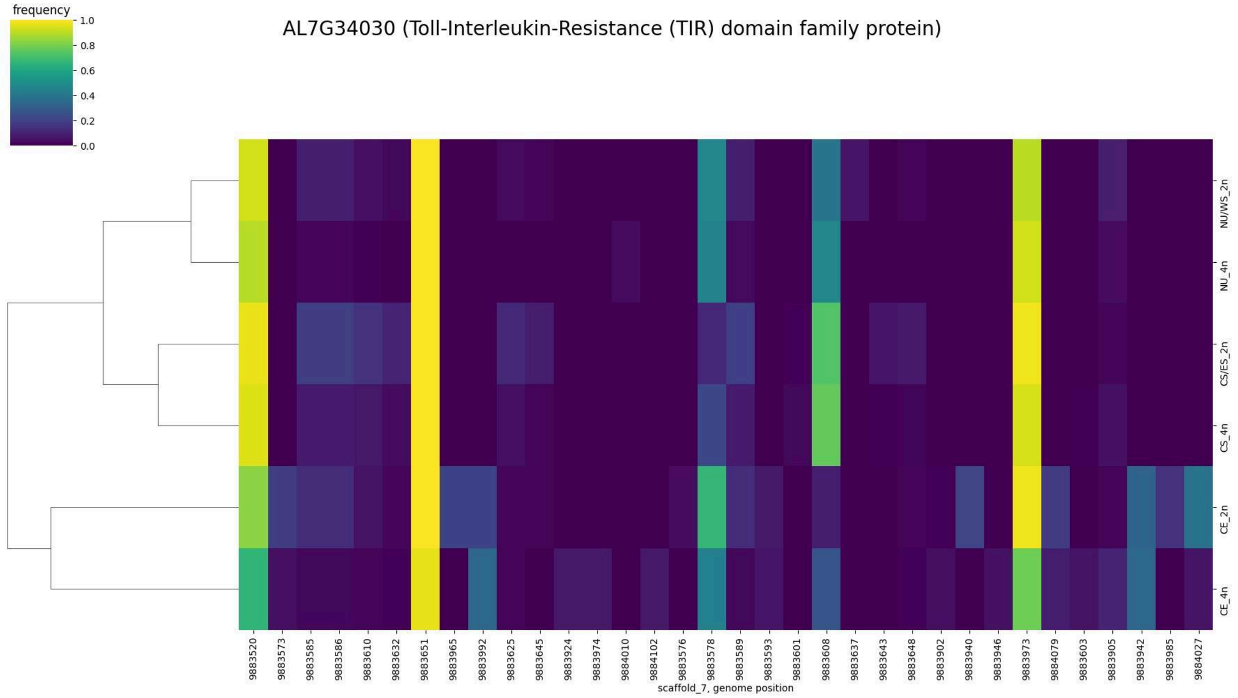

AL7G34030 (Toll-Interleukin-Resistance (TIR) domain family protein)
frequency
1.0
0.8
0.6
0.4
0.2
0.0
NU/WS_2n
NU_4n
CS/ES_2n
CS_4n
CE_2n
CE_4n
9883520 9883573 9883585 9883586 9883610 9883632 9883651 9883965 9883992 9883625 9883645 9883924 9883974 9884010 9884102 9883576 9883578 9883589 9883593 9883601 9883608 9883637 9883643 9883648 9883902 9883940 9883946 9883973 9884079 9883603 9883905 9883942 9883985 9884027
scaffold_7, genome position

V

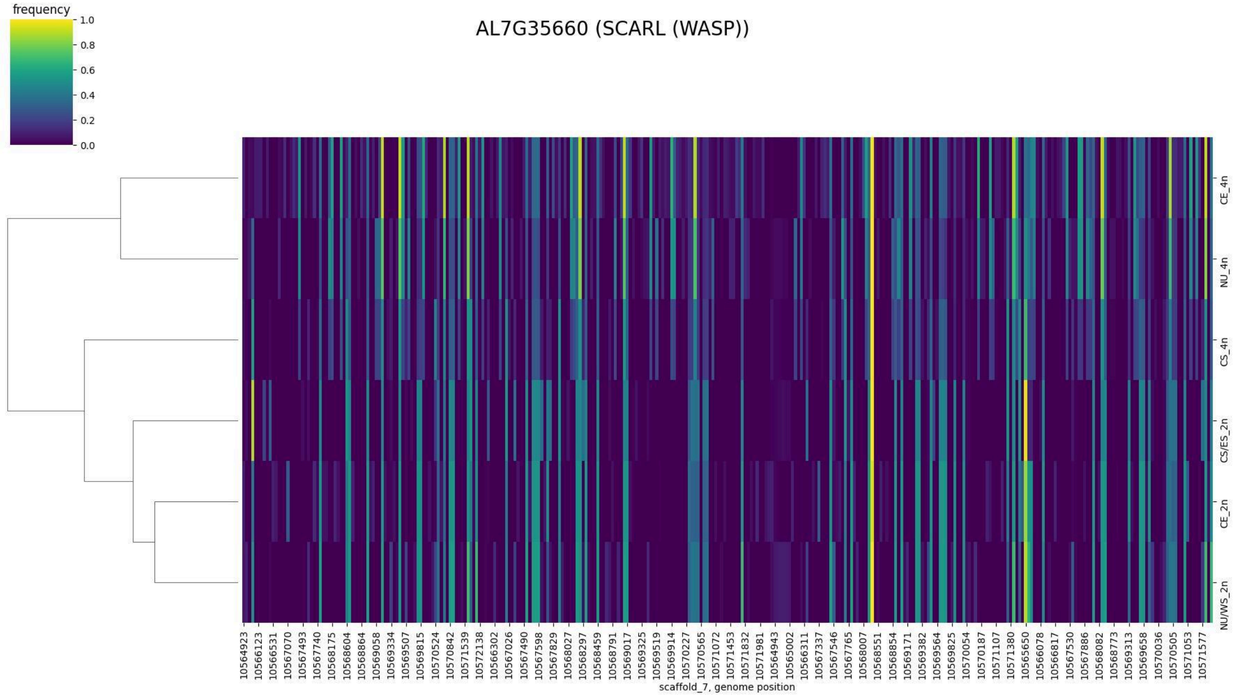

AL7G35660 (SCARL (WASP))
frequency
1.0
0.8
0.6
0.4
0.2
0.0
CE_4n
NU_4n
CS_4n
CS/ES_2n
CE_2n
NU/WS_2n
scaffold_7, genome position

W

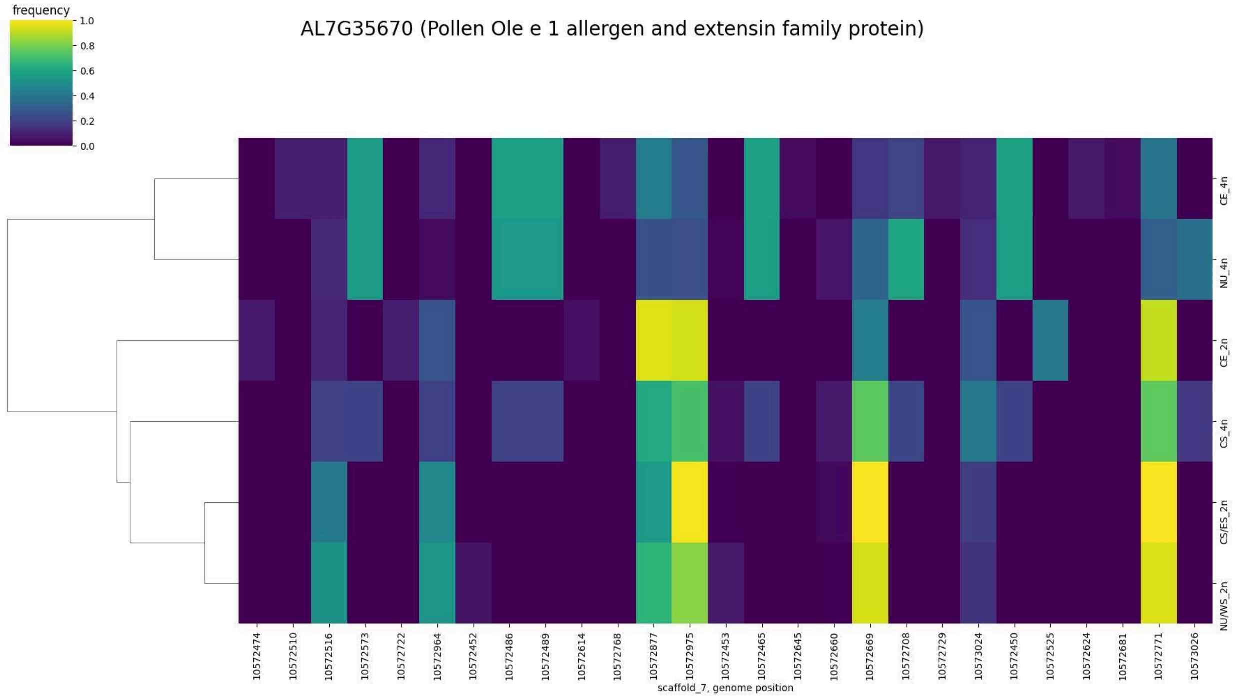
frequency
AL7G35670 (Pollen Ole e 1 allergen and extensin family protein)
CE_4n
NU_4n
CE_2n
CS_4n
CS/ES_2n
NU/WS_2n
10572474 10572510 10572516 10572573 10572722 10572964 10572452 10572486 10572489 10572614 10572768 10572877 10572975 10572453 10572465 10572645 10572660 10572669 10572708 10572729 10573024 10572450 10572525 10572624 10572681 10572771 10573026
scaffold_7, genome position

x

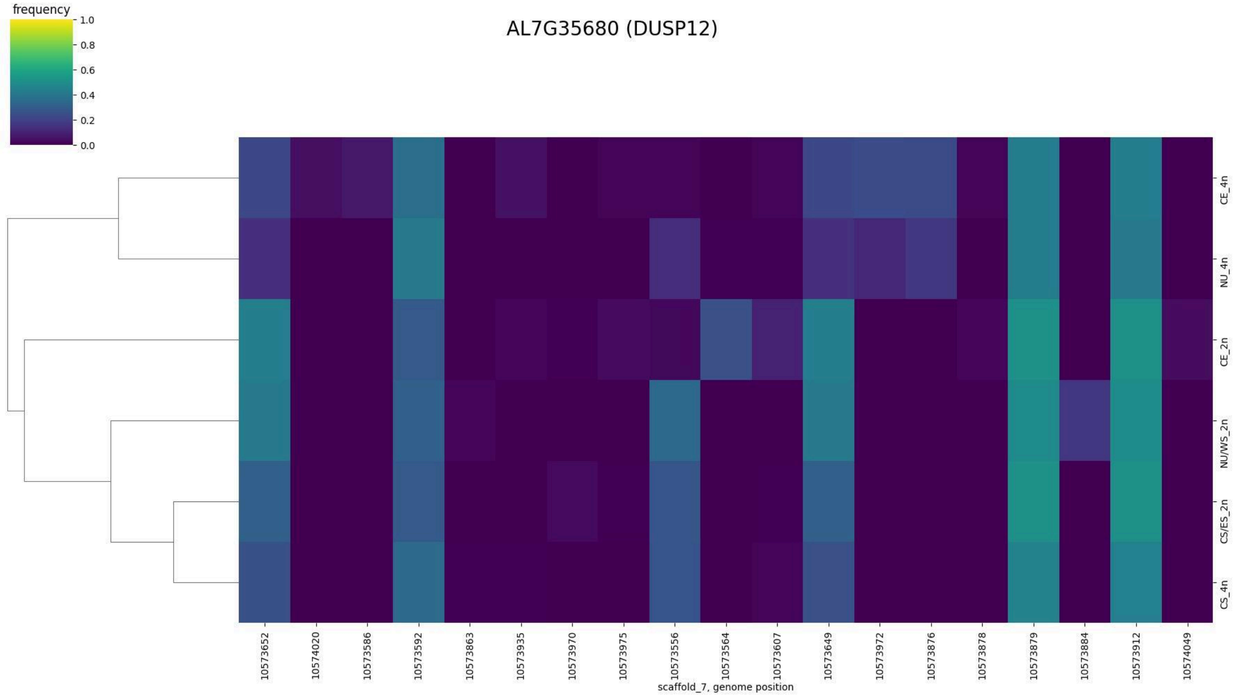
frequency
AL7G35680 (DUSP12)
CE_4n
NU_4n
CE_2n
NU/WS_2n
CS/ES_2n
CS_4n
10573652 10574020 10573586 10573592 10573863 10573935 10573970 10573975 10573556 10573564 10573607 10573649 10573972 10573876 10573878 10573879 10573884 10573912 10574049
scaffold_7, genome position

y

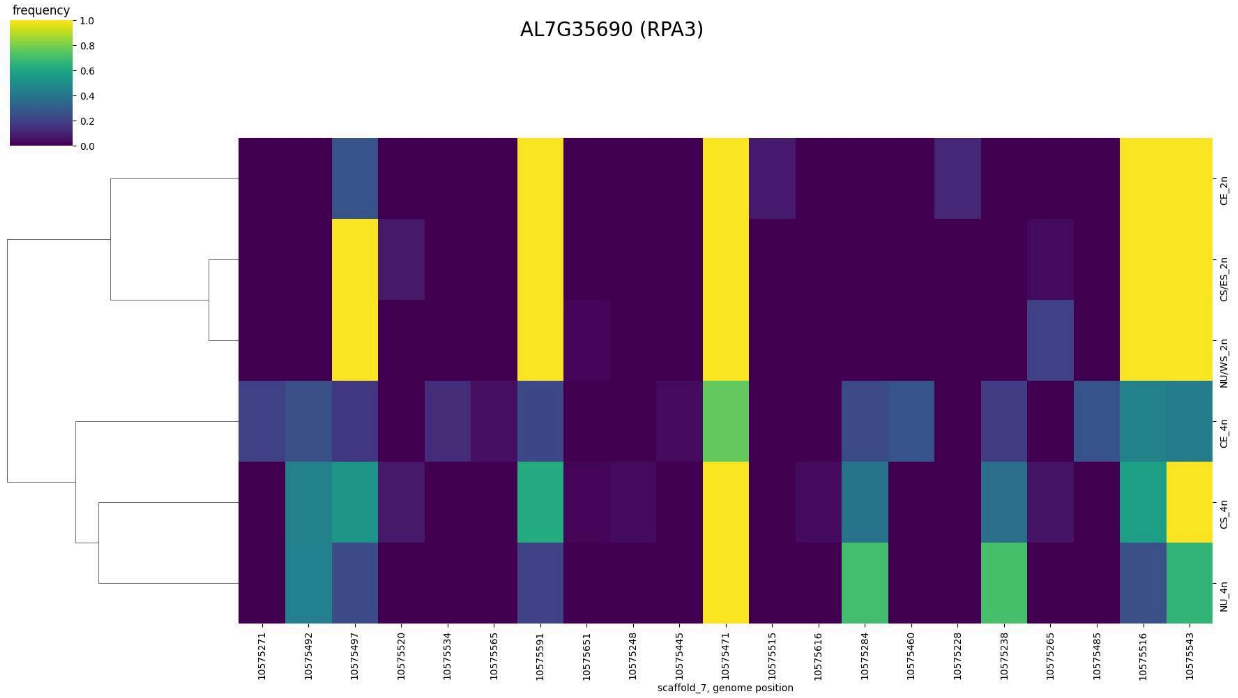

frequency
1.0
0.8
0.6
0.4
0.2
0.0
AL7G35690 (RPA3)
CE_2n
CS/ES_2n
NU/WS_2n
CE_4n
CS_4n
NU_4n
10575271
10575492
10575497
10575520
10575534
10575565
10575591
10575651
10575248
10575445
10575471
10575515
10575616
10575284
10575460
10575228
10575238
10575265
10575485
10575516
10575543
scaffold_7, genome position

z

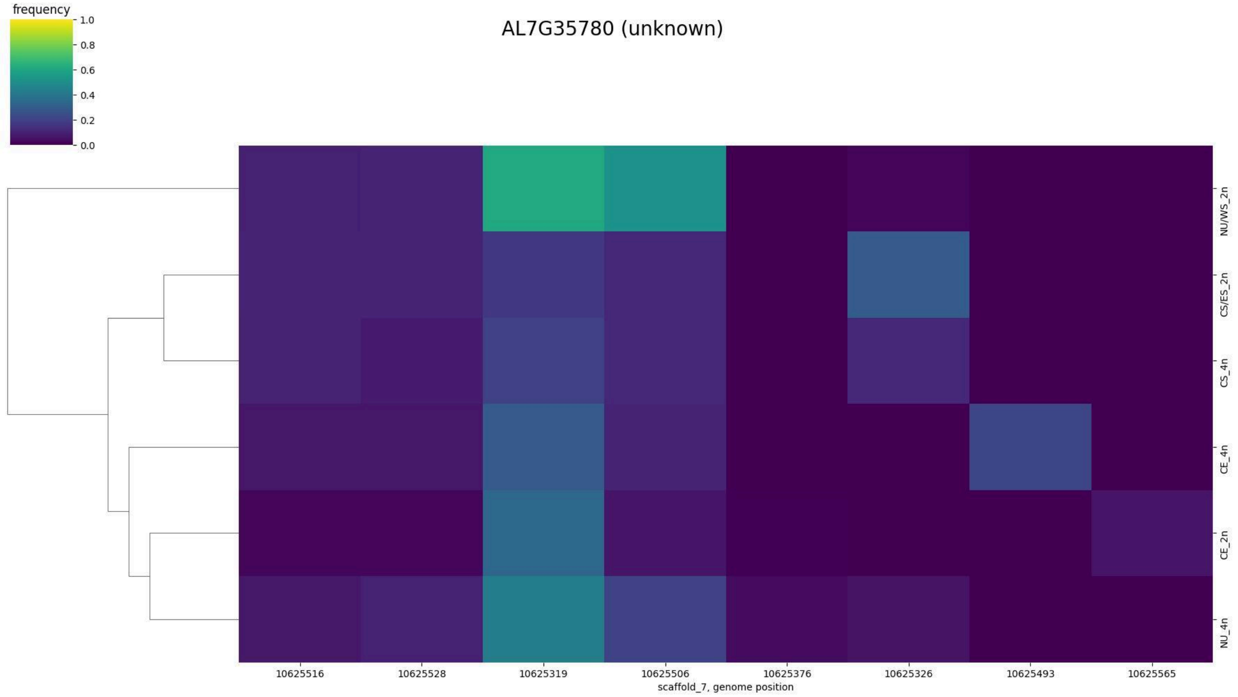

frequency
1.0
0.8
0.6
0.4
0.2
0.0
AL7G35780 (unknown)
NU/WS_2n
CS/ES_2n
CS_4n
CE_4n
CE_2n
NU_4n
10625516
10625528
10625319
10625506
10625376
10625326
10625493
10625565
scaffold_7, genome position

aa

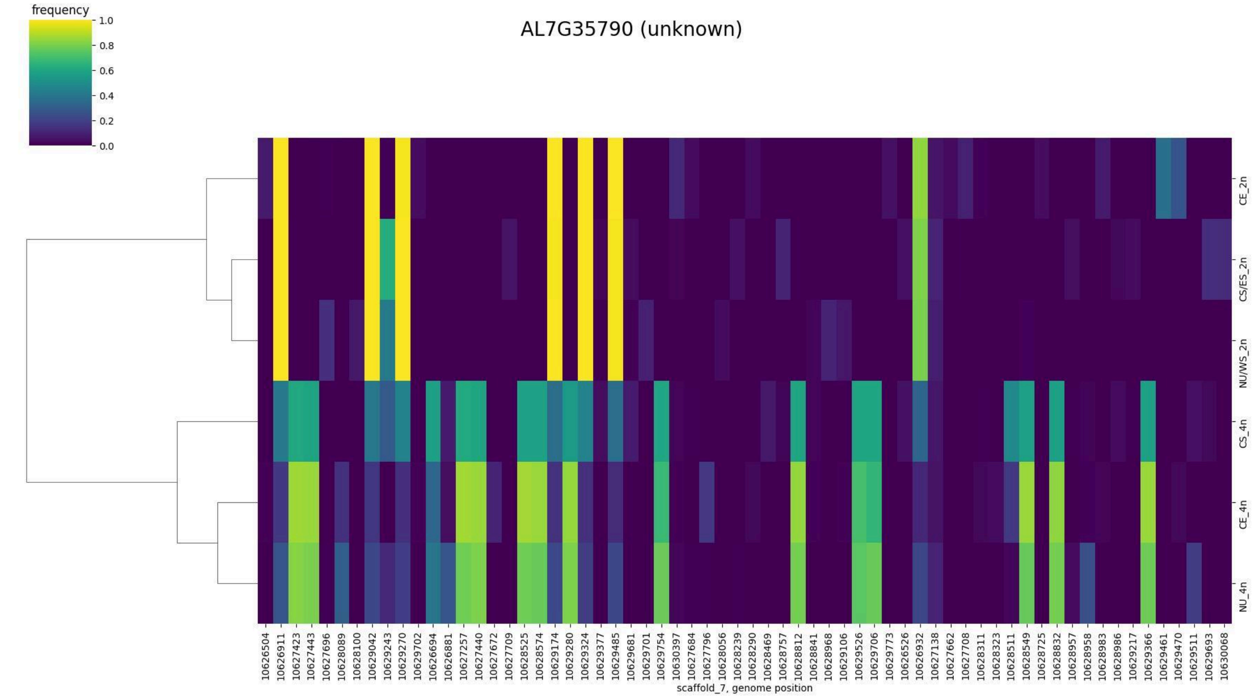
AL7G35790 (unknown)
frequency
1.0
0.8
0.6
0.4
0.2
0.0
CE_2n
CS/ES_2n
NU/WS_2n
CS_4n
CE_4n
NU_4n
10626504 10626911 10627423 10627443 10627696 10628089 10628100 10629042 10629243 10629270 10629702 10626694 10626881 10627257 10627440 10627672 10627709 10628525 10628574 10629174 10629280 10629324 10629377 10629485 10629681 10629701 10629754 10630397 10627684 10627796 10628056 10628239 10628290 10628469 10628757 10628812 10628841 10628968 10629106 10629526 10629706 10629773 10626526 10626932 10627138 10627662 10627708 10628311 10628323 10628511 10628549 10628725 10628832 10628957 10628958 10628983 10628986 10629217 10629366 10629461 10629470 10629511 10629693 10630068
scaffold_7, genome position

bb

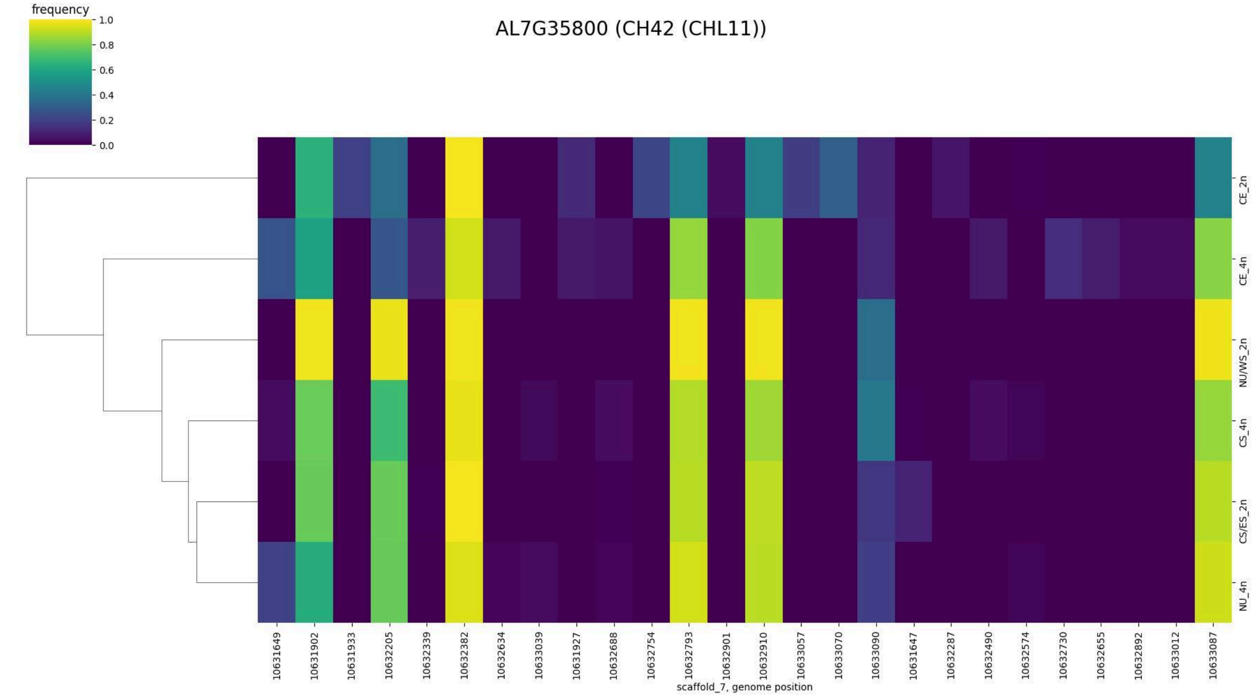
AL7G35800 (CH42 (CHL11))
frequency
1.0
0.8
0.6
0.4
0.2
0.0
CE_2n
CE_4n
NU/WS_2n
CS_4n
CS/ES_2n
NU_4n
10631649 10631902 10631933 10632205 10632339 10632382 10632634 10633039 10631927 10632688 10632754 10632793 10632901 10632910 10633057 10633070 10633090 10631647 10632287 10632490 10632574 10632730 10632655 10632892 10633012 10633087
scaffold_7, genome position

cc

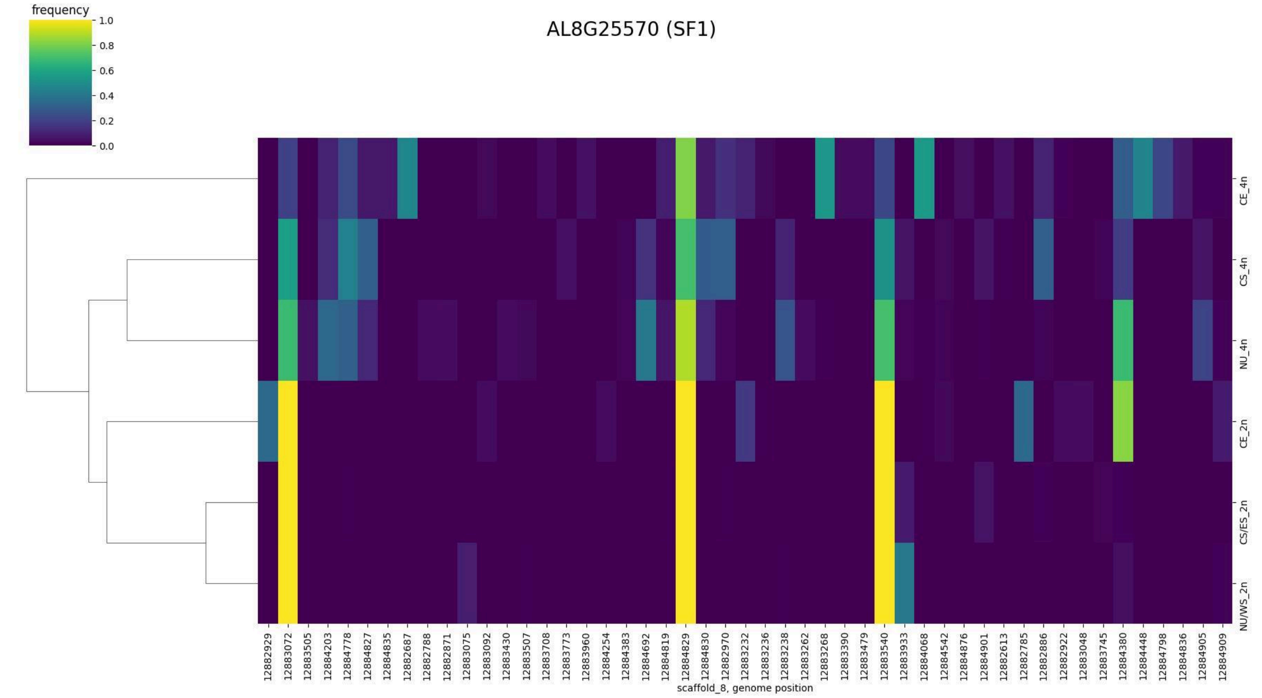
AL8G25570 (SF1)
frequency
1.0
0.8
0.6
0.4
0.2
0.0
CE_4n
CS_4n
NU_4n
CE_2n
CS/ES_2n
NU/WS_2n
12882929 12883072 12883505 12884203 12884778 12884827 12884835 12882687 12882788 12882871 12883075 12883092 12883430 12883507 12883708 12883773 12883960 12884254 12884383 12884692 12884819 12884829 12884830 12882970 12883232 12883236 12883238 12883262 12883268 12883390 12883479 12883540 12883933 12884068 12884542 12884876 12884901 12882613 12882785 12882886 12882922 12883048 12883745 12884380 12884448 12884798 12884836 12884905 12884909
scaffold_8, genome position

dd

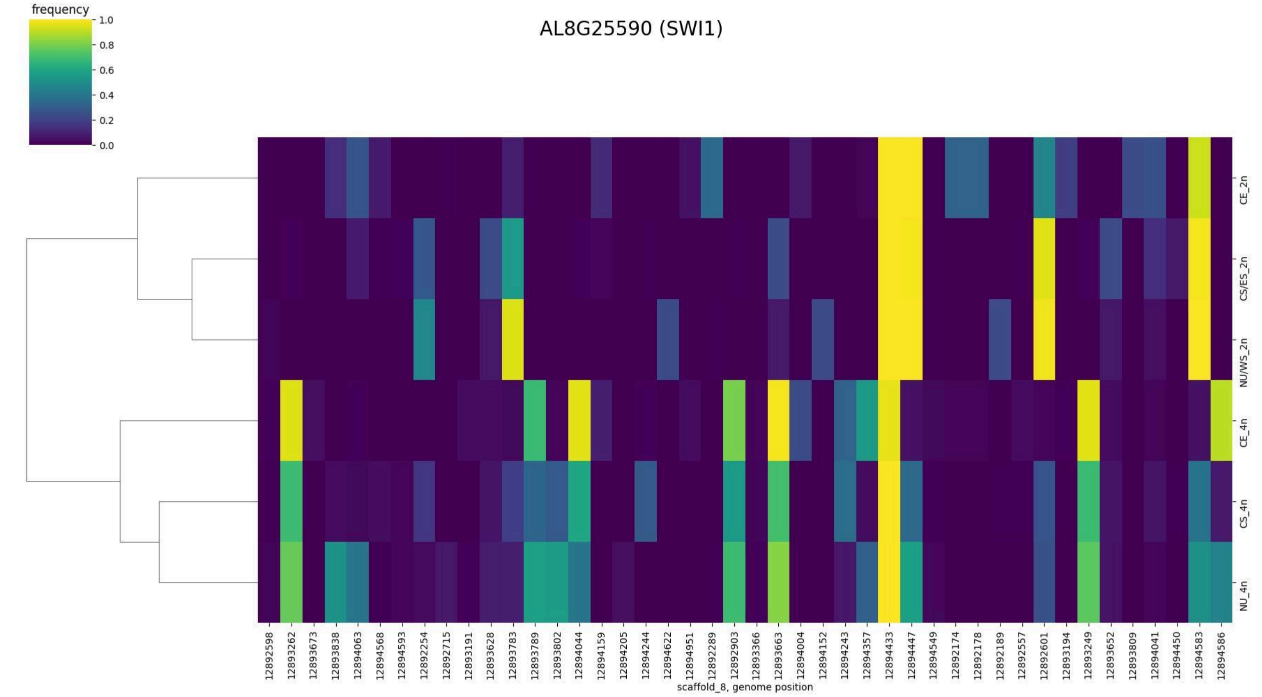
AL8G25590 (SWI1)
frequency
1.0
0.8
0.6
0.4
0.2
0.0
CE_2n
CS/ES_2n
NU/WS_2n
CE_4n
CS_4n
NU_4n
12892598 12893262 12893673 12893838 12894063 12894568 12894593 12892254 12892715 12893191 12893628 12893783 12893789 12893802 12894044 12894159 12894205 12894244 12894622 12894951 12892289 12892903 12893366 12893663 12894004 12894152 12894243 12894357 12894433 12894447 12894549 12892174 12892178 12892189 12892557 12892601 12893194 12893249 12893652 12893809 12894041 12894450 12894583 12894586
scaffold_8, genome position

ee

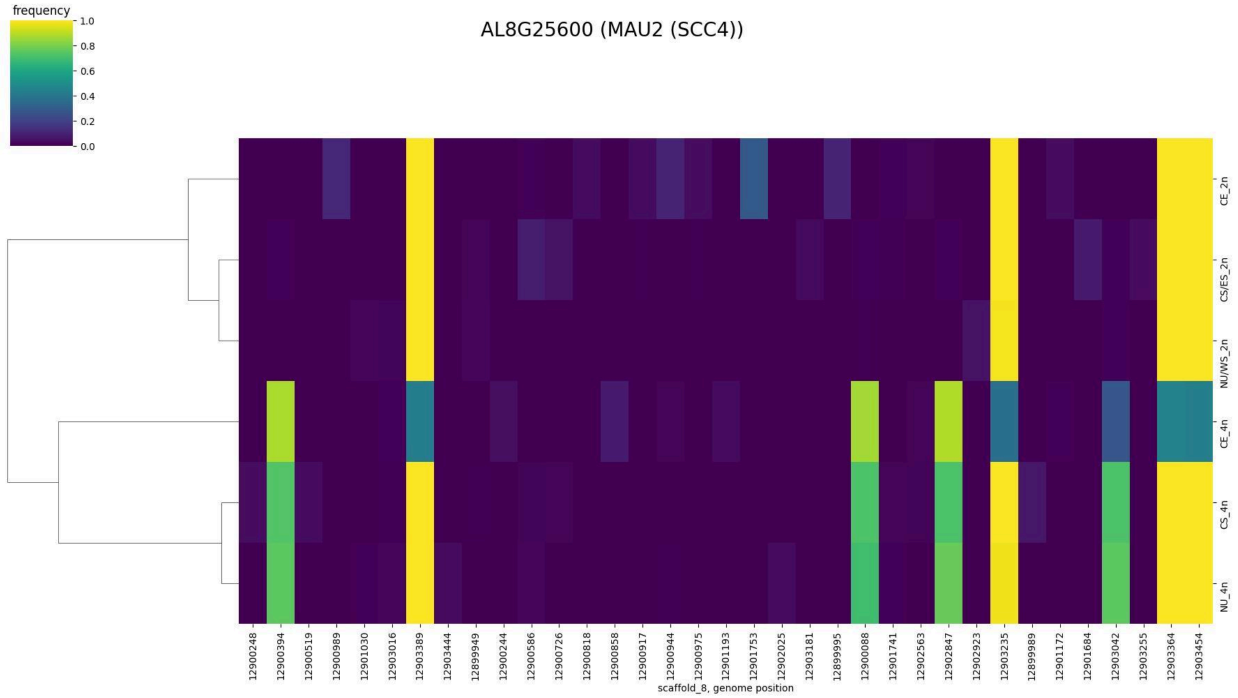
AL8G25600 (MAU2 (SCC4))
frequency
1.0
0.8
0.6
0.4
0.2
0.0
CE_2n
CS/ES_2n
NU/WS_2n
CE_4n
CS_4n
NU_4n
12900248
12900394
12900519
12900989
12901030
12903016
12903389
12903444
12899949
12900244
12900586
12900726
12900818
12900858
12900917
12900944
12900975
12901193
12901753
12902025
12903181
12899995
12900088
12901741
12902563
12902847
12902923
12903235
12899989
12901172
12901684
12903042
12903255
12903364
12903454
scaffold_8, genome position

ff

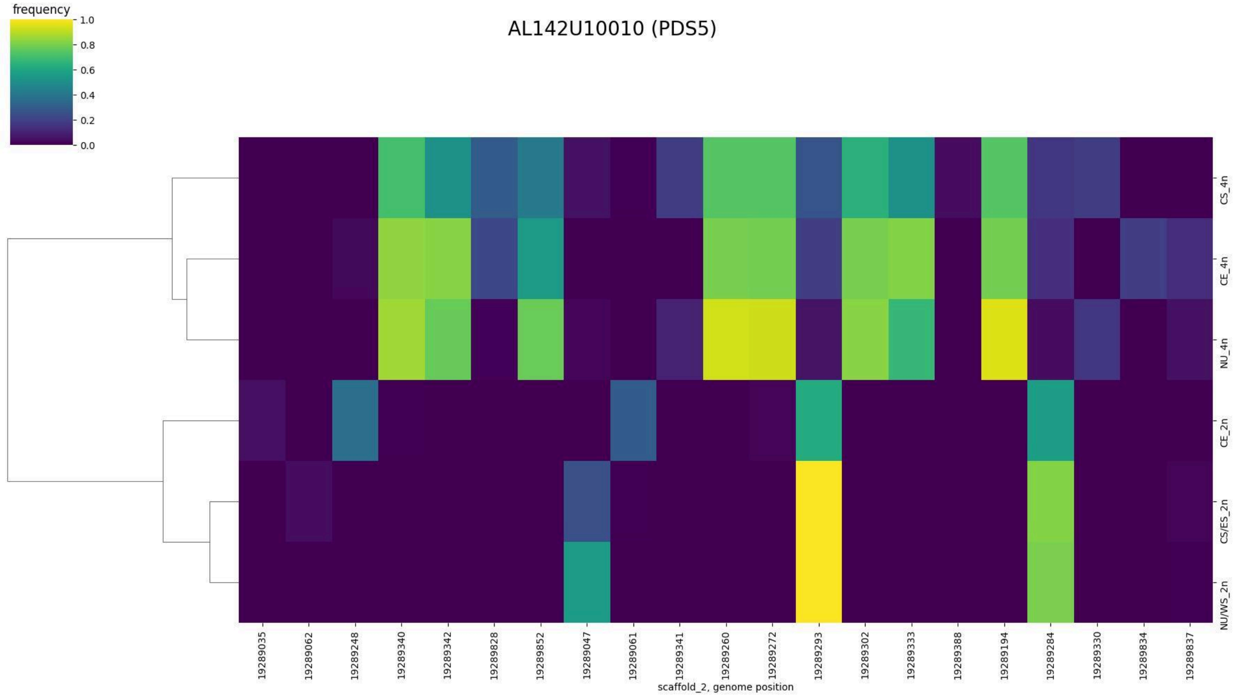
AL142U10010 (PDS5)
frequency
1.0
0.8
0.6
0.4
0.2
0.0
CS_4n
CE_4n
NU_4n
CE_2n
CS/ES_2n
NU/WS_2n
19289035
19289062
19289248
19289340
19289342
19289828
19289852
19289047
19289061
19289341
19289260
19289272
19289293
19289302
19289333
19289388
19289194
19289284
19289330
19289834
19289837
scaffold_2, genome position

gg

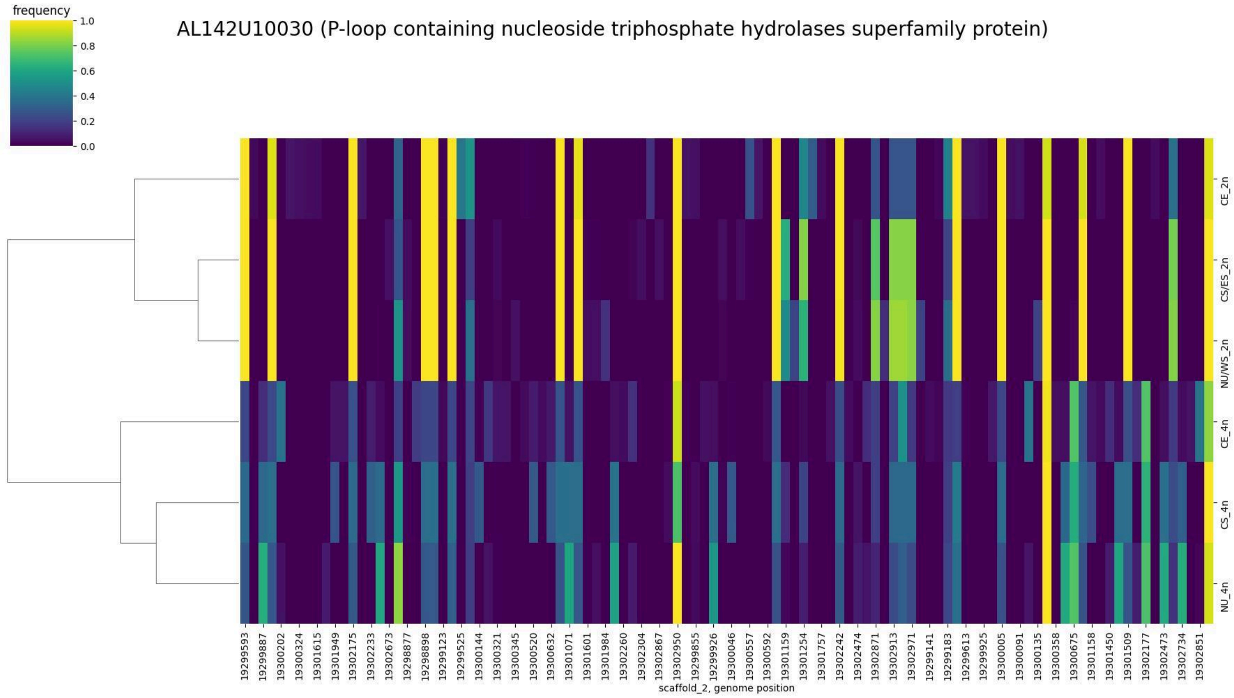

Supplementary Figure 12. Biallelic SNP frequency heatmap for each introgressed gene. Hierarchical clustering based on distance shown on the left side of the heatmap.

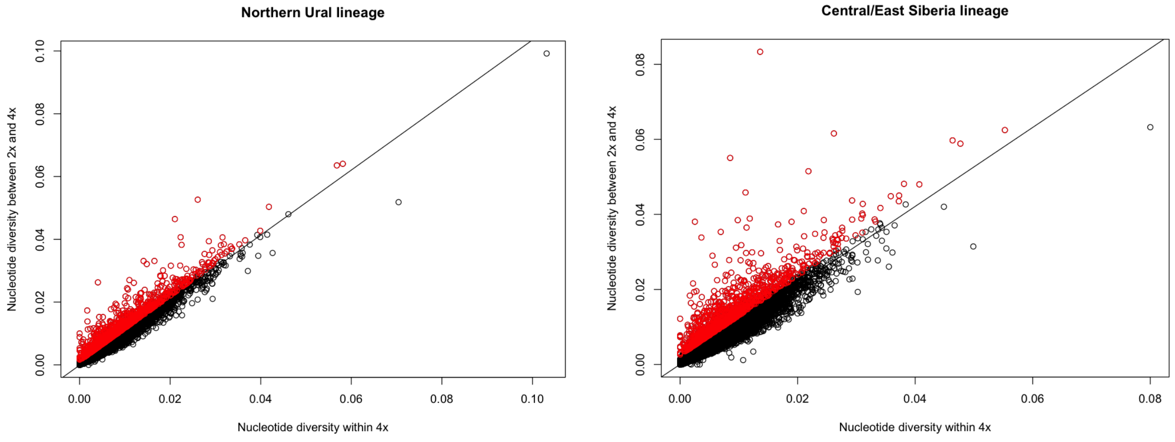

Supplementary Figure 13. Nucleotide diversity within tetraploids (π) and between tetraploids and diploids (dxy). Nucleotide diversity is calculated per-gene, so that each dot represents a single gene. Red points are genes with higher diversity between diploids and tetraploids (dxy) than nucleotide diversity (π) within tetraploids. Line shows linear regression. Both plots were used for residual analysis shown in Supplementary Figure 13.

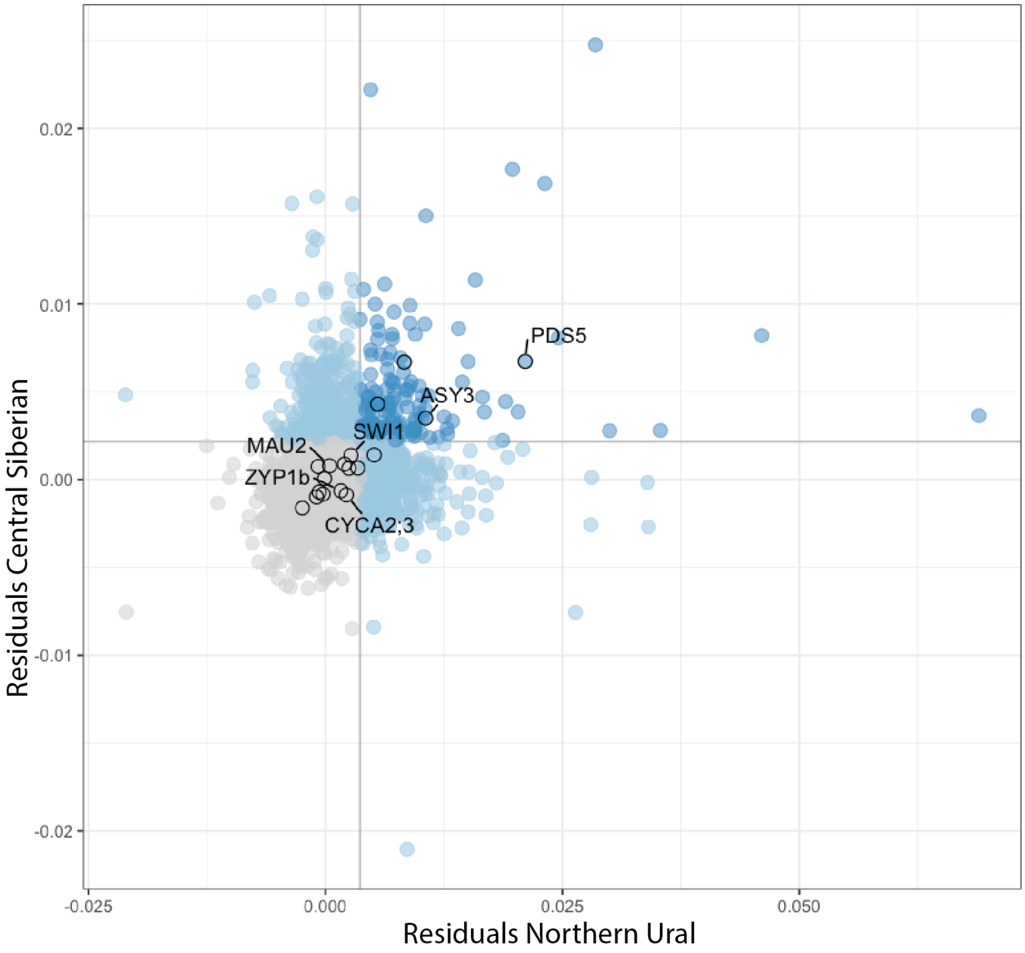

Supplementary Figure 14: Residuals from analyses in Supplementary Fig. 10. X axis are residuals in the Northern Ural tetraploid vs West Siberian diploid analysis, Y axis residuals from Central Siberian tetraploids vs Eastern Siberian diploid analysis. Grey points are genes below significance threshold in both analyses, light blue dots are above significance threshold in one analysis, and dark blue points are genes above the significance threshold in both analyses. Black circle points are genes within introgression windows, with meiotic genes labeled.

Supplementary Table 1 - Per-lineage calculations of pi, per-chromosome and averaged across the genome.

lineage	scaffold 1	scaffold 2	scaffold 3	scaffold 4	scaffold 5	scaffold 6	scaffold 7	scaffold 8
A. pedemontana	0.00361107	0.0038878	0.00378414	0.00393642	0.00417384	0.00323467	0.00412342	0.00429241
Northern Ural 2x	0.00393875	0.00453203	0.00360437	0.00409764	0.00439911	0.00351335	0.00415009	0.00436425
Central Europe 2x	0.00468863	0.00568001	0.00457219	0.00507816	0.00535714	0.00451433	0.00528225	0.00534325
Northern Ural 4x	0.00427826	0.00480552	0.00410252	0.00446104	0.00465438	0.00394057	0.00472856	0.00486608
East Siberia 2x	0.00351382	0.00439513	0.0036366	0.00391632	0.00419931	0.00354756	0.0041359	0.00426257
Central Europe 4x	0.00641077	0.00716033	0.00640866	0.00647403	0.00680791	0.00581352	0.00694464	0.0067454

Central Siberia 2x	0.00399562	0.00501767	0.00399346	0.00437062	0.00461261	0.00392471	0.00469372	0.00462979
KIS 2x	0.00327073	0.00419069	0.00332404	0.00364323	0.00356609	0.00302025	0.00385952	0.0038599
East Siberia SC	7.10E-04	7.81E-04	5.19E-04	4.61E-04	4.36E-04	4.48E-04	6.04E-04	6.71E-04
Central Siberia 4x	0.00395844	0.00476065	0.00399478	0.00432145	0.00460887	0.00389787	0.00456974	0.00450748
West Siberia 2x	0.00334681	0.00436906	0.00332073	0.00372323	0.00369768	0.00320731	0.00398848	0.00399662
A. kamchatica	0.00736282	0.0073324	0.00719772	0.00720103	0.00765063	0.00657111	0.00773628	0.00728415
North America 2x	0.00291611	0.00340514	0.00271469	0.00290837	0.00316839	0.00250697	0.00295552	0.00348114
Karelia 2x	0.00222865	0.00280702	0.00226579	0.00269737	0.00276696	0.00204716	0.00248076	0.00284813
A. cebenensis	0.00228218	0.00231461	0.00208466	0.00242119	0.00273483	0.00206561	0.00243594	0.00247587
Scandinavia UK 2x	0.00383055	0.00482535	0.00383085	0.00404411	0.00463489	0.00364449	0.00455976	0.00458655
Amur Basin 2x	0.00391834	0.00481927	0.0040495	0.00415709	0.00456762	0.00365384	0.00446464	0.00451941

Supplementary Table 2. Per-lineage calculations of Tajima's D, per-chromosome and averaged across the genome.

lineage	scaffold 1	scaffold 2	scaffold 3	scaffold 4	scaffold 5	scaffold 6	scaffold 7	scaffold 8
A. pedemontana	0.351836	0.127919	0.379832	0.224598	0.311647	0.265445	0.291054	0.298255
Northern Ural 2x	-0.189034	-0.307565	-0.404475	-0.205345	-0.222719	-0.341508	-0.397337	-0.285788
Central Europe 2x	-1.12589	-1.08278	-1.01791	-1.09279	-1.12408	-1.12502	-1.06466	-1.11581
Northern Ural 4x	-2.22996	-2.23336	-2.281	-2.20619	-2.25325	-2.27627	-2.20664	-2.2255
East Siberia 2x	-1.84527	-1.81637	-1.81096	-1.79413	-1.79515	-1.82369	-1.7341	-1.71455
Central Europe 4x	-1.47644	-1.46292	-1.50471	-1.52655	-1.49396	-1.51741	-1.45641	-1.54091
Central Siberia 2x	-0.937088	-0.920773	-1.02426	-0.898363	-0.897925	-0.947642	-0.894733	-0.910017
KIS 2x	0.255721	0.0949487	0.027569	0.115456	0.0222048	0.00571544	0.0600888	0.128294
East Siberia SC	-0.74389	-1.21662	-1.43283	-1.4196	-1.60667	-1.37102	-1.06109	-1.32452
Central Siberia 4x	-2.03433	-1.99091	-2.027	-1.98798	-1.98075	-1.99948	-1.96143	-1.95184
West Siberia 2x	-1.83802	-1.72012	-1.82922	-1.78196	-1.868	-1.8787	-1.78363	-1.7811
A. kamchatica	1.96023	1.73526	2.04855	1.91818	1.87188	2.03841	1.8983	1.81561
North America 2x	0.0974186	0.00982349	0.167607	0.173932	0.143156	0.132193	0.151343	0.0416878
Karelia 2x	0.611991	1.01245	0.89406	0.972478	0.720812	0.571197	0.493578	0.885834
A. cebenensis	0.630147	0.499044	0.486182	0.455501	0.641176	0.712201	0.475939	0.508728
Scandinavia UK 2x	-0.613977	-0.593124	-0.56832	-0.589315	-0.591939	-0.594088	-0.447816	-0.474843
Amur Basin 2x	-0.462973	-0.567186	-0.537812	-0.599666	-0.477144	-0.553145	-0.520671	-0.516953

Supplementary Table 3 - Model comparison from demographic modeling, corresponding to models in Supplementary Figure 10. Model with best AIC score highlighted in green.

Model	MaxEstLhood	MaxObsLhood	deltaL	AIC
a	-3584828.033	-3524513.384	60314.649	16511737.92
b	-3584905.563	-3524513.384	60392.179	16512095.02

c	-3558869.191	-3524513.384	34355.807	16392171.49
d	-4334994.199	-3524513.384	810480.815	19967003.28
e	-3578195.633	-3524513.384	53682.249	16481189.09
f	-3559080.049	-3524513.384	34566.665	16393146.71
g	-3560767.578	-3524513.384	36254.194	16400919.46
h	-3584907.559	-3524513.384	60394.175	16512108.22

Supplementary Table 4. Parameter estimates and confidence intervals for demographic model c (see Supplementary Figure 10).

	ES 2x popsize	CS 4x popsize	WS 2x popsize	NU 4x popsize	ANC popsize1	ANC popsize2	ANC popsize3	TDIV1	TDIV2	TDIV3
	94608	3011	4500	659670	4203959	4452069	1767832	4025	4734	29022
**2.5% CI**	58504	2708	3622	608925	3849987	4111756	1108907	3825	4625	19326
**97.5% CI**	130713	3313	5377	710415	4557931	4792383	2426757	4227	4843	38719

Supplementary Table 5. Parameter estimates and confidence intervals for demographic modeling with Central European and Northern Ural polyploids. TDIV1 is divergence time of Siberian tetraploids and local diploids, while TDIV2 is divergence time of Central European tetraploids and local diploids.

	CE 2x popsize	CE 4x popsize	WS 2x popsize	NU 4x popsize	ANC popsize1	ANC popsize2	ANC popsize3	TDIV1	TDIV2	TDIV3
	247217	250345	516868	1086373	1974881	21301841	2974421	4755	32124	358346
**2.5% CI**	199772	203444	391178	966978	1448171	17799071	2403827	3788	26419	300287
**97.5% CI**	294663	297246	642558	1205768	2501591	24804612	3545015	5722	37829	416406

Supplementary Table 6 - Published and assembled S-alleles used in this study.

S-allele	Corresponding name	Accession	Reference
H2018	CgrSRK45	MT592980.1	Neuffer et al., 2023
H3009	AhSRK30	EU878015.1	Castric et al., 2008
H3012	CgrSRK11	MT592933.1	Neuffer et al., 2023
H3014	CgrSRK19	MT592944.1	Neuffer et al., 2023
H3021	-	-	supplementary data 3
H3024	-	-	supplementary data 3
H4021	CgrSRK56	MT592996.1	Neuffer et al., 2023
H4031	CgrSRK28	MT592956.1	Neuffer et al., 2023
H4042	-	-	supplementary data 3
H4043	-	-	supplementary data 3
