# Supplementary figures and images for: Multiple Autopolyploid Arabidopsis lyrata Populations Stabilized by Long-Range Adaptive Introgression Across Eurasia
Source: Mol Biol Evol. 2025 Jul 24;42(8):msaf153. doi: 10.1093/molbev/msaf153 (PMC12342985; doi:10.1093/molbev/msaf153)

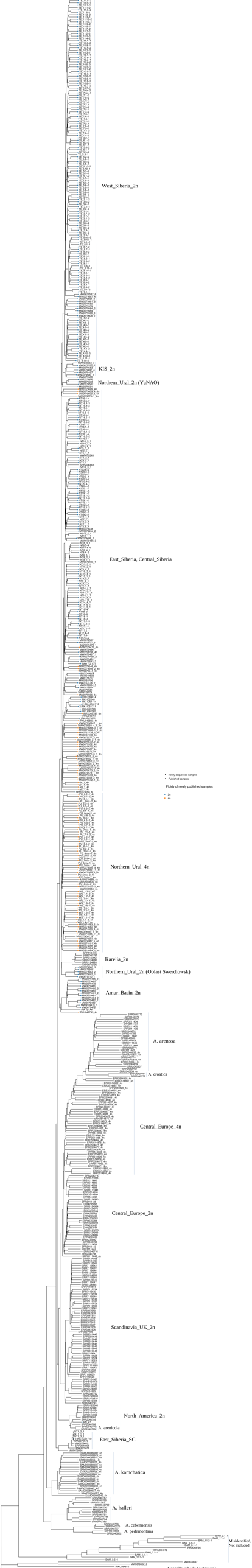

Supplement: msaf153_Supplementary_Data [file msaf153_supplementary_data.zip › SupplementaryFigure5.pdf]
